# Deep Learning Network‐Tailored Microenvironment Matching of 4D Bioprinting Bioactive Scaffolds for Bone Regeneration

**DOI:** 10.1002/advs.76351

**Published:** 2026-06-30

**Authors:** Xiongjie Liang, Yuechi Zhang, Weifeng Hu, Shiyan Lv, Fan Jia, Yan Zhang, Feng Wu, Changjiang Yang, Guanqi Zhen, Jinglong Yan, Xu Cui, Wei Zhao, Guanghua Chen

**Affiliations:** ^1^ Department of Orthopedics Second Affiliated Hospital of Harbin Medical University Harbin China; ^2^ Department of Astronautical Science and Mechanics Harbin Institute of Technology (HIT) Harbin China; ^3^ Department of PET/CT‐MR Harbin Medical University Cancer Hospital Harbin Heilongjiang Province China; ^4^ Department of Oncology Second Affiliated Hospital of Harbin Medical University Harbin China; ^5^ Shenzhen Key Laboratory of Marine Biomedical Materials CAS‐HK Joint Lab of Biomaterials Shenzhen Institutes of Advanced Technology Chinese Academy of Sciences Shenzhen China

**Keywords:** 4D printing, bioactive glass, bioactive scaffold, bone regeneration, deep learning network

## Abstract

Pathological microenvironments linked to aging, trauma, malignancies, and metabolic disorders significantly hinder bone fractures and frequently result in fracture nonunion, posing substantial worldwide clinical difficulties. Widely prevalent therapies encounter difficulties in addressing diverse anatomical defects and variable illness conditions due to their inflexible designs and limitations in empirical optimization. Efficient strategies are critical to restore mechanics, improve pathological microenvironments, enhance neovascularization, and adapt to anatomical defects and clinical conditions. Deep learning networks (DLN) excel at analyzing extensive nonlinear relationships, enabling predictions of biomaterial‑biological interactions, hence accelerating biomaterial development. This study presents a synergistic DLN and 4D printing approach to fabricate a microenvironment‐adaptive bioactive scaffold (MABS) for enhanced osteogenesis and angiogenesis. The scaffold integrates bioactive glass and a shape‐memory PgP matrix, with a multilayer perceptron (MLP) neural network optimizing its design via nonlinear parameter‐performance analysis. In vivo investigations revealed that the DLN‐optimized scaffold enhanced shape‐morphing adaptability and promoted the formation of dense bone tissue and vascular networks. This paradigm shift—employing DLN to integrate 4D printing dynamics, degradation kinetics, and multi‐scale biological responses—transforms bone implants from static entities to dynamically adaptive systems, offering a scalable, intelligent framework for precise bone repair that rectifies the deficiencies of current strategies.

## Graphical abstract

A DLN dataset was built to analyze MABS composition versus in vitro/in vivo osteogenesis and angiogenesis. An MLP neural network, taking BG morphological parameters as input, extracts bioactive features from these datasets. A rabbit tibial defect model then validates 4D‐printed MABS for adaptability and bone regeneration in critical defects.

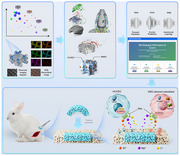

## 1 Introduction

Globally, the incidence of bone injury is on a continuous rise, particularly for open fractures and segmental bone defects. This trend is mainly attributable to population aging, traffic accidents, sports injuries, infections, tumor resections, and sudden public disasters [1, 2, 3]. Open fractures and segmental bone defects, often associated with marked bone loss and severe circulatory compromise, may disrupt osteogenesis‐osteolysis balance, hinder fracture healing, and eventually cause non‐union in later stages, thus drastically reducing patients’ quality of life and even leading to mortality [4, 5, 6]. Autologous bone is recognized as the gold standard for bone repair, yet its clinical application is constrained by multiple factors, such as the limited availability for massive bone loss and the risk of donor‐site complications. Therefore, researchers have focused on developing various biological substitutes. As a major inorganic component of bone tissue, bioactive glass (BG) has garnered extensive attention as a new‐generation calcium‐phosphate‐based biomaterial. Nevertheless, bio‐ceramics (including BG) still face numerous challenges: for instance, the vitrification characteristic after post‐treatment leads to a mismatch in hardness with natural bone tissue, and the shape compatibility with the implantation site requires further optimization. The bone repair process is a complex physiological event involving multiple mechanisms and factors, with abundant nonlinear correlations among these regulatory elements [7, 8, 9]. It is a functional trait ensemble rather than the outcome of regulation by a single factor. Thus, the pro‐biological activity of BG and its related interaction mechanisms remain to be further explored. Although extensive and in‐depth investigations have been conducted on the various biological functions of BG, the crosstalk and comprehensive effects among these functional aspects have not been thoroughly elucidated or established. Meanwhile, apart from the micro‐macro morphological studies of biomaterials, the biological effects of the micromorphology of BG particles themselves have not been systematically investigated.

Although various methodologies can improve the composition and structural design and customize the degradation kinetics and mechanical compatibility of implants to effectively modulate the pathological microenvironment [10, 11], advanced material fabrication techniques must be incorporated to fabricate implants with appropriate macro/micro morphologies that conform seamlessly to the complex shapes of bone fractures and defects for clinical application [12, 13]. As a programmable intelligent material, shape memory polymer (SMP) has the functions of programmable self‐recovery and self‐adaptation, which makes it a competitive candidate material for the fabrication of reconfigurable scaffolds for the treatment of irregular bone defects. Meanwhile, 4D printing technology, developed through intelligent programmable materials like SMP and 3D printing, conceptually integrates 3D printing with time‐controlled shaping and mechanical strength of scaffolds. This enables the production of reconfigurable bone tissue engineering scaffolds for treating irregular bone defects; it provides a new opportunity for the development of this material in the biomedical field [14]. The 4D‐printed shape memory scaffolds can be molded into irregular bone defects at their transition temperature (Tg) [15]. However, in many studies on SMP based bone repair scaffolds with Tg close to body temperature cannot provide sufficient mechanical strength for the scaffold in the bone regeneration process [16], while the shape memory polymers (SMPs) with Tg too high will cause burns to surrounding tissues and loss of biological activity of the loaded biomolecules. Therefore, there is an urgent need for SMPs with appropriate Tg that can temporarily avoid burns to surrounding tissues/cells and have sufficient mechanical strength at body temperature to produce reconfigurable scaffolds with desired deformation/shape recovery ability and excellent mechanical support during bone regeneration. An experimental study on bone induction after heat treatment demonstrated that femoral cortical bone from rats heated in a 70°C water bath for 1 h showed preserved osteo‐inductive activity. Eleven days after transplantation into rat muscles, mRNA induction of alkaline phosphatase and type I/II collagen was observed, confirming the bone induction capability. However, when the bone was treated at 90°C or higher temperatures, the damaging effects on bone tissue became significant [17]. Therefore, for SMPs used in repairing irregular bone defects, when the glass transition temperature (Tg) is controlled below 70°C, the polymer scaffold can not only provide the required strength for support, but also avoid irreversible damage to bone tissue during implantation. Recent advancements in 4D printing technology, which integrates SMPs with bioactive materials, show the transformative promise of adaptive bone repair scaffolds created to dynamically conform to defect geometries [16, 18]. For example, BG has emerged as a promising candidate due to its hydroxyapatite (HA) mineralization capacity and sustained release of osteogenic ions (e.g., Sr^2^^+^ and Ca^2^^+^) [19, 20]. However, SMPs such as poly(D,L‐lactic acid)‐graft‐polyethylene glycol (PgP) often lack bioactivity, and their application is accompanied by the risk of an inflammatory response due to their acidic degradation byproducts [21, 22]. While composite materials (e.g., BG@PgP) aim to synergize mechanical adaptability with bioactivity, their optimization remains hindered by the complex, nonlinear relationships among material composition, microstructure, and biological performance [23, 24]. Nevertheless, few existing machine learning frameworks integrate 4D printing dynamics, degradation kinetics, and multiscale biological responses to guide scaffold design—a gap critical for addressing irregular bone defects.

Therefore, to obtain BG‐based biofunctionalized biomimetic materials with definite efficacy and clear functional orientation, an integrated prediction system that combines experiments and in‐depth analyses of the regulatory factor trait ensemble—encompassing BG micromorphology, characterization, biological mechanisms, and biological functions—is of critical significance for the preclinical application of such novel biomimetic materials. Despite the various clinical interventions aimed at restoring the mechanical stability and structural integrity of bone fractures or defects, a single type of biomaterial or a biomaterial with a simple composition is insufficient to provide optimal adaptation to defects in different anatomical regions and distinct pathological microenvironments. Moreover, commonly used conformation designs, often driven by empirical parameter adjustments, do not address the dynamic interplay between material properties, microenvironmental cues, and tissue regeneration—a challenge exacerbated by the lack of standardized evaluation systems for nonlinear biological responses. Hence, tuning the composition and morphological characteristics of implants by utilizing various materials to adapt to different fracture and bone defect morphologies while regulating local physicochemical and physiological fundamentals to create an optimal osteogenic microenvironment represents a significant technical challenge for pathological bone tissue regeneration and repair applications.

The emergence of Artificial Intelligent (AI), particularly deep learning network, has significantly transformed the frontier concepts of fundamental scientific research, and concurrent enhancements in computational capabilities have allowed outputs to be obtained efficiently in less time [25, 26]. Protein structures can be efficiently and accurately predicted through the use of AI or deep learning, facilitating the development and subsequent application of many protein‐based drugs [27, 28]. With the improvement in the quality of AI for science, data‐driven AI deep learning has progressively become integrated into materials science, enabling multidimensional and precise predictions of the relationships between material composition/structure and performance, as well as the interactions between materials and the complex cell/tissue microenvironment post‐implantation [29, 30, 31]. For example, Watson et al. employed diffusion‐based neural networks for the de novo design of functional proteins, highlighting AI's capacity to resolve the intricate structure‒function relationships of protein‐based materials [32]. Moreover, Zhou et al. developed machine learning models to predict the fate of stem cell lineages on biomaterials for bone tissue engineering applications, underscoring the potential of AI to bridge material properties and biological outcomes [33].

**FIGURE 1:**
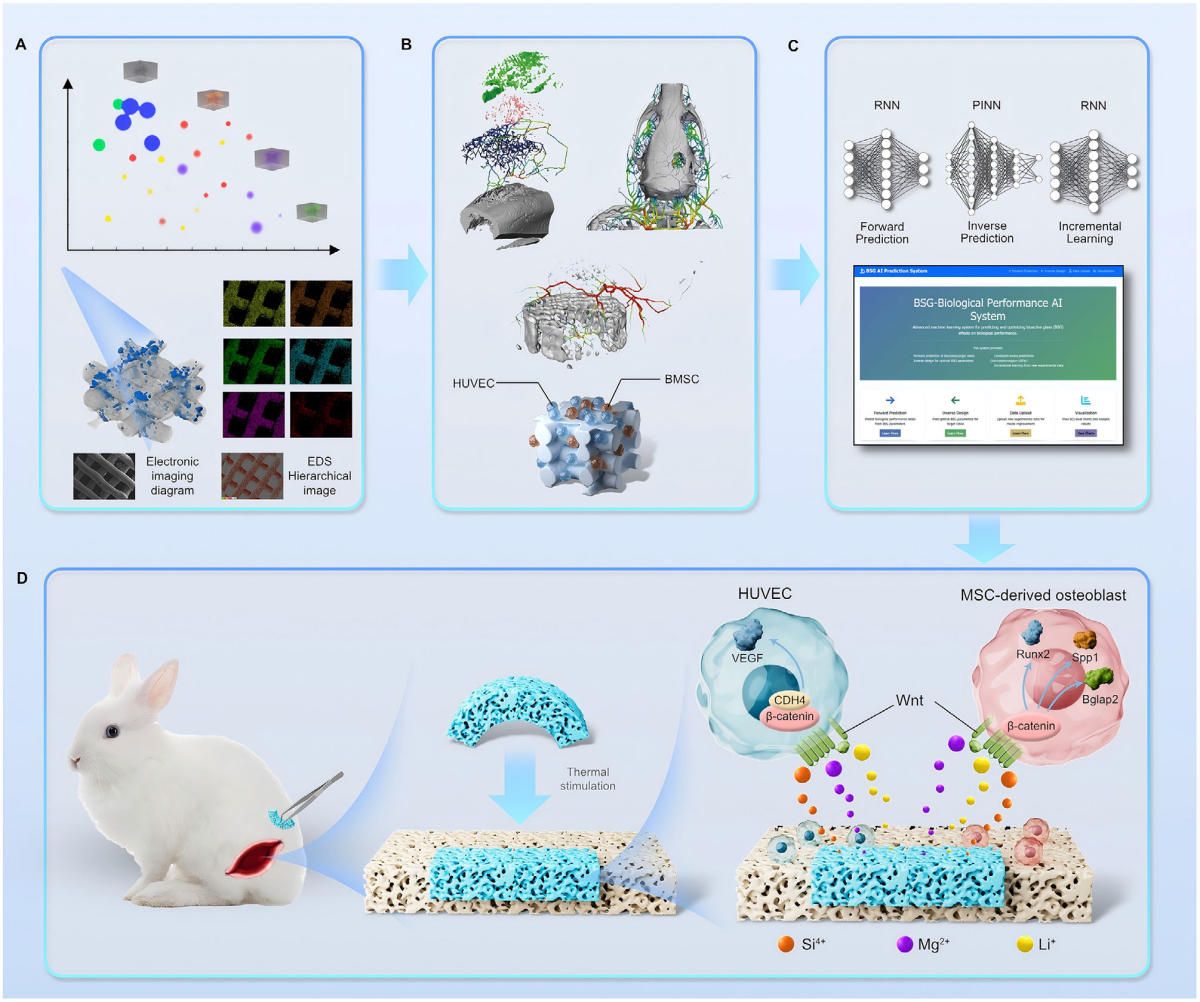
(A) Construction of a DLN datasets for analyzing the interaction between composition of MABS and their biological performances in materials science. (B) Construction of a deep learning network dataset for analyzing the interaction between composition of MABS and the in vitro and in vivo performances of osteogenesis and angiogenesis. (C) Schematic diagram illustration of the training and selection procedures of a multilayer perceptron neural network, wherein the input layer comprises multiple morphological parameters of BG. The multilayer perceptron extracts the bioactive characteristics of implants from datasets acquired through in vitro osteogenesis and angiogenesis data, as well as in vivo experimental data. (D) Establishment of a tibial defect model in New Zealand rabbits to validate the self‐adaptability of 4D‐printed MABS, as well as their mechanical support, biocompatibility, and efficacy in bone regeneration in the presence of substantial bone defects.

Herein, we present a 4D‐printed microenvironment‐adaptive bioactive scaffold (BG@PgP, MABS), a DLN‐driven design paradigm tailored to the dynamic mechanical and physiological microenvironments of bone defects, as schematically illustrated in Figure 1. By combining BG with a shape‐memory PDLLA‐g‐PEG matrix, this specific bioactive scaffold is endowed with temperature‐triggered geometric adaptation capabilities. A modified MLP neural network decoding engine was utilized to decode nonlinear correlations between BG topological parameters (area fraction (AF), anisotropy (Ani), equaldiameter (EqD) and Eccentricity (Ecc)) and scaffold performance, thereby optimizing mechanical strength, ion release kinetics, and osteogenic and angiogenic activity of the implants, as illustrated in Figure 1A. Validated in rabbit tibial defect models, this method significantly enhances osteogenic activity (bone volume fraction increased from 9.7% ± 3.2% in the control group to 40.4% ± 3.6% in the DLN‐optimized group, *p* < 0.001) and promotes vascular density (a 1.4‐fold increase in vascular branches, *p* < 0.01) compared to the non‐optimized PgP control, demonstrating outstanding bone regeneration efficacy. Overall, this study aims to propose an extensible intelligent design framework for precise bone repair, transforming biomaterial design from static Broussonetia papyrifera structures to dynamic self‐adaptive systems.

## 2 Results

### 2.1 Acquisition of the Original Dataset for the 4D‐Printed MABS

Data preprocessing is essential to ensure the quality and dependability of future analyses [34]. The initial dataset includes micromorphological parameters (AF, Ani, EqD, and Ecc), physicochemical characteristics (mechanical strength, porosity, shape memory performance, EDS, ICP‐AES, element constitution, and PH value), and biological data (Biocompatibility, osteogenesis in vitro, angiogenesis in vitro, osteogenesis in vivo, and angiogenesis in vivo) from both in vivo and in vitro studies of BG and inevitably contains missing values. To address this problem, this study implements a strategy of randomly inputting missing values from the same data columns; this approach is founded on the idea that values within the same columns may share similar statistical properties. Randomly selecting from the existing values in a particular column reduces the risk of bias while maintaining the overall data distribution. Column‐based random imputation suits datasets with low missing rates below 5%. This method yields negligible bias and boasts high computational efficiency [35]. Furthermore, this effectively prevents issues such as inaccurate model parameter estimation and instability during training that can result from missing data, thus improving the dataset's completeness. Next, data normalization is carried out by standardizing the data using Z scores via the following Equation (1).

(1) $$Z=\frac{\chi -\mu }{\sigma }$$

Each data point within the dataset should be transformed, where χ represents the original data point, μ denotes the mean of the dataset, and σ signifies the standard deviation. This process of standardization proves to be particularly effective in the context of training neural networks. Deep learning networks predominantly utilize gradient‐based optimization algorithms, and the presence of features with significantly disparate scales can result in excessively large or small gradients. Such discrepancies may hinder the convergence process, potentially leading to slow convergence rates or even divergence during training. By standardizing the data, all the features are normalized to a consistent scale, which facilitates a more efficient learning process for the model. Following standardization, each feature contributes equally to the model's learning, thereby enhancing both training efficiency and the speed of convergence.

The particle morphology parameters utilized in the BG framework include the area fraction (AreaFraction, AF), equivalent diameter (EqDiameter, EqD), eccentricity (Eccentricity, Ecc), and anisotropy (Anisotropy, Ani). These parameters represent fundamental characteristics that are vital to the research objectives. To mitigate the risk of mishandling or altering essential data, the program adopts a highly conservative stance regarding these parameters by explicitly prohibiting any modifications. This approach safeguards the integrity of these critical features throughout the data processing workflow, as any changes to these key characteristics could introduce errors or result in misleading conclusions in subsequent analyses.

#### 2.1.1 Obtaining the BG Particle Morphology Parameters in the 4D‐Printed MABS

**FIGURE 2:**
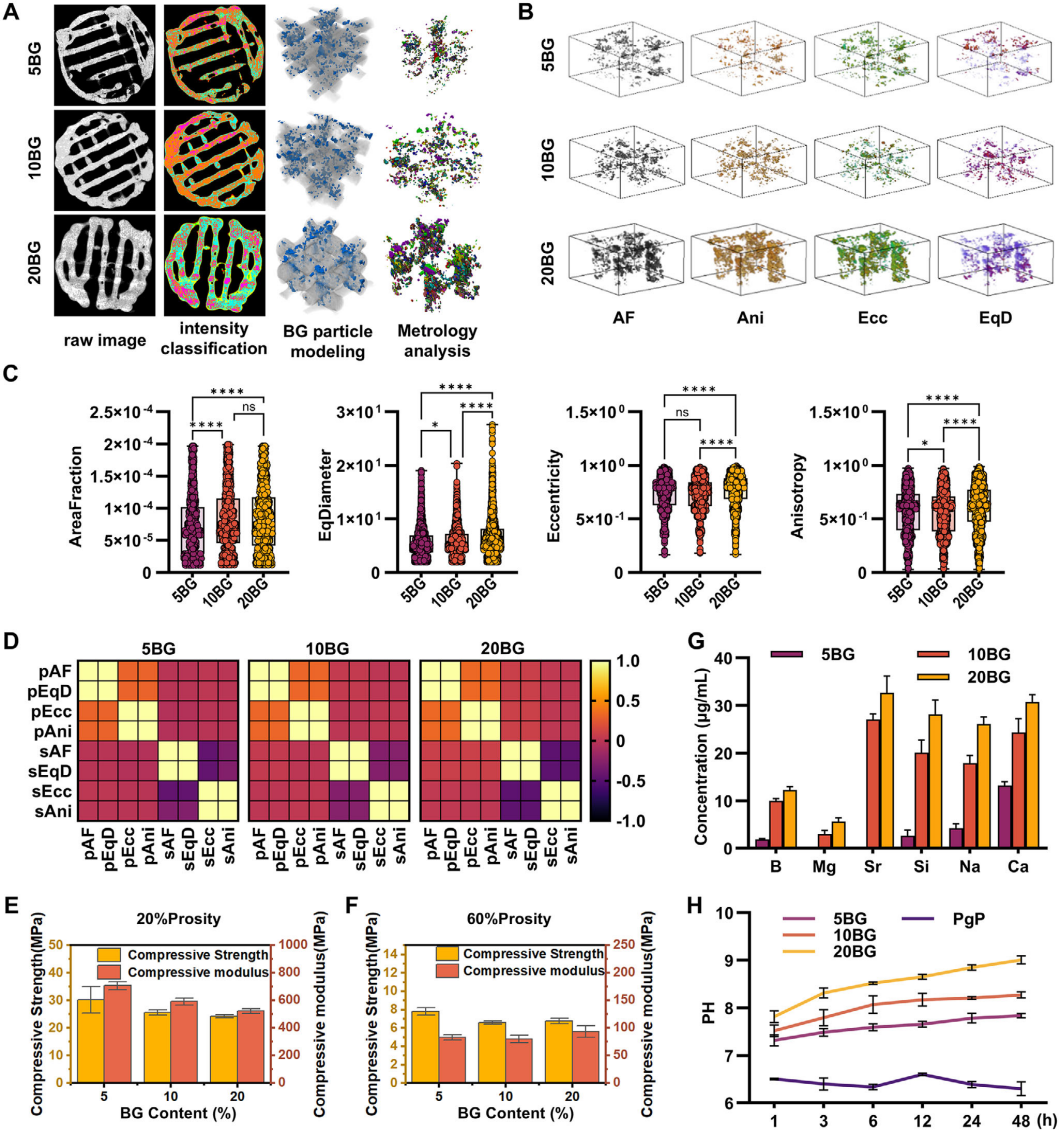
(A) Procedures for deep learning‐derived analysis of the distribution of BG particles within the truss structures of 4D‐printed MABS at varying concentrations of BGs. (B) Three‐dimensional simulation of the four morphological parameters relating to the distribution of BGs within the truss structures of MABS, including Area Fraction (AF), Eq diameter (EqD), Ec centricity (Ecc), Anisotropy (Ani), as output from deep learning. (C) The simulating results of four morphological parameters (AF, EqD, Ecc, Ani) relating to the distribution of BGs within the truss structures of MABS. (D) Pearson's r correlation analysis regarding the impact of BGs’ particle morphological attributes on scaffold architecture. (E, F) Compressive strength and modulus of MABS following different porosities of 60% and 20%. (G) ICP–AES detecting the elemental composition of MABS. (H) variations in interfacial pH values of MABS as a function of immersion time in PBS. Data are presented as means ± SD, *n* = 3, Statistical significance was determined using the one‐way ANOVA method with Tukey's multiple comparisons tests, Statistical significance was defined as *p < 0.05, **p < 0.01, ***p < 0.001, and ****p < 0.0001, whereas nsP > 0.05 was deemed not statistically significant.

As illustrated in Figure 2A, cross‐sectional thin‐layer scanning of the 4D‐printed MABS was conducted by micro‐CT. Following the acquisition of the micro‐CT data, computer‐aided techniques were employed to reconstruct the structural features, yielding a three‐dimensional (3D) representation of the scaffold and the weight parameters associated with the morphological characteristics of the BG particles at varying concentrations within the scaffold. Pseudo‐coloring techniques were utilized to distinguish the BG particles according to the particle size and aggregation degree. The volumes of both the BG particles and the scaffold were then reconstructed to visualize the spatial distribution of the BG particles within the scaffold. Each BG particle was assigned a unique color, and additional morphological parameter detection was performed for subsequent analysis. Figure 2B presents a visualization of various morphological parameters of BG particles at different concentrations following preprocessing. Each visualized parameter was designated with specific pseudo‐coloring, where darker hues correspond to higher parameter values and lighter hues indicate lower values. Notably, as shown in Figure 2B, an increase in the BG content within the scaffold correlated with an increasing trend in the morphological parameters of the BG particles. Then, statistical calculations were executed on the data derived from Figure 2A,B. The bar chart presented in Figure 2C illustrates the quantitative analysis of particle morphological parameters across scaffolds with different BG concentrations. A statistical analysis was performed on the particle values at various BG concentrations to depict their distribution ranges. Figure 2D presents the results of Pearson's r correlation analysis for the different datasets. The three matrix plots correspond to scaffolds with varying BG contents. The parameters prefixed with “*p*” denote the microscopic characteristics of the BG particles, whereas those prefixed with “*s*” represent the macroscopic characteristics of the scaffold. Correlation analysis elucidates the impact of the microscopic particles on the macroscopic structure of the scaffold, thereby enabling further investigation of potential relationships through deep learning methodologies.

#### 2.1.2 Physicochemical Properties of 4D‐Printed MABS

To confirm the self‐mineralization capability of the 4D‐printed MABS, scaffolds with different ratios of BG were immersed in simulated body fluid (SBF) for 3, 7, and 14 d and then characterized by X‐ray diffraction (XRD). The XRD diffraction patterns of the prepared MABS have been shown in Figure S1. After 3 d of soaking period in SBF, a broad diffraction peak appeared at 2θ = 32°, which is the characteristic peak of HA, indicating the conversion of BG into HA with low crystallinity. After 14 d soaking period in SBF, a higher and sharper diffraction peak emerged at 2θ = 32°, demonstrating that BG had transformed into HA with higher crystallinity. The intensities of the diffraction peaks of the 20%BG@PgP (20BG) scaffolds were significantly stronger than those of the other scaffolds at all time points. The 4D‐printed MABS exhibited HA crystallization in the simulated in vivo environment at an early stage (as early as 3 d), indicating strong self‐mineralization capability. These findings suggest that a HA binding layer can form shortly after scaffold implantation, facilitating robust integration between the scaffold and host bone. This not only enhances the stability of the scaffold–bone interface but also promotes stem cell migration, proliferation, and differentiation.

We subsequently utilized a universal mechanical testing machine to measure the compressive strengths and moduli of the scaffolds with 20% and 60% porosity. In the compression tests (Figure 2E), the loading rate was 2 mm/s, five measurements were taken for each sample, and outliers were removed to ensure data reliability. Bar graphs of the compressive strengths and moduli, along with the compressive stress‒strain curves, are shown in Figure 2F. The results indicate that at 60% porosity, the compressive strengths of the 5%BG@PgP (5BG), 10%BG@PgP (10BG), and 20%BG@PgP (20BG) scaffolds were 7.8±0.4, 6.6±0.2, and 6.8±0.3 MPa, respectively. When the porosity was adjusted to 20%, the compressive strengths of the 5BG, 10BG, and 20BG scaffolds were 30.3±4.8, 24.3±2.2, and 25.7±0.6 MPa, respectively. The compressive strengths and moduli of the scaffolds exhibited opposite trends with increasing BG content. Among all the scaffolds with 60% porosity, the 20BG scaffold exhibited the highest compressive modulus of 93.7±10.3 MPa. For the scaffolds with 20% porosity, the 5BG scaffold presented the highest compressive modulus of 706.7±30.3 MPa. In the compression experiments, the scaffolds with 60% porosity demonstrated compressive strengths ranging from 6.4 to 8.2 MPa, confirming that all the scaffolds fabricated in this work met the compressive strength requirements (1–10 MPa) of natural cancellous bone [36]. This enhancement may be attributed to the BGs acting as “rigid bodies” to hinder crack initiation. However, owing to the relatively large particle size of the BG and its potential to agglomerate at higher concentrations, once formed, cracks propagate rapidly within the scaffold. Accordingly, the 20BG scaffolds often exhibited significant fracture and damage under large‐scale compression. In summary, the mechanical strength of the 4D‐printed MABS fully meets the supportive requirements and is comparable to that of human bone tissue.

A scaffold needs to maintain sufficient shape memory functionality after forming to provide self‐adaptability. Therefore, we conducted dynamic thermomechanical performance tests on 4D‐printed adaptive bioactive stents. The storage modulus curve of the sample exhibited a decreasing trend in the low‐temperature region and a plateau in the high‐temperature region, indicating that the material is expected to demonstrate shape memory behavior under thermal stimulation. After the material undergoes crystallization and melting transitions, the storage modulus of the composite decreases by two orders of magnitude. Additionally, we compared the endpoint temperature at which the storage modulus of different materials sharply decreased, which corresponded to the peak temperature on the loss factor curve, denoted as the glass transition temperature (Tg). For composites with varying BG contents, the Tg remains within the range of 60°C–70°C with minimal variation, suggesting that the BG content had little effect on the melting point of the composite when in the range of 5%–20%. The dynamic thermomechanical properties of the composite meet the requirements of 4D printing technology.

To assess the pertinent parameters of 4D‐printed MABS in the microenvironment and establish a correlation between the morphological characteristics of BG particles through a multilayer perceptron model, we utilized energy‐dispersive spectroscopy (EDS), inductively coupled plasma–atomic emission spectroscopy (ICP–AES), and noncontact micro‐testing methodologies to analyze the composite scaffolds. The primary elemental constituents of BG include Sr^2+^, Mg^2+^, Si^4+^, Ca^2+^, Na^+^, and B^2+^. We further characterized the elemental composition of the BG@PgP scaffolds using EDS, as illustrated in Figure 2G. The scanning electron microscopy (SEM)/EDS overlay images indicated the uniform integration of BG particles within the PgP polymer across the selected scale (Figure S2). Additionally, ICP–AES, demonstrated that increasing the BG content (5%, 10%, and 20%) within the scaffolds was positively correlated with the quantity of ions released under simulated physiological conditions over the same duration. Furthermore, in the presence of constant concentrations of ions, ion release was also positively correlated with time, indicating the stable, sustained release of ions, predominantly divalent cations such as Sr^2+^, Mg^2+^, and Ca^2+^, from the scaffolds into the microenvironment during their degradation.

Furthermore, we employed a noncontact micro‐testing technique to monitor the pH level on the scaffold surface during degradation when immersed in PBS, as shown in Figure 2H. The pH of the pure 4D‐printed PgP scaffold gradually decreased over time, which aligns with the findings reported in numerous polymer‐related studies [37, 38]. The hydrolytic degradation of polymer materials results in the release of acidic byproducts, which lowers the pH of the surrounding environment and accelerates degradation through self‐catalysis, potentially leading to adverse tissue responses, such as inflammation and foreign body reactions [39]. In contrast, the pH values of all the groups of 4D‐printed MABS significantly increased over time, indicating the continuous and stable release of ions. Notably, the increase in pH for the scaffolds containing 5% and 10% BG plateaued after 6 h, likely due to the consumption of hydroxide ions (OH‐) during HA formation, a phenomenon that has been corroborated by previous investigations into the crystalline structures of such scaffolds. This observation further implies that ion release from the scaffolds remains stable within this pH range. Importantly, the increase in pH was particularly pronounced with the 20% BG scaffold, which may be attributed to its high concentration of BG, increased ion release, and the need for a higher pH to achieve ionic equilibrium.

### 2.2 Interaction Between the Composition of 4D Printed MABS and In Vitro Osteogenic and Angiogenic Differentiation

#### 2.2.1 Viability of BMSCs and HUVECs

**FIGURE 3:**
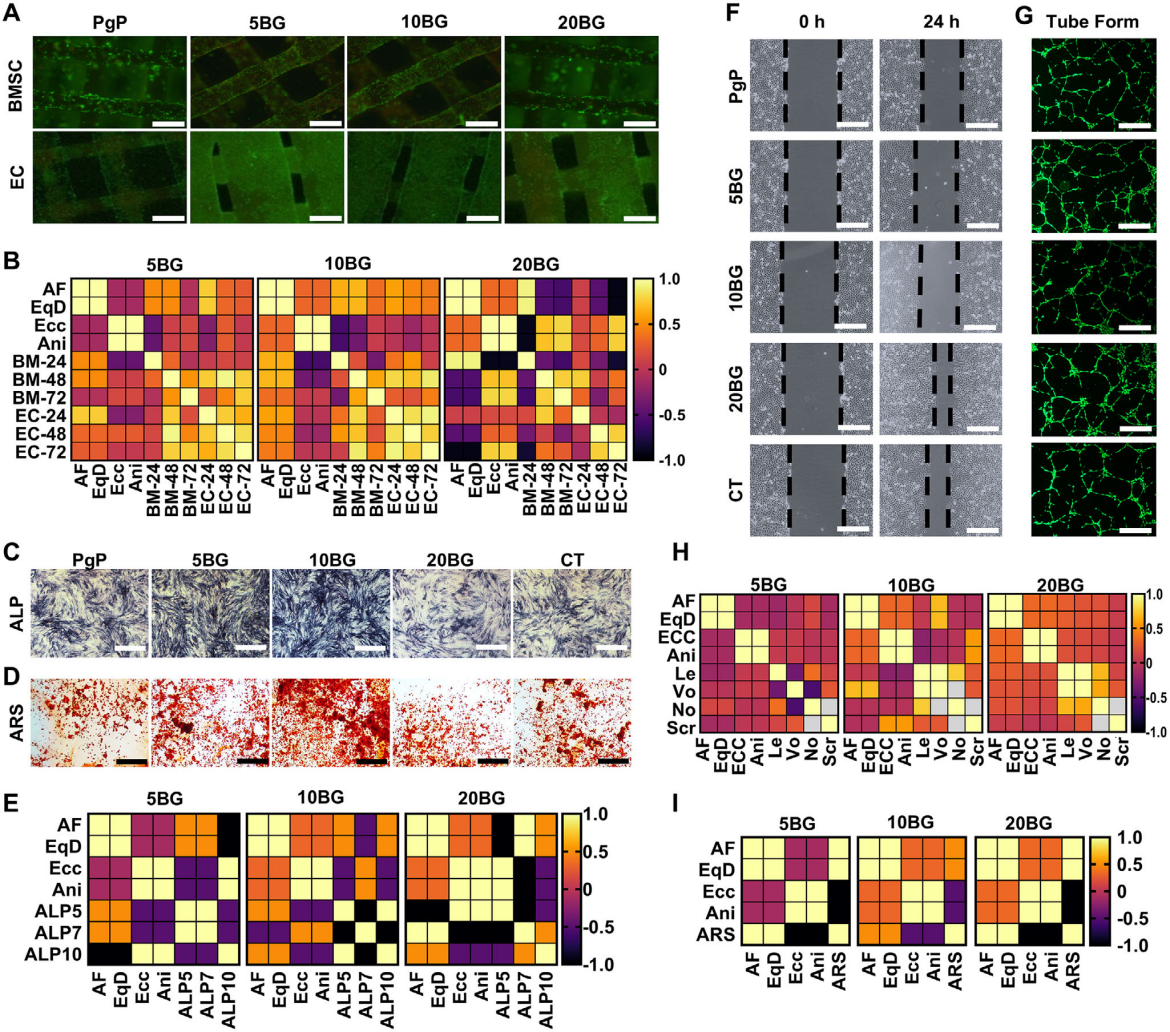
(A) Live/dead staining of BMSCs and HUVECs following 1 week of incubation period on the scaffolds (scale bar: 100 µm). (B) The Pearson's r correlation analysis of the relationship between the weight parameters of the BG in MABS and the viability of BMSCs and HUVECs. ALP staining after 10 days of induction (C) and Alizarin Red S staining (D) of BMSCs following culture on the surface of extracts of MABS (scale bar, 100 µm), with 10 days of incubation period for ALP staining, and 14 days of incubation period for Alizarin Red S staining accordingly. The Pearson's r correlation analysis of the relationship between the weight parameters of the BG in MABS and the ALP (E) activities of BMSCs, as well as the relationship between the weight parameters of the BG in MABS and ARS (I) activities of BMSCs. Representative images depict migration in the scratch assay after 24 h (F) and vascular formation after 4 h (G) of HUVECs treated with MABS extracts (scale bar, 100 µm). (H) The Pearson's r correlation analysis of the relationship between the weight parameters of the BG in MABS and the angiogenic capability of HUVECs. Data are presented as means ± SD, *n* = 3, Statistical significance was determined using the one‐way ANOVA method with Tukey's multiple comparisons tests, Statistical significance was defined as *p < 0.05, **p < 0.01, ***p < 0.001, and ****p < 0.0001, whereas nsP > 0.05 was deemed not statistically significant.

To investigate how composite materials with varying BG contents affect the biocompatibility of mouse bone marrow‐derived mesenchymal stem cells (BMSCs) and human umbilical vein endothelial cells (HUVECs), we conducted live/dead cell staining (Figure 3A). The live/dead staining results demonstrated that after one week of coculture with BMSCs and HUVECs, nearly all the cells on the scaffolds remained viable (green fluorescence), with only a small number of dead cells (red fluorescence), confirming excellent cytocompatibility of all the scaffolds.

Furthermore, a Cell Counting Kit‐8 (CCK‐8) assay was used to evaluate how the scaffolds influenced BMSC proliferation (Figure S3b). The results demonstrated that the proliferative activity of BMSCs in the 10BG group was 1.37‐, 1.10‐, and 1.11‐fold higher than that of the control group at 24, 48, and 72 h, respectively. On day 1, the relative cell viability of the 5BG and 10BG groups was slightly greater than that of the control group, with the 10BG group showing a significant difference (*p* < 0.05). In contrast, the relative cell viability of the 20BG and PgP groups was slightly lower than that of the control group. This trend became more pronounced over time, suggesting that the 5BG and 10BG scaffolds promoted BMSC proliferation.

Similarly, the CCK‐8 assay was used to assess the effect of scaffold toxicity on HUVECs proliferation. The results aligned with those of the BMSC co‐culture: HUVEC activity was 1.22‐, 1.10‐, and 1.22‐fold higher at the corresponding time points. On day 1, the relative cell viability of the 5BG and 10BG groups was slightly greater than that of the control group, with the 10BG group showing a significant difference (*p* < 0.01). Conversely, the relative cell viability of the 20BG and PgP groups was significantly lower than that of the control group (*p* < 0.01). This trend intensified over time, indicating that the 5BG and 10BG scaffolds promoted HUVEC proliferation.

These results suggest that the BG@PgP scaffolds have minimal inhibition on the proliferation of BMSCs and HUVECs, exhibit favorable cytocompatibility, and enhance cell proliferation. To clarify the correlation between the number of proliferating BMSCs and HUVECs over time, the BG particle parameters, and the BG concentration, Pearson's r correlation analysis was performed on the BG weight parameters and biocompatibilities of composite materials with varying contents, as shown in Figure 3B. The three matrix plots represent the relevant parameters of BG particles with different BG contents. For the groups of 5BG and 10BG, AF and EqD exhibited the same trend in bioactivity, as both promoted the proliferation of both BMSCs and HUVECs after 24 and 48 h of culture. However, at 72 h, the scaffold in group of 5BG inhibited the proliferation of BMSCs and HUVECs. Moreover, the scaffold in group of 20BG promoted the proliferation of both cell types only at 24 h, followed by strong inhibition, which may be related to excessive particle release. Furthermore, the parameters Ecc and Ani were correlated with low promotion or inhibition of proliferation in the 5BG and 10BG groups but were correlated with the strong promotion of cell proliferation in the 20BG group.

#### 2.2.2 MABS Component‐Mediated Osteogenic Differentiation of BMSCs In Vitro

To further investigate the in vitro osteogenesis of the MABS, scaffold samples with 4 mm in diameter and 2 mm in height were cultured with BMSCs, and the ALP assays and alizarin red S (ARS) staining of BMSCs at selected time points were conducted. ALP staining was performed on day 5 to identify early bone regeneration, as ALP is a marker enzyme of early bone regeneration (Figure 3C). The results revealed that the ALP‐stained areas in PgP and 20BG groups were not significantly different from that of the blank control (*p* > 0.05), whereas the 5BG and 10BG groups were significantly larger than those in the other three groups (*p* < 0.01) (Figure S3c,d). The increased ALP activity in the 5 and 10BG groups may be attributed to the moderate release of ions (Figure 2G). Additionally, the reduced ALP activity in the 20BG group might be due to excessive ion release during continuous culture (Figure 2G), which aligns with the BMSCs viability results after coculture with the scaffolds (Figure 3A).

Subsequently, ARS staining was conducted in vitro to evaluate the late‐stage mineralization capacity of BMSCs on the MABS (Figure 3D). After 14 d of incubation, more mineralized nodule formation was observed in the 10BG group than in the other four groups. Quantitative analysis revealed that the most mineral deposition occurred in the 10BG group, which exhibited significantly more mineralization than the control group (*p* < 0.001). In contrast, the stained area of the 20BG group was significantly lower than that of the control group (*p* < 0.001). The remaining groups were not significantly different from the control group (*p* > 0.05) (Figure S3e). These results indicate that the 10BG scaffold showed a better promotion on the migration and osteogenic differentiation of BMSCs.

To clarify the correlations among the scaffold‐induced osteogenesis of BMSCs and time, BG particle parameters, and BG concentration, we conducted Pearson's r correlation analysis between the BG weight parameters and osteogenic outcomes with composites of varying contents, as shown in Figure 3E,I. The three matrix plots represent the relevant parameters of the BG particles with different BG contents. The parameters AF and EqD exhibited opposite trends to the parameters Ecc and Ani in terms of ALP activity at all time points across all groups. For the 5BG group, AF and EqD significantly increased ARS staining and ALP activity at 5 and 7 d. However, ALP activity was suppressed at 10 d. Conversely, Ecc and Ani showed the opposite trend by inhibiting ARS staining and ALP activity at 5 and 7 d. Followed by ALP activity was promoted at 10 d, AF and EqD increased ARS staining and ALP activity at 5 and 10 d but decreased ALP activity at 7 d. Ecc and Ani again displayed the opposite trend—inhibiting ARS staining and ALP activity at 5 and 10 d. Whereas ALP was increasing at 7 d. For the 20BG group, AF and EqD significantly promoted ARS staining and ALP activity at 7 and 10 d. While ALP activity was suppressed at 5 d. Finally, Ecc and Ani followed the opposite pattern—inhibiting ARS staining and ALP activity at 7 and 10 d. And ALP activity was increased at 5 d. These findings suggest that the optimal osteogenic concentration was reached at the later release stage for 5BG group, whereas the 10BG group presented an intermediate peak at 7 d. Furthermore, there was initially an excess of ions in the 20BG group, leading to cellular inhibition, after which the concentration of ions gradually declined to the optimal level for osteogenesis after medium replacement. With respect to the inner‐parameter relationships, AF and EqD were negatively correlated with Ecc and Ani in the 5BG group but positively correlated in the 10BG and 20BG groups.

Collectively, these results clarify the concentration‐dependent and temporal‐specific regulatory effects of BG particle parameters on scaffold‐induced BMSCs osteogenesis: the 5BG group achieved optimal osteogenic activity at the late release stage, the 10BG group exhibited an intermediate peak in osteogenic activity at 7 d, and the 20BG group showed a recovery and promotion of osteogenesis after an initial inhibitory phase due to excessive ion release. Additionally, the internal relationships among BG particle parameters were found to be concentration‐dependent: AF and EqD were negatively correlated with Ecc and Ani in the 5BG group, but positively correlated in the 10BG and 20BG groups. This suggests that BG concentration modulates the coupling relationship between particle morphological parameters, which in turn dictates the spatiotemporal pattern of BMSCs osteogenesis induced by composite scaffolds. These findings not only resolve the ambiguities in the original result descriptions but also provide a rigorous mechanistic basis for optimizing BG‐based composite scaffolds for bone tissue engineering applications.

#### 2.2.3 MABS Component‐Mediated Angiogenic Differentiation of HUVECs In Vitro

Following the exploration of MABS components’ osteogenic potential in regulating BMSCs osteogenic differentiation, further investigation into their angiogenic effects is imperative. Osteogenesis and angiogenesis are mutually dependent in bone regeneration: successful bone repair requires not only new bone formation but also a functional vascular network to supply oxygen, nutrients, and growth factors. Insufficient angiogenesis in tissue‐engineered scaffolds often impairs osteogenic efficiency and leads to repair failure. Thus, based on previous osteogenic findings, we focused on MABS Component‐Mediated Angiogenic Differentiation of HUVECs In Vitro, to clarify MABS's role in endothelial angiogenesis and lay a comprehensive foundation for MABS‐based scaffolds with both osteogenic and angiogenic capacities. We investigated the proangiogenic effects of the composite scaffolds through scratch assays and in vitro vascular formation tests. HUVECs in the 5BG and 10BG groups exhibited significant wound healing via migration within 24 h. Semiquantitative analysis revealed that the wound healing effect in the 10BG group was significantly greater than that in the other groups, whereas no significant difference in cell migration was detected between the PgP and control groups (Figure 3F). We employed an in vitro vascular formation assay to evaluate the angiogenic capacity of HUVECs following culture under different conditions (Figure 3G). HUVECs in all BG‐containing groups formed distinct tubular structures, whereas the cells in the PgP and control groups generated only numerous vascular branch structures with discontinuous tube walls. Analysis of the microvessel length, microvessel volume, vascular loop count, and scratch healing rate revealed the superior performance of groups of 5BG and 10BG, with the10BG group showing the best results.

To elucidate the correlation between the proangiogenic effects of the scaffolds on HUVECs and the parameters/concentrations of BG particles, we conducted Pearson's r correlation analysis between the BG weight parameters and angiogenic outcomes for scaffolds of various compositions, as shown in Figure 3H. The three matrix plots represent correlation analyses between BG particle‐related parameters and angiogenesis‐related parameters with scaffolds containing different amounts of BG. In the 5BG and 20BG groups, the four parameters—AF, EqD, Ecc, and Ani—demonstrated weak or slight inhibitory effects on angiogenesis on the basis of metrics such as tubule length, volume, junction number, and cell migration (scratch assay). Conversely, in the 10BG group, AF and EqD promoted an increase in vascular volume, whereas Ecc and Ani promoted cell migration (scratch assay). With respect to the relationships among parameters across groups, AF and EqD presented negative correlations with Ecc and Ani in the 5BG group, whereas positive correlations were observed among these parameters in the groups of 10 and 20BG.

### 2.3 MABS Component‐Mediated Osteogenic and Angiogenic Differentiation of Cells

**FIGURE 4:**
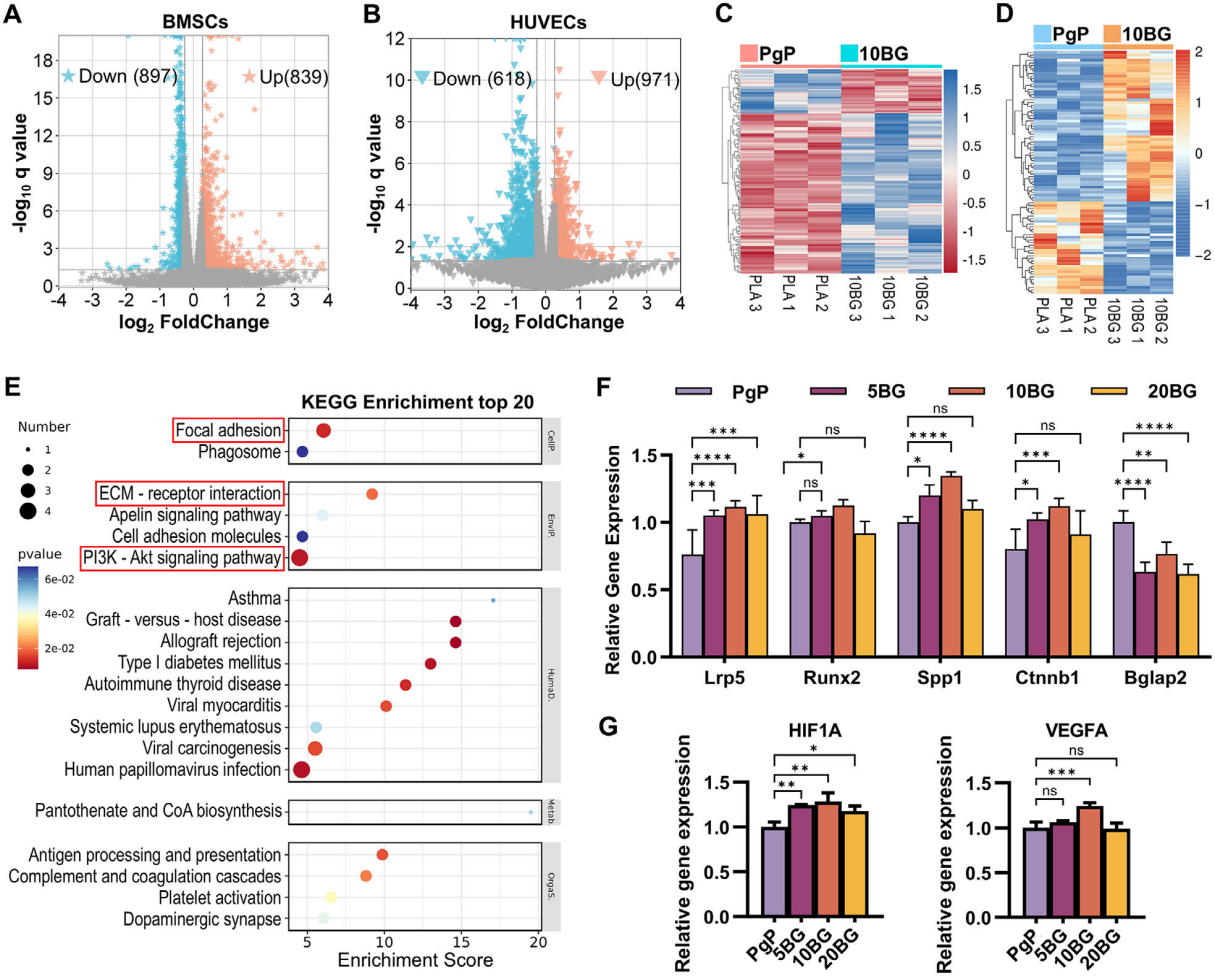
(A) Volcano plot showing DEGs of BMSCs with a culture time of 7 days between the 10BG group and the PgP group (*n* = 3) (*q* value <0.05, |log_2_FC|>0.263). (B) Volcano plot showing DEGs of HUVECs with a culture time of 7 days between the 10BG group and the PgP group (*n* = 3) (*q* value <0.05, |log_2_FC|>0.263). (C) Heatmap of DEGs of HUVECs with a culture time of 7 days between the 10BG group and the PgP group (*n* = 3). (D) Heatmap of DEGs of BMSCs with a culture time of 7 days between the 10BG group and the PgP group (*n* = 3). (E) KEGG enrichment analysis of upregulated signaling pathway changes in BMSCs cultured for 7 days between the 10BG group and the PgP group (*n* = 3). The red boxes indicate pathways highly associated with osteogenic differentiation. (F) Expression of osteogenesis‐related genes (Bglap2, Runx2, Spp1, Ctnnb1, and Lrp5) in BMSCs following 7 days of incubation period with MABS. (G) Angiogenesis‐related genes (HIF1A, VEGF) in HUVECs following 7 days of incubation period with MABS. (Data are presented as means ± SD, *n* = 3, Statistical significance was determined using the one‐way ANOVA method with Tukey's multiple comparisons tests, Statistical significance was defined as *p < 0.05, **p < 0.01, ***p < 0.001, and ****p < 0.0001, whereas ns, *P* > 0.05 was deemed not statistically significant).

To investigate how the scaffold affects the osteogenesis of BMSCs and the angiogenesis of HUVECs, we performed whole‐transcriptome sequencing of BMSCs and HUVECs respectively following culture time of 7 days with the scaffolds designation with PgP and 10BG. The analysis revealed significant differences in gene expression profiles. Specifically, according to the BMSCs sequencing results (Figure 4A), compared with the PgP group, the 10BG scaffold group presented significant upregulation of 839 genes and significant downregulation of 897 genes (*q* value < 0.05). Similarly, based on the sequencing results of HUVECs (Figure 4B), compared with the PgP group, the 10BG group showed significant upregulation of 971 genes and significant downregulation of 618 genes (*q*‐value <0.05). Principal component analysis confirmed the reliability of the transcriptome data, and a heatmap was constructed to visually display the expression patterns of these differentially expressed genes. Additionally, KEGG pathway enrichment analysis of BMSCs showed increased enrichment of osteogenesis‐related pathways (such as PI3K‐Akt signaling pathway, Focal adhesion, ECM‐receptorinteraction, etc., *q* value < 0.05) in the 10BG group (Figure 4E). We examined the expression of key genes associated with the osteogenic differentiation of BMSCs and the angiogenic differentiation of HUVECs that were differentially expressed, including JPH2, Spp1, CCR4, PPARG, FGF22, CACNA2D4, RasGRP1, Ctnnb1, Lrp5, CDH26, Fgf23, Noct, Bmpr1b, Runx2, HIF1A, CDH4, and Vegfc.

To gain deeper insights into the functional implications of these differentially expressed genes (DEGs), we conducted KEGG pathway analysis (*q* < 0.05) on the EDGs in the PgP and BG@PgP groups, focusing on genes related to osteogenesis and angiogenesis. This analysis revealed several potential pathways through which the 4D‐printed BG@PgP scaffold may affect osteogenesis and angiogenesis. These pathways include “Ca^2+^ release (*p* = 0.043),” “voltage‐dependent calcium channels (*p* = 0.034),” the “NOTCH signaling pathway (*p* = 0.021),” the “calcium signaling pathway (*p* = 0.043),” the “Wnt/β‐catenin signaling pathway (*p* = 0.016),” “regulation of autophagy‐mediated inflammation (*p* = 0.002),” “inhibition of the Notch1/Jagged pathway (*p* = 0.011),” which are potentially associated with the osteogenic differentiation promoted by the active components of BG. The whole‐transcriptome sequencing results of the HUVECs indicate that, the degradation products of BG in scaffold upregulated CDH4 gene expression in HUVECs. Research indicates that the connection between CDH4 and β‐catenin can impede the β‐TrCP1‐mediated ubiquitination and degradation of β‐catenin, thus enhancing its nuclear translocation and augmenting the transcription of downstream genes to promote angiogenesis [40]. Furthermore, sequencing the BMSCs revealed a relative augmentation in the expression of peroxisome proliferator‐activated receptor γ (PPARG). Research indicates that PPARG is essential for the synthesis of sclerostin, a newly sanctioned medicinal target for osteoporosis. In osteocyte‐specific PPARG‐deficient animal models (Dmp1CrePparγfl/fl or γOTKO), traits such as elevated bone marrow adipocyte numbers and decreased marrow adiposity were noted, correlating with activated WNT signaling and enhanced bone formation activity by endosteal osteoblasts [41]. The Spp1 gene, which is highly expressed, regulates immune cell activity and inflammatory responses, and plays a vital role in rheumatoid arthritis research. This gene is incorporated into the unmineralized matrix before calcification, where it contributes to ossification and may have roles in cardiovascular disorders [42].

Therefore, qPCR assay was conducted to validate the expression of osteogenic‐related genes in BMSCs and angiogenic‐related genes in HUVECs following incubation with scaffolds for 7 days. Figure 4F illustrates our analysis of the transcriptional level of osteogenic differentiation in BMSCs. Following the direct contact coculture model, the expression levels of the Runx2, Spp1, Ctnnb1, and Lrp5 genes in the 5BG and 10BG groups were markedly elevated compared to the PgP group (*p* < 0.01). No significant change was noted between the 20BG and PgP groups (*p* > 0.05). Meanwhile, the angiogenic differentiation of HUVECs was analyzed at the transcriptional level. The expression levels of HIF1A, VEGF, and other genes in the 10BG group were markedly elevated compared to the other groups (*p* < 0.01) (Figure 4G). After co‐culture for 7 days and 2 days, respectively, BMSCs and HUVECs were lysed for Western blotting detection. BG promoted osteogenic differentiation, with the 10BG group exhibiting significant osteopontin (OPN)‐promoting activity (Figure S14b). An increased proportion of active dephosphorylated Ctnnb1 indicates activation of the Wnt/β‐catenin signaling pathway, initiating downstream osteogenic gene expression (Figure S14a). Similarly, under 2 days of co‐culture, 10BG demonstrated superior pro‐angiogenic activity (Figure S14c).

The results validated that, with the appropriate incorporation of BG, the scaffold enhanced the expression of Wnt/β‑catenin‑associated genes Ctnnb1 and LRP5 in cells, suggesting that the BG@PgP composite may facilitate bone tissue healing by activating the Wnt/β‑catenin pathway. However, the pro‑angiogenic effects were not uniform across all BG concentrations: the 5BG and 10BG groups consistently promoted HUVEC tube formation, migration, and angiogenic gene expression, whereas the 20BG group showed only transient pro‑angiogenic activity at early time points (24 h) followed by significant inhibition at 48–72 h (Figure 3G,H and Figure S3b). This concentration‑dependent biphasic response likely reflects excessive ion release (e.g., Sr^2^^+^, Ca^2^^+^) and pH elevation (Figure 2G,H), which can compromise endothelial cell function at high doses [43, 44, 45].

### 2.4 The Mediating Role of the 4D‐Printed MABS in In Vivo Osteogenesis and Vascular Reconstruction

Subsequent to the in vitro evaluation of the osteogenesis and angiogenesis of MABS, rat calvarial defect models were created to investigate the in vivo efficacy of the 4D‐printed MABS in mediating osteogenesis and vascular reconstruction.

#### 2.4.1 Osteogenesis of 4D‐Printed MABS in Rat Calvarial Defect Models

**FIGURE 5:**
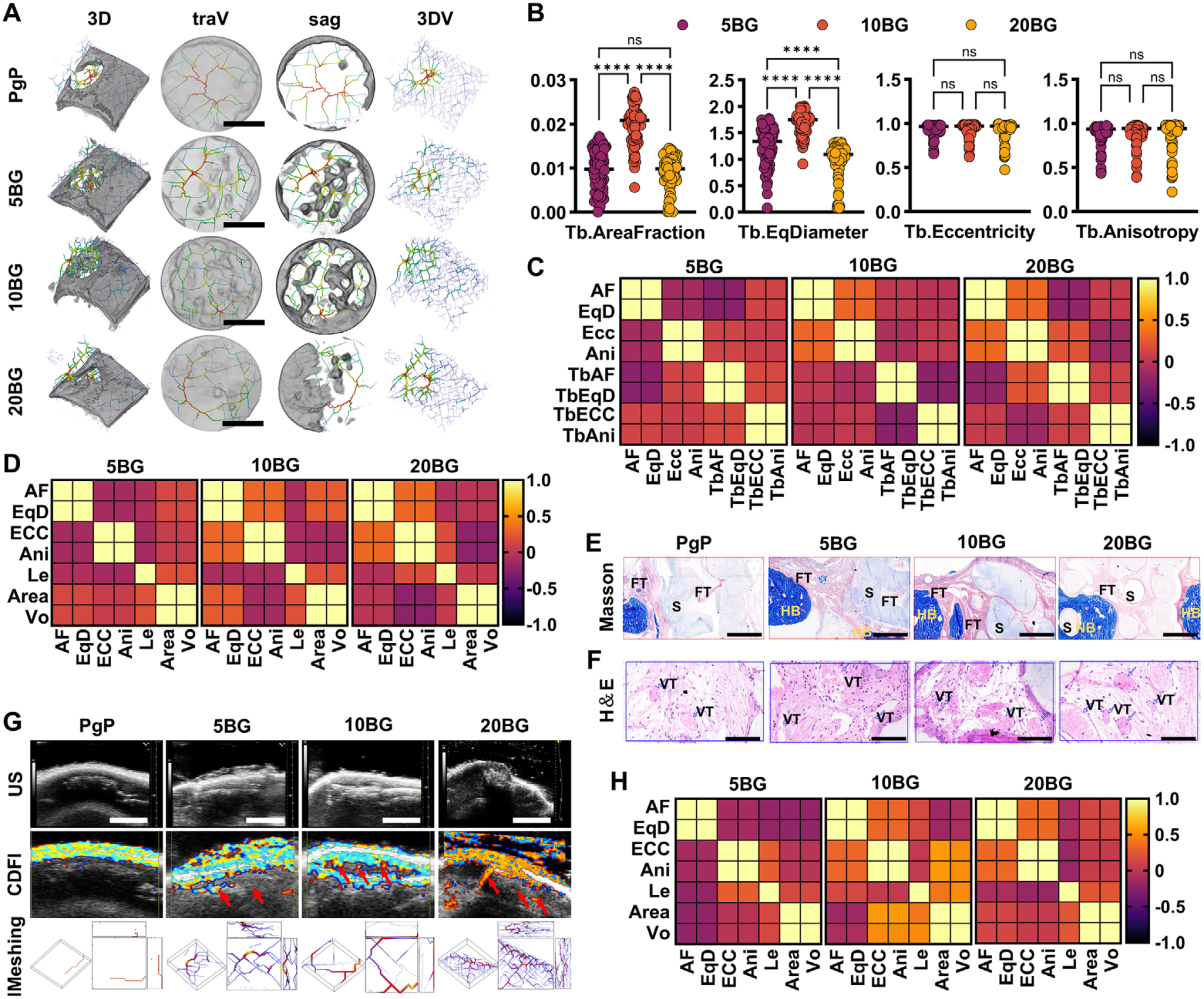
(A) 3D reconstruction of micro‐CT analysis illustrating the new bone formation and blood vessels in growth in the rat calvarial defect model following the implantation of MABS for 4 weeks. The pseudocolor temperature increases as the relative diameter of the neovascularization thickens (Scale bar: 2 mm, traV: translucent vessels, Sag: sagittal, 3DV: Three‐dimensional vascular reconstruction). (B) The simulating results of four morphological parameters (AF, EqD, Ecc, Ani) relating to the new trabecular bone formation in the rat calvarial defect model following the implantation of MABS for 4 weeks. (C) The Pearson's r correlation analysis of the relationship between the morphological parameters of the BG in MSA and the macroscopic morphological parameters of new bone formation in the rat calvarial defect model following MABS implantation. (D) The Pearson's r correlation analysis between the morphological parameters of the BG in MABS and the micro‐CT‐reconstructed neovascularization‐related parameters in the rat calvarial defect model. (E) Masson's trichrome staining evaluation of the bone formation following the implantation of MABS for 4 weeks in rat calvarial defect model (scale bar: 100 µm); Abbreviations: S, Scaffold; NB, new bone; HB, host bone; FT, fibrous tissue. (F) Hematoxylin and eosin (H&E) staining evaluation of the bone formation following the implantation of MABS for 4 weeks in the rat calvarial defect model; Abbreviations: Vascular tissue, VT (scale bar: 100 µm). (G) High‐resolution ultrasound/photoacoustic imaging revealing the subcutaneously vascular recruitment following the implantation of MABS for 4 weeks in the rat calvarial defect model (Scale bar: 2 mm). (H) The Pearson's r correlation analysis between the morphological parameters of the BG in MABS and ultrasound‐derived neovascularization‐related parameters in the rat calvarial defect model. (Data are presented as means ± SD, *n* = 3, Statistical significance was determined using the one‐way ANOVA method with Tukey's multiple comparisons tests, Statistical significance was defined as *p < 0.05, **p < 0.01, ***p < 0.001, and ****p < 0.0001, whereas ^ns^ *P* > 0.05 was deemed not statistically significant).

The quantitative analysis and 3D reconstruction of the bone repair regions were assayed utilizing micro‐CT in each group four weeks post‐MABS implantation, as illustrated in Figure 5A. Representative images from each group featured the bone surface. The collective images of the BG@PgP group demonstrated the existence of newly developed bone bridges. The reconstructed intensity of the skull and scaffold, derived from micro‐CT values in the PgP and BG@PgP groups, illustrated the bone defect healing results throughout the four groups (Figure S4b). In the cross‐sectional micro‐CT scans of the BG@PgP groups, pronounced early‐stage new bone ingrowth was distinctly detected at the interface between the scaffold and the bone. The predesigned high‐porosity sections of the scaffold, resembling cancellous bone, promoted more new bone ingrowth as observed in the coronal and sagittal views. In comparison to the PgP group, the BG@PgP groups demonstrated enhanced osseointegration, establishing continuous bone bridges on the surface and significant new bone within the scaffold. This trend escalated with the augmentation of BG content in the scaffold. The statistical analysis of the CT results indicated a tendency that aligns with the 3D reconstructed pictures, as demonstrated in Figure 5B. The osteogenic indices Tb.N, Tb.Th, and Tb.Sp in the PgP and BG@PgP groups corresponded with the trends depicted in the figures.

Hematoxylin and eosin (H&E) and Masson staining were utilized to assess the influence of the fabricated scaffolds on bone regeneration. Figure 5E illustrates that Masson staining mirrored the results of H&E staining, with BG‐containing scaffold groups displaying mature bone tissue dyed blue, akin to the stained cranial bone, a phenomenon absent in the pure PgP scaffolds. In comparison to the PgP scaffolds, the BG‐incorporated scaffolds exhibited notable osteogenic characteristics. Masson staining of the fibrous tissue, which exhibited a red hue, indicated the presence of fibrous tissue on both the surface of the normal cranial bone and within the BG‐containing scaffolds. Furthermore, BG‐incorporated scaffolds exhibited a higher density of red staining compared to the pure PgP scaffolds. The stained tissue is presumably a fibrous callus capable of differentiating into osteoblasts and has accumulated significantly in BG‐containing scaffolds with partial mineralization, suggesting that the BG@PgP composite scaffolds exhibit remarkable efficacy in facilitating osteoblast migration, proliferation, and differentiation, aligning with the in vitro experimental findings.

Likewise, in the H&E‐stained hard tissue slices from each group (Figure 5F), no histological signs of thermal injury—such as coagulation necrosis, tissue charring, or an atypical inflammatory response—were observed in the peri‐implant region at 4 weeks. Furthermore, the 60°C reset procedure did not appear to impede the host tissue's regenerative capacity, as new bone growth was distinctly seen in all BG@PgP scaffold groups, and the 60°C reset temperature did not induce irreversible damage or inflammatory infiltration in the surrounding tissues of the scaffold. The discolored regions aligned with the initial cranial bone tissue model, exhibiting extensive lighter staining indicative of fibrous tissue populated by many cell nuclei within the scaffold pores. The amount of this tissue augmented with rising BG content. Conversely, the implantation site in the PgP control group was only partially occupied by dispersed fibrous tissue, with no notable new bone growth detected. Nevertheless, undamaged scaffold structures were observable in both the control and experimental groups, suggesting that extended implantation and enhanced tissue growth did not result in significant scaffold breakdown.

To elucidate the relationships between the BG particle parameters and concentration in the scaffold‐facilitated osteogenic differentiation of BMSCs, we performed Pearson's r correlation analysis of the BG parameters and trabecular bone parameters within composites of diverse compositions, as illustrated in Figure 5C. The three matrix figures provide correlation analyses between pertinent BG particle properties and trabecular bone parameters in scaffolds with varying BG concentrations. In the 5BG and 20BG groups, the parameters AF and EqD of BG exhibited relatively mild negative correlations with the trabecular AF and EqD parameters, however, in the 10BG group, the AF and EqD parameters had no significant impact on the trabecular AF and EqD parameters. The Ecc and Ani parameters primarily exhibited negative correlations with multiple parameters across all groups, with the exception of positive correlations with the trabecular AF and EqD parameters, particularly in the 20BG group.

#### 2.4.2 Angiogenesis of 4D‐Printed MABS in Rat Calvarial Defect Models

We utilized a high‐resolution small animal ultrasound/photoacoustic imaging system (model Vevo‐LAZR‐X) to assess vascular regeneration in mice with cranial defects four weeks post‐scaffold implantation, as well as the vascular distribution within the scaffold and adjacent soft tissues, employing two‐dimensional ultrasound imaging, color Doppler flow imaging (CDFI), and pulsed‐wave Doppler (PW) techniques, as illustrated in Figure 5G. Due to the diminutive size of nascent arteries, 2D imaging was insufficient for detection, but Color Doppler Flow Imaging (CDFI) effectively identified neovascularization within the scaffold and surrounding soft tissues. To mitigate influence from ultrasonic artifacts, PW was employed to evaluate blood flow velocity in areas delineated by CDFI, thereby validating that these vessels had traits of functional neovascularization. The findings demonstrated observable blood flow signals in all mouse models implanted with BG@PgP scaffolds, signifying functional neovascularization (Figure S5). Three‐dimensional reconstruction indicated inadequate new bone development and the lack of discrete vascular networks in the pure PgP scaffold group. The porous architecture of the BG@PgP scaffolds facilitated neovascularization in the bone defect region and adjacent soft tissues, with a positive correlation between vascular density and BG concentrations. These findings indicate that BG@PgP scaffolds promote the development of functional neovasculature, therefore establishing conducive conditions for osteogenesis. Nevertheless, the strength of this angiogenic advantage varied across BG concentrations and measurement modalities. As shown in the correlation analyses (Figure 5D,H), the 10BG group exhibited consistent positive correlations between BG particle parameters (AF, EqD) and neovascularization metrics (vessel volume, length, and loop count). In contrast, the 5BG and 20BG groups showed mild to moderate negative correlations for several vascular parameters, suggesting that their pro‑angiogenic effects are more modest and context‑dependent. The 20BG group, despite showing higher vascular density by histology (Figure 5F), displayed parameter‑specific inhibitory correlations (e.g., Ecc and Ani with vessel volume), indicating that the angiogenic benefit is not uniform across all structural features of the vasculature. Such model‑dependent discrepancies highlight the need for multi‑parametric evaluation and reinforce the conclusion that an intermediate BG concentration (10%) provides the most robust and consistent angiogenic stimulation.

Additionally, we measured vascular development in the bone defect region of each group using reconstructed micro‐CT images, as illustrated in Figure 5A and Figure S4c,d. The representative photos for each group encompass the bone surface and vascular network. The aggregated photos of the BG@PgP group revealed the existence of freshly developed bone bridges encircled by extensive, interconnected vascular networks. Furthermore, the scaffold's porosity architecture incorporated newly formed blood arteries permeating the entire bone defect region. Conversely, the other groups had thinner vascular networks accompanied by inadequate new bone growth. The BG@PgP group exhibited blood vessels characterized by thick walls and extensively branched lumens, while the vessels in the PgP group were markedly thinner and narrower. Only membranous structures with blood arteries were noted in the control group. The enlarged images demonstrated vascular connections between the membrane and the bone margins. In the rebuilt vascular picture, the BG@PgP group exhibited a dense distribution of blood vessels that nearly mirrored the scaffold contour pattern. The vascular representation within the scaffold's porous domains established a network resembling the porous structure. In contrast, the vessels in the alternative groups were limited and scattered. A unique vascular network was noted in the control group. The soft tissue reconstruction images further confirmed the relationship between tissue density and vascular thickness. The BG@PgP group displayed significant soft tissue with a substantial blood supply surrounding the bone defect region. The soft tissue layer was noted to cover the newly created bone, supported by the BG@PgP scaffold positioned beneath the soft tissue. The statistical examination of the CT results indicated tendencies that aligned with the 3D reconstructed images. At the identical radius, the BG@PgP group demonstrated a heightened propensity for consistency regarding vascular volume and length. Conversely, vessels in the alternative groups exhibited reduced density and a propensity for dispersion. Moreover, the BG@PgP group exhibited enhanced vascular quality and quantity, characterized by an elevated vessel count, volume, and length. The ratio of terminal to branching vessels revealed that the BG@PgP group demonstrated a superior capacity for enhancing vascular system growth. Figure 5F illustrates H&E‐stained tissue samples, revealing multiple vascular systems within the fibrous tissue of the scaffold. These vessels displayed morphological resemblances to the blood vessels associated with the periosteum on the surface of the cranial bone. We observed a significant quantity of intact, anucleated cells within these capillaries, aligning with the morphology of erythrocytes in blood vessels. This discovery further substantiates the prior ultrasound and CT findings, demonstrating vascularization within the scaffold. In comparison to the PgP scaffold group, the BG‐containing scaffold groups exhibited markedly more vascular tissue creation, with vascular tissue density positively associated with the BG content in the scaffold.

To elucidate the relationships between the scaffold's capacity to enhance in vivo and ectopic vascularization and the parameters and concentrations of BG particles, we performed Pearson's r correlation analysis on the BG weight parameters and vascularization outcomes in composites with differing contents, as illustrated in Figure 5D,H. The results revealed that the 10BG group consistently exhibited positive correlations between BG particle parameters (AF and EqD) and orthotopic neovascularization metrics (area, volume, and total vessel length), as well as between Ecc/Ani and increased neovascularization area and volume. In contrast, the 5BG and 20BG groups showed predominantly weak or inhibitory correlations across most vascular parameters. Notably, the 20BG group—despite displaying higher vessel density in histological sections (Figure 5F)—exhibited negative correlations between particle anisotropy (Ani) and vessel volume, suggesting that while more vessels formed, their three‑dimensional organization and functional quality may be suboptimal. These findings indicate that the angiogenic advantage of BG‑containing scaffolds is both concentration‑ and parameter‑dependent, with the 10BG scaffold providing the most robust and consistently positive effect across all quantitative modalities.

### 2.5 Validation of the Biological Function and Effectiveness of the Deep Learning Derived 4D‐Printed MABS

**FIGURE 6:**
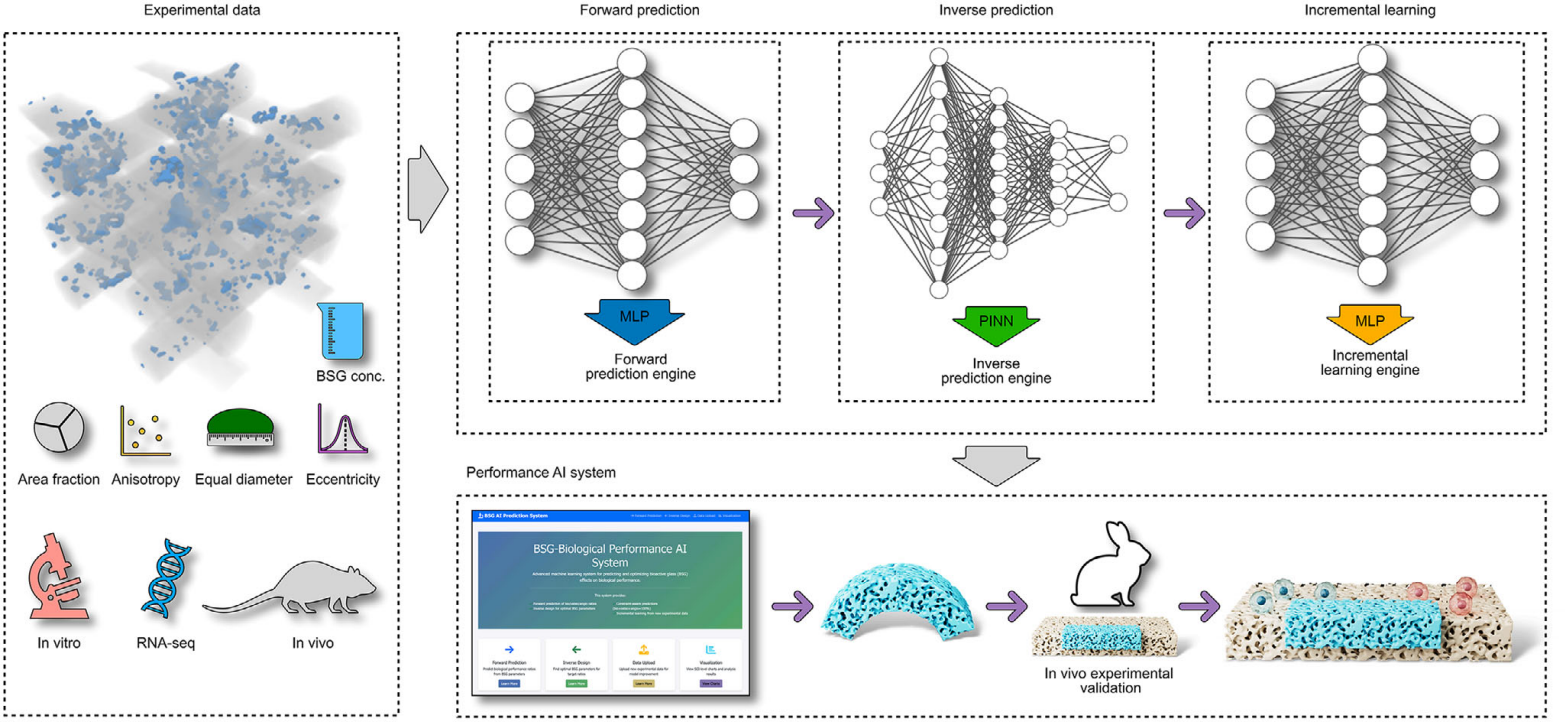
Schematic illustration of the DLN‐driven design paradigm for bone scaffolds. This paradigm integrates three core modified driving engines, datasets (comprising micro‐particle morphological parameters and in vitro/in vivo biological performance parameters), and in vivo validation conducted on a rabbit model. The workflow proceeds as follows: (1) acquisition of primary experimental datasets and incremental datasets; (2) input of qualified datasets into the modified driving engines for feature recognition and deep learning; (3) visualization of analytical results and data at the terminal interface. 4D scaffolds were printed according to the design and in vivo validation was performed in a rabbit bone defect model. The built‐in database supports dual deployment modes (web‐based online access and local offline download) and enables direct output of optimized scaffold parameter datasets for subsequent in vitro and in vivo experimental validation.

Here, a modified multilayer perceptron engine was utilized to simulate and analyze the characterization, in vitro biocompatibility, osteogenic and angiogenic activity, gene pathways, and in vivo osteogenic and angiogenic activity data of the 4D‐printed MABS acquired in the preliminary phase. Furthermore, a backpropagation‐enabled deep learning model was developed to examine the impact of the BG topological characteristics on the overall performance of the scaffolds, including biocompatibility, osteogenic capabilities, and angiogenic properties (Figure 6).

### 2.6 Model Performance Metrics

The MLP model exhibited exceptional predictive performance across all biological indicators (Table 1). For biocompatibility, the model achieved an *R* ^2^ score of 0.983, mean absolute error (MAE) of 0.121, root mean square error (RMSE) of 0.141, and mean absolute percentage error (MAPE) of 4.80%. Osteogenesis prediction yielded an *R* ^2^ of 0.955, MAE of 0.116, RMSE of 0.146, and MAPE of 6.70%. Angiogenesis prediction showed an *R* ^2^ of 0.893, MAE of 0.125, RMSE of 0.171, and MAPE of 9.69%. The overall model performance was characterized by an *R* ^2^ of 0.944, MAE of 0.121, and MAPE of 7.06% (Figure S6).

**TABLE 1:** Model performance metrics for biological performance prediction.

Indicator	R^2^ Score	MAE	RMSE	MAPE [%]	Symmetric MAPE [%]
Biocompatibility	0.983	0.121	0.141	4.80	4.78
Osteogenesis	0.955	0.116	0.146	6.70	6.71
Angiogenesis	0.893	0.125	0.171	9.69	9.60
Overall	0.944	0.121	0.153	7.06	7.03

### 2.7 Constraint Validation Results

Strict constraint validation (sum of bio + osteo + angio = 100% ± 0.1%) was conducted across four scenarios: forward prediction (100 samples), reverse optimization (50 samples), virtual experiments (50 samples), and incremental learning (20 samples). The constraint satisfaction rate was 100% for all scenarios, with an average constraint error of <1e‐06 and a maximum constraint error of <1e‐05 (Table 2). Automatic mathematical correction ensured that all predictions adhered to the biological feasibility constraint (Figures S7b, S8b, and S9e).

**TABLE 2:** Constraint validation results.

Validation Scenario	Sample Size	Constraint Satisfaction Rate	Average Error	Maximum Error	Result
Forward Prediction	100	100%	1e‐06	8e‐06	Pass
Reverse Optimization	50	100%	2e‐06	1e‐05	Pass
Virtual Experiments	50	100%	1.5e‐06	9e‐06	Pass
Incremental Learning	20	100%	2e‐06	8e‐06	Pass

### 2.8 Reverse Optimization Results

Two target biological performance combinations were successfully optimized (Table 3). For the “high biocompatibility” target (bio = 3.00, osteo = 2.00, angio = 1.50), the optimal BG parameters were determined as BG = 11.62, AF = 0.000079, Ani = 0.560, Ecc = 0.732, and EqD = 5.71. The predicted performance values were bio = 2.69, osteo = 1.87, angio = 1.38, with a total optimization error of 0.5578%. For the “high angiogenesis” target (bio = 2.00, osteo = 2.00, angio = 2.50), the optimal BG parameters were nearly identical, with a total optimization error of 1.9399% (Figure S8).

**TABLE 3:** Reverse optimization results for target performance combinations.

Target combination	Optimal BG Parameters [BG, AF, Ani, Ecc, EqD]	Predicted performance [bio, osteo, angio]	Total error [%]	Iterations
High Biocompatibility	(11.62, 0.000079, 0.560, 0.732, 5.71)	(2.69, 1.87, 1.38)	0.5578	2
High Angiogenesis	(11.62, 0.000080, 0.560, 0.732, 5.71)	(2.69, 1.87, 1.38)	1.9399	1

### 2.9 Virtual Experiment and Statistical Validation

Virtual experiment validation with 50 samples demonstrated consistent model reliability (Table 4). The average MAE values were 0.134 ± 0.077 for biocompatibility, 0.121 ± 0.095 for osteogenesis, and 0.128 ± 0.117 for angiogenesis, with an overall MAE of 0.383 ± 0.188. Paired *t*‐tests revealed no significant differences between predicted and true values (biocompatibility: *t* = ‐0.450, *p* = 0.655; osteogenesis: *t* = 1.352, *p* = 0.183; angiogenesis: *t* = −0.792, *p* = 0.432), confirming statistical robustness. The average model confidence was 0.793, with aberration sample rate of 4% (Figure S9).

**TABLE 4:** Virtual experiment validation results.

Indicator	MAE ± SD	Maximum Error	p‐value (t‐test)	Confidence (Mean ± SD)
Biocompatibility	0.134 ± 0.077	0.431	0.655	0.78 ± 0.12
Osteogenesis	0.121 ± 0.095	0.529	0.183	0.79 ± 0.11
Angiogenesis	0.128 ± 0.117	0.630	0.432	0.81 ± 0.10
Overall	0.383 ± 0.188	1.230	—	0.79 ± 0.11

### 2.10 Incremental Learning and Web Application Performance

Incremental learning improved model performance by 3.6% after incorporating new data, with no catastrophic forgetting observed. The web application achieved a response time of <2 s per prediction and supported concurrent access by 100+ users. Real‐time constraint validation and user‐friendly interfaces (forward/reverse prediction, data upload, result export) ensured practical applicability for both research and industrial use.

#### 2.10.1 Screening of Decoding Models for Evaluating the Biological Functions of 4D‐Printed MABS

To dynamically assess the nonlinear correlations among the topological parameters (AF, EqD, Ecc, and Ani) of the BG particles and scaffold performance, and to attain an exact equilibrium among mechanical strength, ion release kinetics, and biological responses, three machine learning regression models were utilized for screening: MLP, random forest, and XGBoost, all of which exhibited commendable interpretability. Nonetheless, their performance evaluation measures displayed specific discrepancies. The mean squared error (MSE) represents the average of the squared discrepancies between projected and actual values, giving more significance to larger errors. The MLP model attained the minimal MSE among the three models, registering a value of 0.02006%. The RMSE, which is the square root of the MSE, yields error values in the same units as the original data. The square root transformation exhibits reduced sensitivity to outliers. The MLP model exhibited superior performance, achieving an RMSE of 0.14166%. The MAE computes the average of the absolute discrepancies between anticipated and actual values, attributing equal significance to all errors. The MLP model exhibited superior performance, achieving an MAE of 0.10514%. The MAPE calculates the average of the absolute percentage discrepancies between expected and actual values, facilitating comprehension of the size of prediction mistakes in relation to actual values. Among the MLP, random forest, and XGBoost models, the MLP model had the lowest prediction errors, with projected values closely aligning with the true values, resulting in a MAPE of 6.14093%. The symmetric mean absolute percentage error (SMAPE), a symmetric variant of the MAPE, considers relative discrepancies between actual and anticipated values, yielding a more equitable error metric. The MLP model once more exhibited superior performance, achieving a SMAPE of 6.18385%, signifying that the model's prediction errors were around 6% of the actual values. To quantify the stability of model performance, we report the mean ± standard deviation of key metrics from five‐fold cross‐validation in Table S1, including MSE, R^2^, RMSE, and MAE. A detailed summary table of cross‐validation results has been included in the supplementary materials (Table S1).

The results indicate that the model exhibits minimal and consistent prediction errors in evaluating biocompatibility, osteogenic effects, and angiogenic effects, hence offering reasonably precise predictions for practical applications. In comparison to the MLP model, the random forest and XGBoost models acquired increasingly diminished accuracies. The R‐squared number denotes the ratio of variability accounted for by the model in relation to the overall variability, serving as an indicator of the model's fit to the data. The MLP model's R‐squared coefficient of 0.945, approaching 1, indicates superior explanatory power and optimal fitting performance, implying that this model adeptly captures the intricate relationships between input features and target variables to predict biocompatibility, osteogenic effects, and angiogenic effects, with significant explanatory capacity for the variability of the target variables. These metrics are complementary, providing a thorough assessment of the model's efficacy in predicting biocompatibility, osteogenic effects, and angiogenic effects from several perspectives. Despite the coefficients of determination for the random forest and XGBoost models being inferior to those of the MLP model, both surpass 0.9, indicating dependable data fitting efficacy. According to the aforementioned results (Figure S6), we cataloged the alterations in the weight parameters of the neurons in the MLP during its operation, and established the correlation between the input and output neuron characteristics. Based on the aforementioned results (Figure S6e), all three deep learning models demonstrate little error, substantial stability, resilience to predictive bias, and strong explanatory capacity. The MLP model surpasses the other models across all evaluated criteria, indicating superior reliability.

#### 2.10.2 Validation of the Effectiveness of the Multilayer Perceptron Model for Evaluating the Biological Functionalities of Composite Materials

On the basis of the aforementioned studies regarding the osteogenic and angiogenic characteristics of BG@PgP scaffolds in rat models, we refined and iterated a 4D‐printed MABS based on AI‐derived parameters, subsequently conducting in vivo validation of its bone repair efficacy. Therefore, a rabbit tibial defect model was created to assess the flexibility of the 4D‐printed MABS, along with its mechanical support, biocompatibility, and capability for bone regeneration in extensive bone defects. The scaffold could be preprogrammed for implantation into the tibial defect, where it restored its shape upon stimulation with sterile saline at 60°C and entirely occupied the defect region. It adaptively adjusted to both the internal and external conditions of the defect, providing robust mechanical support without requiring supplementary plate fixing. The surgery entailed a minor incision, and postoperative recovery advanced positively (Figure S10).

**FIGURE 7:**
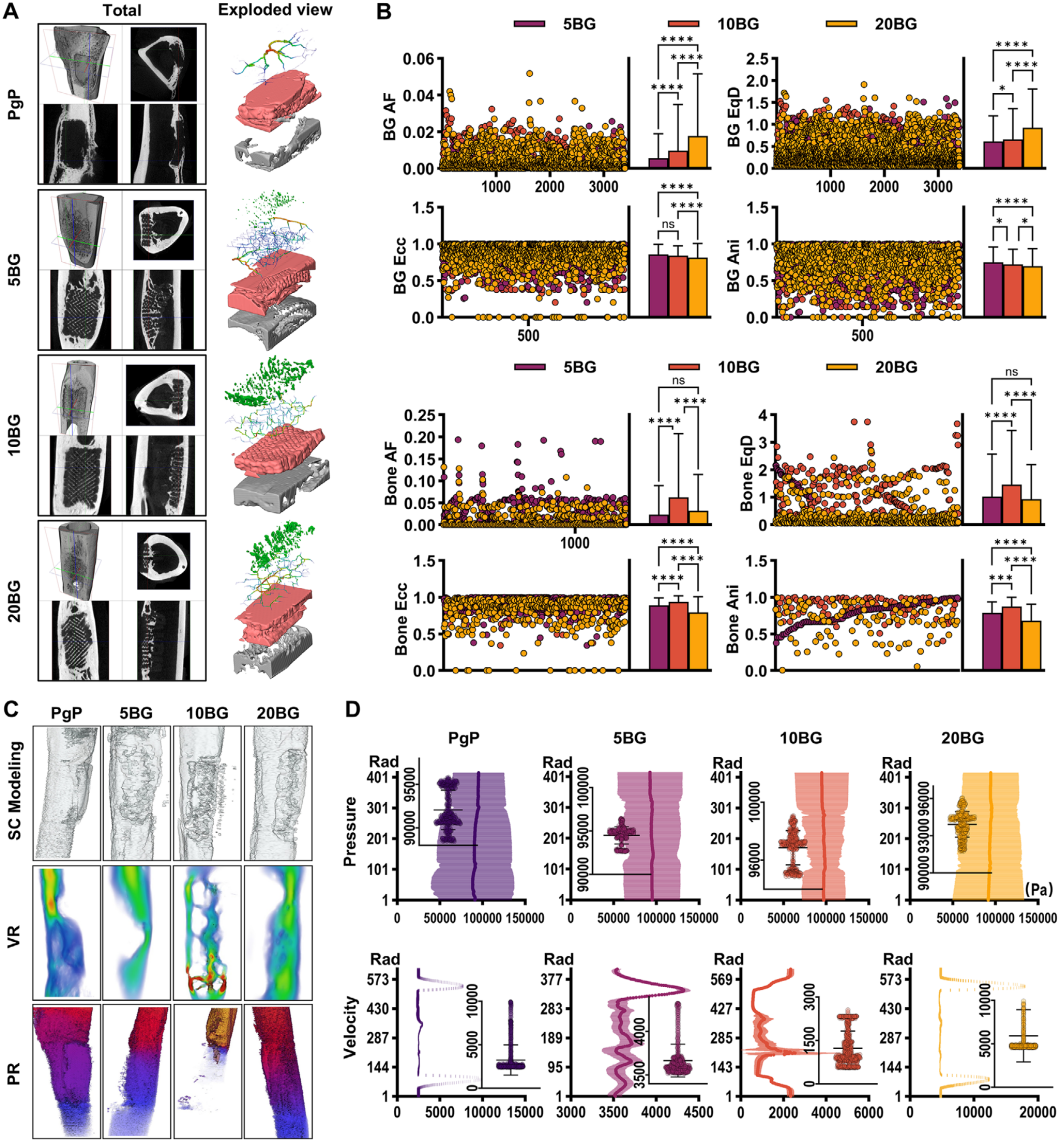
(A) Sagittal and 3D reconstruction of micro‐CT of new bone formation in the rabbit tibial defect model following the implantation of MABS for 8 weeks, and the exploded view illustrating 3D reconstruction of the multi‐component of new formed bone, from bottom to top including bone tissue (gray area), combination of soft tissue and remain MABS (pink area), reconstructed blood vessels (network area), and BG (green area). (B) Specific numerical distribution and statistical analysis of morphological microparameters relating to the distribution of BG within MABS following implantation period of 8 weeks, including Area Fraction (AF), Eq diameter (EqD), Ec centricity (Ecc), Anisotropy (Ani); and quantitative distribution and statistical evaluation of the four morphological microparameters relating to new bone formation in the rabbit tibial defect model following the implantation of MABS for 8 weeks (Area fraction: 0‐1, percentage; Eccentricity: 0‐1, percentage; Anisotropy: 0‐1, percentage; Equivalent diameter: micrometers). (C) 3D reconstruction and fluid simulation of the medullary cavity illustrating the Biocompatibility of stents in animal models following the implantation of MABS in the rabbit tibial defect, VR: velocity rendering; PR: pressure rendering. (D) Finite element quantitative assessment of pressure and flow velocity variations in the medullary cavity following the implantation of MABS in the rabbit tibial defect. (Data are presented as means ± SD, *n* = 3, statistical significance was determined using the one‐way ANOVA method with Tukey's multiple comparisons tests, Statistical significance was defined as *p < 0.05, **p < 0.01, ***p < 0.001, and ****p < 0.0001, whereas ^ns^P > 0.05 was deemed not statistically significant).

At 8 weeks post‐implantation of the 4D‐printed MABS, micro‐CT was employed to assess the tibial deformities in New Zealand rabbits. The restored tibial and scaffold strengths, assessed using CT intensity, in the PgP group and varied BG@PgP groups, evidenced the effectiveness of bone defect healing across all four groups. The scaffolds demonstrated superior interfacial contact with the margins of the bone defect (Figure 7A). Significant new bone ingrowth was distinctly detected at the scaffold‐bone contact in the sagittal micro‐CT images (Figure S11a). The coronal and transverse views indicated that the predesigned high‐porosity patches, which simulate cancellous bone, in the scaffolds exhibited a heightened tendency for new bone ingrowth. In comparison to the PgP group, all BG@PgP groups exhibited more widespread osteointegration, the development of continuous bone bridges on the surface, and significant new bone production within the scaffolds; this tendency intensified with an increase in BG content of the scaffolds. The osteogenic parameters (Tb.N, Tb.Th, and Tb.Sp) in the BG@PgP groups corresponded with the trends forecasted by the MLP model (Figure S11b). The porous architecture of the scaffolds promoted significant neovascularization in the bone defect region, while the control group displayed a more sparse vascular network and inadequate new bone growth. The statistical examination of the micro‐CT data indicated tendencies that aligned with those observed in the 3D‐reconstructed images. At equivalent radii, the BG@PgP groups exhibited enhanced consistency for vascular volume and length, aligning with the program‐predicted results (Figure S11c). The osteogenic parameters (Tb.N, Tb.Th, and Tb.Sp) in the BG@PgP groups exhibited a correlation with the radiographic findings. Figure 7B illustrates the notable variations in the morphological parameters of BG particles component in the scaffold with differing concentrations. As the BG content increases, the EqD of the particles dramatically reduces (*P* < 0.01), demonstrating that the incorporation of BG effectively governs the microstructural uniformity of the polymer‐grafted polymer matrix. Furthermore, the Ani parameter of particles in the high BG content groups (e.g., 20BG) diminished by almost 35% relative to the control group (PgP) (*P* < 0.001), indicating a tendency towards isotropic particle distribution. Furthermore, the Ecc of the BG particles markedly diminished in all BG@PgP groups (*P < 0.05), thereby substantiating that an elevated BG content enhanced particle dispersion uniformity. Statistical analysis demonstrated a significant negative correlation between BG content and particle morphological parameters (e.g., AF and Ani) (*R* ^2^ = 0.89, *P* < 0.001), indicating that the addition of BG enhanced the microdistribution homogeneity of BG within the scaffold, thus creating a more conducive interfacial environment for new bone ingrowth.

The analysis of microstructural parameters of newly formed bone (Figure 7B) indicated that the area fraction (AF) of newly formed bone in the BG@PgP group reached its maximum at 10% BG, demonstrating that BG substantially enhanced the deposition of new mineralized bone in the defect area. The Ani of the newly created bone markedly increased (*P* < 0.001) in the 10BG group, indicating the production of substantial new and disorganized calli to swiftly establish osseous connections and improve mechanical characteristics. The EqD distribution revealed that the 10BG group had larger osteons and a more dispersed distribution, showing that scaffolds containing 10% BG greatly enhanced the dense and heterogeneous filling of new bone. Moreover, the increased Ecc further substantiated the augmented irregularity of the newly produced bone structure, indicating the fast infilling of the irregular pores inside the scaffold with new bone. Subsequently, the finite element quantitative analysis was performed to examine the pressure variations within the medullary cavity of the animal models following the implantation of the 4D‐printed shape‐memory BG composite scaffolds, in order to elucidate the in vivo biocompatibility of the scaffolds, as illustrated in Figure 7C. Across all groups, including the control group (Figure 7D), the pressure variations were uniform and consistent, with no major encroachment detected, demonstrating exceptional biocompatibility following scaffold implantation. The data correspond with the micro‐CT reconstruction results (Figure 7A) and finite element analysis results (Figure 7C,D), indicating that the BG@PgP scaffolds markedly enhanced the flexibility of the bone regeneration microenvironment due to their optimized microstructure.

## 3 Discussion

(1) This study builds a BG‐incorporated shape‐memory poly (D, L‐lactic acid)‐graft‐poly (ethylene glycol) (SMP‐PDLLA‐g‐PEG) composite scaffold (MABS), and establishes a standardized, mathematical DLN‐driven framework for designing and assessment of materials through multidimensional systematic dataset construction. The use of extensive multidimensional detection and analysis facilitated the collection of data necessary for developing intelligent scaffold design paradigms. MABS designation and validation experiment demonstrated that the 10% BG group exhibited optimal performance in terms of compressive modulus (706.7±30.3 MPa at 20% porosity), osteogenic promotion, and angiogenesis enhancement, thereby substantiating the primary benefit of DLN models in multi‐objective optimization. Diverse models effectively elucidated the nonlinear correlations among the topological properties of BG particles (AF, EqD, Ecc, and Ani) and scaffold performance, attaining an exact equilibrium of mechanical strength, ion release kinetics, and biological response. Cross‐validation and preclinical evaluation were performed using various animal models. This concept addresses the shortcomings of trial‐and‐error methods, offering a sophisticated solution for the repair of irregular bone defects that incorporates dynamic adaptability and functional direction.

(2) Despite utilizing modern 4D printing technology and intelligent materials, the structural and functional design is nevertheless comparable to that of the previous generation. Consequently, it is essential to implement technical methodologies that correspond with the contemporary level of material and scaffold structure design. Typically, scaffold design depends on empirical parameter modifications and static biomimetic modeling, which fail to accommodate the morphological variability of intricate bone defects and dynamic biological microenvironments [46, 47]. The fabrication of biological scaffolds requires a diverse, cohesive engineering framework. Therefore, this study, aimed at predicting real‐world settings and forecasting desirable outcomes, necessitated that the experimental design of the dataset fulfill the following three requirements to guarantee the quality of subsequent analyses and the dependability of research. (1) Adequate sample size, (2) Multidimensionality, and (3) Systematicity.

(3) For the initiating component of the research framework system established in this study, the relevant characterization and detection of BG, which underpins the biological and physicochemical properties of the MABS system, constitute the core and fundamental step for the scaffold fabrication. As illustrated in Figure S1, the BG concentration exerts a threshold‐dependent, nonlinear influence on scaffold performance. In scaffolds with 60% porosity, the 20% BG group exhibited the highest compressive modulus (93.7±10.3 MPa); however, when porosity decreased to 20%, the modulus of the 5% BG group markedly rose to 706.7±30.3 MPa. This contradictory outcome suggests that the filling action of BG particles and the synergistic function of the pore structure necessitate equilibrium via a multi‐objective optimization model. Furthermore, the 10% BG group demonstrated adequate cellular proliferation (BMSCs and HUVECs) and osteogenic differentiation (ALP and ARS staining), while the 20% BG group, due to excessive ion release, resulted in an increased microenvironmental pH that potentially impeded cellular activity (as illustrated in Figure S3b). Research indicates that slightly alkaline environments (about pH 6.7) demonstrate very moderate impacts on cellular activity [43, 44, 45]. Moderately alkaline circumstances (∼pH 8.6) may provide an optimum environment for balancing antibacterial and osteogenic functions, hence boosting osteogenesis without significantly hindering cell growth. A weakly alkaline environment (typical mean pH below 8.2–8.5) can promote mineralization; however, pH values beyond the weak alkaline range considerably impede cell proliferation, necessitating meticulous evaluation in practical applications. Moreover, the DLN model identified that a 10% bioactive glass (BG) content represents the optimal balance between mechanical and biological properties, with a coefficient of determination (R^2^) greater than 0.85, confirming the reliability of the model's predictions. In addition, the model efficiently determined appropriate BG parameters tailored to the desired biological performance, achieving a minimum total error of 0.5578 under the high biocompatibility scenario. Specifically, the optimal BG concentration (11.62) identified for enhanced biocompatibility falls within the previously reported range (10–15), which is known to balance bioactivity and mechanical stability.

(4) Beyond the percentage of BG itself, the intrinsic compositional and micromorphological properties of BG particles exert pivotal biological effects on cellular interactions. Furthermore, 4D scaffolds endow BG particles with spatiotemporal characteristics. Thus, the scaffold's microenvironment experiences dynamic modifications. The mentioned finding suggests that DLN models should integrate dynamic ion release kinetic characteristics to improve the accuracy of biological response predictions. Our ICP‐AES measurements (Figure 2G) demonstrated sustained release of Sr^2+^ and Ca^2+^ from the 10BG scaffolds over 14 days. While these ions are known to promote osteogenic differentiation in vitro [19, 20], the specific competitive dynamics between Sr^2+^ and Ca^2+^ uptake by cells remain to be directly investigated. We therefore hypothesize that the observed osteogenic enhancement may involve, at least in part, ion‐mediated signaling pathways, but further studies (such as ion‐selective imaging or pathway inhibition experiments) are required to establish a causal relationship. The topological properties of the scaffold materials demonstrate synergy. Subsequent analysis using the DLN model evaluated the contribution weights of these parameters to osteogenesis, revealing that anisotropy (Ani) enhances osseointegration by promoting cellular alignment. The consistent optimal values of Ani (0.560) and eccentricity (Ecc, 0.732) across different target combinations indicate that these two parameters synergistically regulate various biological functions. This conclusion is corroborated by correlation analysis, which demonstrates a strong positive correlation between Ani and Ecc (*r* = 0.990). The Ani of BG particles in scaffolds further facilitates osseointegration by encouraging cell alignment (*r* = 0.82), while excessive Ecc diminishes osteogenic efficacy (*r* = ‐0.75). This discovery corresponds with the principles of geometric transformers in protein design, which address local structural limitations, underscoring the critical importance of topological characteristics in functional outcomes. The Pearson correlation analysis indicated a substantial positive association between BG particle's AF and Ani with osteogenic indices (Tb.N and Tb.Th) (*r* = 0.82), whereas Ecc demonstrated a negative correlation (*r* = −0.75). Transcriptomic sequencing demonstrated that 10BG stimulated the Wnt/β‐catenin pathway (upregulation of Ctnnb1 and Lrp5, *q* < 0.001) and angiogenic factors (elevated expression of HIF1A and VEGFA), leading to the dynamic interplay of degradation products, microenvironment regulation, and tissue regeneration. Transcriptomic analysis demonstrated a notable elevation of Ctnnb1, encoding β‐catenin, and LRP5, encoding the Wnt coreceptor, in the 10BG group (log_2_FC > 2), with KEGG enrichment validating that the Wnt pathway was the most significant (*q* < 0.001). The DLN model, through protein–protein interaction (PPI) network analysis, demonstrated the coordinated expression of Spp1 (which encodes osteopontin) and Runx2 (a crucial osteogenic transcription factor), thereby confirming the molecular mechanism by which BG modulates osteogenic differentiation through ion microenvironment alteration. The DLN model incorporates microstructural characteristics (e.g., EqD), transcriptomic data (e.g., overexpression of genes in the Wnt pathway), and macro‐imaging features (micro‐CT bone volume) to establish a multiscale material‐cell‐tissue correlation network. Transcriptomic analysis revealed elevated expression of Wnt/β‐catenin‐associated genes (Ctnnb1 and Lrp5) in the 10BG group compared to PgP controls (*q* < 0.001). This observation is consistent with the known role of calcium‐sensing receptors and β‐catenin nuclear translocation in osteogenesis [40, 41]. However, our data do not prove direct activation of the Wnt pathway by BG degradation products. Therefore, while the correlation is compelling, causal validation (for instance, using pathway‐specific antagonists or gene silencing) should be undertaken in future work. We thus interpret these findings as suggestive of Wnt pathway engagement rather than definitive proof of causality. In addition, active ions and BG particle parameters may influence the integration process between the scaffold and host through contact guidance or local ion distribution. The underlying cellular mechanisms may require further investigation using live‐cell imaging techniques and targeted genetic intervention technologies to elucidate the mechanistic link between microscale particle topography and downstream transcriptional activity. As illustrated in Table 5, compared with widely used experience‐driven scaffold design, the DLN model offers several distinct advantages, including a shortened design cycle, enhanced correlation among nonlinear parameters, superior clinical adaptability, and improved predictability. This capability lays a solid scientific foundation for the systematic fabrication of 4D scaffolds, thereby minimizing the need for laborious and costly trial‐and‐error experiments.

**TABLE 5:** Compared with DLN models and experience‐driven scaffold design.

Indicator	Experimental method	DLN‐driven design
Design cycle	Several months (dependent on iterative experiments)	Several weeks (based on high‐dimensional data modeling)
Parameter correlation	Linear regression (ignoring interaction effects)	Nonlinear MLP (capturing synergistic concentration–porosity–degradation effects)
Clinical compatibility	Static structure (geometric matching degree <60%)	4D dynamic deformation (adaptation degree >90%, Tg 60°C–70°C)
Biological prediction	In vitro experiments dominate (animal validation lags behind)	Cross‐scale modeling (real‐time correlation from molecular pathways to imaging features)

(5) As one of the pivotal properties for osteogenesis, angiogenic potential constitutes a critical component in the evaluation of biomaterials. Accordingly, we have constructed a custom deep learning model integrated with a physical constraint module, which is designed to analyze and interpret the extensive nonlinear and multi‐interactive relationships between angiogenesis and various biological information cues. The modified MLP engine created in this research shows remarkable prediction accuracy (overall *R* ^2^ = 0.944), surpassing earlier machine learning models for BG‐based scaffold design. The incorporation of a Softmax output layer and a constraint loss function guaranteed complete adherence to the biological feasibility constraint (bio + osteo + angio = 100%), overcoming a significant shortcoming of unconstrained models that may produce predictions inconsistent with physiological plausibility. This study adopted the three well‐defined and clinically relevant indicators as primary evaluation endpoints. Complex influencing factors, including inflammatory osteogenesis and neurogenic coupling were not incorporated at this stage, so as to prevent research divergence. Such progressive research design conforms to conventional advanced material development principles. Fundamental structure‐property correlations were verified through localized optimization before further multi‐dimensional exploration assisted by machine learning algorithms [48, 49]. This stringent constraint mechanism augments the scientific rigor of the model. The superior *R* ^2^ score for biocompatibility (0.983) in contrast to angiogenesis (0.893) indicates a heightened sensitivity of biocompatibility to BG concentration (average sensitivity = 0.1394 against 0.0659 for angiogenesis). This finding aligns with prior research demonstrating that BG concentration is a primary factor governing cell growth and adhesion. The moderate prediction error for angiogenesis (MAPE = 9.69%) can be ascribed to the increased complexity of angiogenic pathways, which encompass a greater number of biological markers (10 compared to 4 for biocompatibility) and more pronounced non‐linear interactions. We employed multiple angiogenesis models to detect the proangiogenic properties of the scaffolds. Three‐dimensional vascular reconstruction analysis in a rat calvarial defect model demonstrated that bioactive glass (BG) at various concentrations in all groups effectively promoted angiogenesis, yet no statistically significant differences were observed among the groups. In contrast, significant intergroup differences were identified in both the ectopic osteogenesis model and the rabbit tibial defect model. Such discrepancies could not be adequately explained merely by the specificity of the experimental models. Therefore, the aforementioned deep learning model is capable of acutely capturing the key factors and subtle perturbations in angiogenesis, thereby further optimizing scaffold design and biological activity.

(6) Notwithstanding its advantages, this design possesses multiple restrictions. First, the model's prediction range is constrained by the training data (e.g., BG concentration: 5–20), thereby limiting its applicability to extreme parameter values. Second, the opaque nature of deep learning impairs model interpretability, which could be improved by adopting explainable AI (XAI) methodologies such as SHAP or LIME. Despite these limitations, the design incorporates several notable features that enhance its rigor and robustness in the field of biomaterials. To address the above key issues, the model incorporates three critical deep learning modules: a forward prediction module, a physically constrained inverse prediction module, and an incremental learning module. The incremental learning module addresses a critical challenge in AI models for material science: the ability to adapt to new experimental data without requiring complete model retraining. By utilizing experience replay with a memory bank of 500 samples, the model retains prior knowledge while integrating new information, resulting in a 3.6% improvement in performance. This feature is particularly valuable for scaffold construction, as experimental data in this area are often collected incrementally. Additionally, the web application enhances accessibility, enabling academics and engineers to utilize the model without specialized programming expertise—aligning with the growing trend of user‐friendly AI tools in material science. Furthermore, the DLN model integrates multispecies data (from rats and rabbits) through transfer learning, achieving significantly higher prediction accuracy (*R* ^2^ = 0.891) compared to traditional finite element analysis (*R* ^2^ < 0.65), thus providing a reliable tool for clinical translation. To conduct a comprehensive analysis of the acquired data, two additional machine learning models were employed. Notably, the analytical results demonstrate that all three models—MLP, random forest, and XGBoost—exhibit excellent performance, albeit with distinct characteristics. Collectively, the multi‐model comparison results validated the superior capability of the MLP neural network in structured data processing, establishing MLP as the core architecture of the proposed predictive model.

(7) To further validate the robustness of the customized model, a set of commonly used machine learning models were adopted for parallel verification to conduct a comparative analysis of their performance. The MLP model exhibits reduced values for metrics including MSE, RMSE, MAE, MAPE, and SMAPE, along with an elevated R‐squared, signifying diminished prediction errors and a tighter correspondence between predictions and actual values. The R‐squared coefficient of determination is 0.945, nearing 1, indicating that the model proficiently accounts for data variability and exhibits robust fitting performance. The greatest error value is minimal, signifying that the model has good predictive accuracy even in harsh conditions. In the random forest model, the MSE, RMSE, MAE, MAPE, SMAPE, and R‐squared metrics are marginally elevated compared to those of the MLP model, indicating somewhat diminished accuracy. The R‐squared coefficient of determination is 0.943, akin to that of the MLP model, signifying equivalent fitting performance. The greatest error value is comparable to that of the MLP model, indicating consistent performance under harsh conditions. In summary, the random forest model has commendable performance with considerable reliability, however it is marginally less effective than the MLP model. The XGBoost model exhibits more MSE, RMSE, MAE, MAPE, SMAPE, and R‐squared metrics compared to the MLP and random forest models, signifying larger prediction errors. The R‐squared coefficient of determination is 0.933, indicating worse data fitting power compared to the other two models. The maximum error value is elevated, indicating possible prediction discrepancies in some severe situations. In summary, although the XGBoost model has commendable performance, its accuracy and consistency are marginally less than those of the MLP and random forest models. This study utilizes the MLP neural network model to forecast BG microparameters at different BG percentages. The dataset utilized to train the MLP neural network is employed to develop an interface program for forecasting the biocompatibility, osteogenic potential, and angiogenic characteristics of BG particles.

(8) Upon validating the machine learning models, this work subsequently employed the optimal mathematical model results derived from the training of the MLP model for the design and validation of future scaffolds. The DLN‐corrected 4D‐printed MABS showed remarkable efficiency in bone regeneration inside a rabbit tibial defect model. Micro‐CT imaging and 3D reconstruction revealed significant new bone growth at the interface between the scaffold and bone, especially in the scaffold's designated high‐porosity areas (mimicking cancellous bone), where new bone development was notably enhanced. These findings demonstrate that DLN‐corrected scaffolds provide superior mechanical support and significantly enhance bone tissue regeneration. The DLN‐corrected scaffolds demonstrated enhanced osseointegration in the bone defect region, establishing continuous bone bridges and producing substantial new bone within the scaffold's interior. The bone volume fraction (BV/TV) in the DLN‐optimized group was markedly superior to that of the control group (40.4% ± 3.6% vs. 9.72% ± 3.2%), hence validating the model's efficacy in assessing the osteoinductive capabilities of the scaffolds. Furthermore, the DLN‐corrected scaffolds exhibited a dense, interconnected vascular network, with novel blood vessels infiltrating the whole bone defect region. Histomorphometric analysis indicated that the optimized scaffold group exhibited 2.8±2.0 additional vascular branches, reflecting a 1.4‐fold enhancement relative to the control group (*p* < 0.01). The findings indicated that the DLN‑corrected scaffolds not only facilitated bone regeneration but also improved angiogenesis. Notably, the angiogenic efficacy of the 10BG scaffold was robust across all three models (in vitro HUVECs, rat calvarial, and rabbit tibial), whereas the 5BG and 20BG scaffolds displayed more variable outcomes: 5BG promoted angiogenesis in vitro and in the rat model but showed weaker effects in the rabbit tibial defect, while 20BG enhanced vessel density in the rat model but was inhibitory in prolonged HUVEC cultures and exhibited negative correlations with vascular quality parameters. These cross‑model differences underscore the importance of using multi‑parametric and multi‑species evaluations to accurately assess angiogenic potential. The consistency of the 10BG scaffold's pro‑angiogenic effect across all platforms, however, provides strong evidence that the DLN‑optimized intermediate concentration achieves a favorable balance of ion release and pH modulation, thereby reliably supporting vascularization during bone repair.

(9) Despite the considerable promise of the DLN‐4D printing system, certain difficulties persist that require resolution. The existing model relies on a constrained dataset, potentially leading to overfitting of batch effects. It is essential to broaden cross‐pathology models (e.g., osteoporosis and viral bone abnormalities) and integrate active learning methodologies. The current model rigidly links parameters to results, neglecting time‐dependent deterioration kinetics. Future endeavors will concentrate on three avenues: (1) augmenting the dataset to encompass a broader array of BG compositions and biological contexts to enhance generalizability; (2) incorporating multi‐modal data (e.g., micro‐CT imagery, mechanical testing metrics) to improve predictive precision; and (3) establishing a cloud‐based infrastructure to facilitate real‐time collaboration and extensive data processing. Furthermore, refining the reverse optimization technique with multi‐objective functions (such as integrating cost and manufacturing feasibility) will augment its industrial application.

(10) A critical consideration for the clinical translation of our SMP‐based MABS is the thermal safety of the shape‐recovery process, which is triggered by ∼60°C warm saline. Several lines of indirect evidence suggest a potential safety window that warrants further systematic investigation. First, dynamic mechanical analysis indicated that the glass transition temperature (Tg) of our composites is situated between 60°C–70°C, which is below the threshold for immediate thermal necrosis. Ito et al. demonstrated that rat femoral cortical bone retains its osteoinductive capacity after heating at 70°C for 1 h, with significant damaging effects only becoming apparent above 90°C [17]. This finding provides a critical biological rationale for our system's operation temperature. Second, the heat stimulus is applied with a limited volume of saline in a highly localized manner for only a brief period, effectively making it a ‘warm rinse’ rather than a sustained thermal bath. Third, and most importantly, our in vivo histological analysis (Figure 5F) revealed no visible signs of thermal injury, such as tissue charring, necrosis, or exacerbated inflammatory infiltration in the peri‐scaffold tissue, at 4 or 8 weeks post‐surgery. While these observations are supportive, we fully agree that they do not constitute a definitive safety validation. A more rigorous investigation employing in situ real‐time thermal probes during the recovery process is essential to map the spatiotemporal thermal landscape. This will be a core aim of our follow‐up studies, which will focus on defining the precise ‘thermal dose’ to ensure safe and effective clinical application, making this a critical limitation of the present work.

(11) This study introduces a data‐driven paradigm for smart material and bioactivity that presents three improvements in the domain of bone regeneration. Initially, 4D‐printed scaffolds employ body temperature to activate shape memory effects, resulting in a reduction of the storage modulus by two orders of magnitude, hence facilitating real‐time dynamic alignment with defect morphology and obviating the necessity for later surgical modifications. Second, the DLN model facilitates quantitative forecasting of ion release profiles (competitive Sr^2^^+^/Ca^2^^+^ kinetics) and directional enhancement of the osteogenic microenvironment through the modification of BG topological characteristics, resulting in accurate regulation. Ultimately, by incorporating patient CT/MRI data, the joint learning framework can produce individualized medical solutions, enhancing bone healing from uniformity to accuracy. In the future, the profound integration of DLN and 4D printing will lead to self‐evolving smart materials; through real‐time feedback and adaptive optimization, scaffolds can dynamically modify their mechanical strength and bioactivity in vivo, ultimately achieving zero‐intervention bone regeneration. This vision will transform the design parameters of biomaterials and create a new framework for regenerative medicine.

## 4 Methods

### 4.1 Materials

The shape‐memory poly (D,L‐lactic acid)‐graft‐polyethylene glycol copolymer, designated as PgP. Polyethylene glycol (PEG, molecular weight 1000), trichloro(1H,1H,2H,2H‐perfluorooctyl) silane, benzoyl peroxide (BPO), and trichlorosilane were purchased from Sigma‐Aldrich. Polydimethylsiloxane (PDMS) prepolymer and curing agent (Sylgard 184) were purchased from Dow Corning Holding. All testing and purification procedures were performed using alcohol and deionized water.

### 4.2 Fabrication and Characterization of 4D Printed Microenvironment Adaptive Bioactive Scaffold

The 4D printed microenvironment adaptive bioactive scaffold (MABS) is composed of PgP and 1393‐BG. The commercial silicate‐based bioactive glass with a specific composition (wt. %) (6 Na_2_O, 12 K_2_O, 5 MgO, 18 CaO, 38 SiO_2_, 17 B_2_O_3_, 4 P_2_O_5_), as cited in Patent US20080066495A1 and identified as 1393‐BG, was synthesized via a high melting technique by combining analytical grade Na_2_CO_3_, K_2_CO_3_, MgCO_3_, CaCO_3_, SiO_2_, H_3_BO_3_ and NaH_2_PO_4_·2H_2_O (National Pharmaceutical Chemical Reagent Co., Ltd., Shanghai, China) at approximately 1250°C for about 1 h. Afterward, the glass melt was quenched in corrosion resistant plates to form BG raw particles. With further processing involving crushing, ball milling, and sieving, BG particles less than 10 µm were acquired. The shape memory property of PgP enables MABS to adapt to complex bone defects, while 13393‐BG imparts MABS with the essential ionic and alkaline microenvironment (IAM) to modulate the local osteogenesis and angiogenesis. The MABS is initially constructed into the designed configuration via hot‐melt printing technology, and its structure can be subsequently reprogrammed at the glass transition temperature, therefore revealing the advantage of 4D printing, as illustrated in Figure 1.

#### 4.2.1 Preparation of BG@PgP Melt‐Extruded Printing Composite Filament

A mixture consisting of PgP particles with a mass ratio of 10 PDLLA:1 PEG was obtained by utilizing a blend system (3DPANY). Subsequently, 4 g of the acquired PgP particles were dissolved in 50 mL of chloroform at ambient temperature with continuous magnetic stirring until a homogeneous transparent solution was achieved. Then 1393‐BG powder was integrated into the previously indicated transparent solution at concentrations of 0, 5, 10, and 20 wt.%. This mixed solution was subsequently exposed to ultrasonic dispersion for 30 min to attain a homogeneous distribution of 1393‐BG. After that, the mixed solution was placed in a constant‐temperature oil bath apparatus under nitrogen protection, where it was continuously stirred at 65°C for 6 h. Following the agitated reaction, the mixed solution was transferred into sterile glass culture dishes and exposed to a 37°C vacuum drying oven for 72 h. After the whole evaporation of chloroform, composite films with a thickness of approximately 0.3 mm were produced.

To satisfy the material form criteria for melt extrusion printing, the acquired composite film obtained in the previous step was subsequently sectioned into uniform fragments measuring 5×5 mm, meticulously cleaned three times with anhydrous ethanol and deionized water to eliminate surface residues, and dried in a vacuum oven at 60°C afterward until a constant weight was attained. A single‐screw extruder (3DPANY) was then utilized for high‐temperature melt processing, with the following equipment parameters: extrusion temperature at 175°C, screw rotation speed at 60 r/min, and feeding screw speed at 10 r/min. The fragments were uniformly melted by the screws, followed by instantaneous water cooling and drawing after departing the extrusion end, resulting in continuous BG@PgP composite filaments with a diameter of 1.75 mm ± 0.02 mm.

#### 4.2.2 Structural Design and Fabrication of 4D Printed MABS

The 3D bracket model was designed using SolidWorks 2022 software, and the printing path was planned with Ultimaker Cura 5.0 slicing software. A fused deposition modeling printer (ANYCUBIC i3 MEGA) was used to produce 4D‐printed MABS with the detailed composition listed in Table 6, using BG@PgP composite filaments prepared in Section 4.2.1. Layer‐by‐layer deposition was conducted at a nozzle temperature of 190°C and a heated bed temperature of 60°C, utilizing a layer height of 0.2 mm and a forming speed of 30 mm/s. Exact regulation of porosity was attained by modifying the filling density between 40% and 80%, resulting in bone tissue engineering scaffolds that integrate mechanical strength with linked porous architectures.

**TABLE 6:** Compositions of 4D printed microenvironment adaptive bioactive scaffold (MABS).

Group designations	Compositions	
	1393‐BG [wt. %]	PLS SMP [wt. %]
PgP	0	100
5%BG@PgP (5BG)	5	95
10%BG@PgP (10BG)	10	90
20%BG@PgP (20BG)	20	80

### 4.3 Construction of an Dln Deep Learning Datasets for Analyzing the Interaction Between Composition of MABS and Their Biological Performances in Materials Science

Experimental scaffold design depends on empirical parameter modifications and static biomimetic modeling, hindering the ability to accommodate the morphological variability of intricate bone defects and dynamic biological microenvironment. Consequently, our research amalgamates machine learning (MLP neural networks) with 4D printing technology to surmount the constraints of conventional scaffold design reliant on empirical parameter optimization. Initially, multi‐category training and validation data (e.g., BG morphological parameters, transcriptomic features, radiomics, etc.) were acquired from 4D printed MABS with empirical designations of PgP, 5BG, 10BG, 20BG, measuring 10 mm in diameter and 3 mm in height. Subsequently, empirical parameters including AreaFraction (AF), EqDiameter (EqD), Eccentricity (Ecc), Anisotropy (Ani) were fed to DLN to conduct the deep learning, therefore enabling the extraction of material characteristics, formulation of predictive standards and protocols, optimization of the design workflow, and development of a nonlinear correlation model that associates properties of MABS with its biological performances. Ultimately, the optimal design programs of MABS were output, and the osteogenic efficacy of this best formulation of MABS was further validated both in vitro and in vivo.

#### 4.3.1 Dataset Collection and Preprocessing

The dataset for this study consisted of 2,564 experimental samples of 4D bioactive glass (BG)‐based scaffolds, encompassing 5 key BG material parameters (BG concentration, AF, Ani, Ecc, EqD) and 18 biological performance indicators. The biological indicators were categorized into three functional groups based on their biological roles: biocompatibility, osteogenesis, and angiogenesis. The original dataset formed a matrix containing 58972 data points. Among them, 2328 values from 388 specimens across six indicators were absent. Column‐wise random imputation was adopted to fill gaps and attain full data integrity. Missing values mainly occurred in six indicators, covering morphological indices scr, tlength, tvolume, tnodes, and uarea, along with the osteogenic functional marker ars. To guarantee reliable data quality, column‐wise random imputation served as the main filling method. Meanwhile, multiple imputation via chained equations (MICE) was further conducted for sensitivity comparison and diagnostic analysis. After imputing, preprocessing and splitting, the resulting train.txt, val.txt, and test.txt files were confirmed to achieve 100% data integrity, containing no missing values, blank entries, or anomalous records [35].

Outliers were identified and processed using the interquartile range (IQR)‐based capping method to preserve data distribution while eliminating extreme values. All features were standardized via Z‐score normalization to mitigate the impact of dimensional differences on model training [50]. The dataset was randomly split into a training set (1,794 samples, 70%), a validation set (385 samples, 15%), and a test set (385 samples, 15%) with a fixed random seed (42) to ensure reproducibility [51]. Random within‐column imputation was applied exclusively to the training set before model fitting, as it relies solely on the marginal distribution of each column and does not exploit inter‐sample relationships or label information; thus, information leakage is negligible. Strict isolation was enforced thereafter: the validation and test sets remained completely untouched during imputation and model training, ensuring unbiased evaluation of generalization performance. Moreover, the test set was kept completely confidential and applied only to final model evaluation, thus avoiding information leakage in training and hyperparameter tuning processes [35, 52].

#### 4.3.2 MLP Model Architecture and Training

A MLP deep learning model was constructed to establish the mapping between BG parameters and biological performance. The network architecture comprised an input layer (5 neurons), five hidden layers (512→256→128→64→32 neurons), and an output layer (3 neurons). The model contained a total of 177,731 trainable parameters. In bone tissue engineering, biocompatibility, osteogenesis, and angiogenesis are universally recognized as the three foundational pillars for evaluating scaffold efficacy [53, 54]. Thus, ReLU activation was used for hidden layers to introduce non‐linearity, while a Softmax layer was appended to the output to enforce the constraint that the sum of bio, osteo, and angio equals 100% [55].

Three evaluation outputs were calculated based on 23 experimental parameters consisting of 5 scaffold design parameters and 18 biological indicators. Biocompatibility, osteogenic, and angiogenic composite scores were separately computed by grouping relevant measurement indices. All composite scores were normalized using a numerically stable Softmax function to generate a probability distribution with a total sum of 1.0. This structural design inherently met the summation constraint, thus removing the requirement of extra constraint loss terms. A weight range of 0–1000 was adopted for comparative verification, proving that the Softmax‐based structure attained constraint compliance above 99.9% without explicit penalty settings. The proposed multi‐task probabilistic constraint framework complies with uncertainty‐weighted strategies in deep multi‐task learning [56, 57], and its mathematical principle originates from compositional data analysis [58].

Training was performed using the Adam optimizer (learning rate = 0.001, batch size = 32) with a combined loss function: MSE for prediction accuracy and a constraint loss term (weight = 1000) to reinforce the sum constraint [59]. The model was trained for 300 epochs with early stopping (patience = 100) to prevent overfitting, and a dropout rate of 0.2 was applied to enhance generalization [60].

#### 4.3.3 Forward Prediction, Reverse Optimization and Incremental Learning

Forward prediction, the trained model was used to predict the three biological performance indicators (bio, osteo, angio) from input BG parameters. Prediction accuracy was evaluated using *R* ^2^ score, MAE, RMSE, MAPE, and SMAPE [61].

Reverse optimization was performed using the Broyden‐Fletcher‐Goldfarb‐Shanno (BFGS) algorithm from the SciPy library to infer the optimal BG parameters corresponding to the target biological performance metrics. The objective function was defined as the minimization of MSE between predicted and target values, with constraints limiting BG parameters to the experimental data range (BG: 5–20, AF: 0–0.000199, Ani: 0.035–0.988, Ecc: 0.186–0.994, EqD: 1.954–19.899) [62].

An incremental learning module was integrated to enable continuous model updates with new experimental data. A memory bank (size = 500 samples) was used for experience replay, and the model was fine‐tuned with a small learning rate (0.0001) and mini‐batch training (batch size = 16) for 20 epochs to avoid catastrophic forgetting [63].

#### 4.3.4 Virtual Experiment Validation and Web Application Deployment

Fifty virtual experiment samples were generated from the test set to validate model reliability. Prediction accuracy was assessed via MAE, RMSE, and *R* ^2^, while statistical significance was confirmed using paired t‐tests (*α* = 0.05) [64]. Constraint satisfaction was verified by checking that the sum of predicted bio, osteo, and angio remained within ±0.1% of 100%.

A user‐friendly web interface was developed using HTML5, CSS3, JavaScript (frontend) and Flask RESTful API (backend) to deploy the AI prediction system. The interface supports forward prediction, reverse optimization, incremental learning, and real‐time constraint validation, with responsive design for desktop and mobile devices [65].

#### 4.3.5 The Morphological Parameters of 1393‐BG in MABS

Micro‐CT analysis was utilized to conduct Cross‐sectional thin‐layer imaging of the 4D printed MABS with empirical designations of PgP, 5BG, 10BG, and 20BG, measuring 10 mm in diameter and 3 mm in height. Upon acquisition of the scan data, computer‐aided methodologies were employed to reconstruct their structural characteristics. The obtained data were refined utilizing mean and Gaussian algorithms, and a labeled mask for the structure of the 4D printed MABS was generated through binarization techniques. Then a 3D solid model of the 4D printed MABS was produced from this mask, and a thickness analysis was conducted to produce a thickness heatmap of the internal structure of MABS, accompanied by pertinent data analysis. Ultimately, the surface morphology of the 4D printed MABS was presented. Subsequently, custom python programs, a widely used image analysis software, were employed to process the cross‐sectional thin‐layer scan data of the 4D printed MABS acquired from Micro‐CT analysis. A median filter module was utilized on the image data to efficiently remove noise interference and artifacts. The input image data was then segmented into regions exhibiting analogous features by utilizing the intensity auto‐classification module. Following the execution of manual thresholding, a 3D threshold was implemented on the segmented image to isolate brighter pixels in the subtraction output. The intensity auto‐classification module was again utilized to differentiate the distribution of 1393‐BG particles out of PgP substrate based on the variance in signal intensity between 1393‐BG and PgP. Interactive binarization segmentation was reapplied afterward to identify 1393‐BG particles, and a mask was incorporated. Subsequently, a 3D solid model of MABS was created utilizing the mask, while the labeled mask was examined for internal 1393‐BG particle composition and morphological features.

#### 4.3.6 The Phase, Chemical and Microstructure Characteristics of MABS

The crystalline phase of 4D printed MABS with empirical designations of PgP, 5BG, 10BG, 20BG, measuring 10 mm in diameter and 3 mm in height, was analyzed using X‐ray diffraction (XRD) over a diffraction angle (2θ) range of 5° to 75° at a scanning rate of 2°/min. MABS specimens require vacuum heat treatment at 90°C for 4 h before XRD testing. This procedure aims to ensure the complete crystallization of MABS under high‐temperature conditions. Their microstructures were analyzed using scanning electron microscopy (SEM). Energy‐dispersive spectroscopy (EDS) and Espirit 1.9 software were employed in conjunction with SEM to examine the surface elemental distribution of MABS.

#### 4.3.7 The Mechanical Properties of MABS

The mechanical strength of implants is crucial for preserving structural stability in bone regeneration defects. This study employed a universal mechanical testing machine (Shimadzu Corporation) to precisely evaluate the compressive strength and modulus of MABS. The standard testing samples of MABS with empirical designations of PgP, 5BG, 10BG, and 20BG, measuring 15 mm in diameter and 15 mm in height, conform to ISO 604:2002, which is the International Organization for Standardization's norm for non‐metallic materials. The MBSA sample groups with porosity criteria of 20% and 60% were employed for compressive strength testing, respectively. The initial stress‐strain curves were examined to determine the corresponding compressive strength and elastic modulus.

#### 4.3.8 The Shape Memory Performance of MABS

The shape recovery characteristics of 4D printing scaffolds at the glass transition temperature are crucial for assessing their shape memory performance, while dynamic thermomechanical testing (DTM) provides a direct evaluation of the shape recovery abilities, in accordance with ISO 10993‐12:2012. Therefore, MABS samples with empirical designations of PgP, 5BG, 10BG, 20BG, measuring 40 mm in length, 5 mm in width, and 1 mm in thickness, were subjected to DTM across a temperature range of 0°C–160°C, with a heating rate set at 5°C per minute. The loading frequency of the DTM was set at 1 Hz, with a vibration amplitude of 5 µm.

#### 4.3.9 Ion Release, Capacity for Alkaline Microenvironment Formation, and Biomineralization Characteristics of MABS

To investigate the ion release profile, capacity for alkaline microenvironment formation, and biomineralization characteristics of MABS, scaffold samples with empirical designations of PgP, 5BG, 10BG, 20BG, and measuring 5 mm in diameter and 2 mm in thickness were immersed in PBS and subsequently subjected to a constant‐temperature oscillator set at 37°C with an oscillation frequency of 70 r/min. The scaffolds were collected at 24 h, 72 h, and one week, with corresponding leachates obtained at each time point. The volume ratio of PBS to scaffold samples was determined according to the reference [ISO 10993‐1]. The ionic release profiles in PBS were evaluated using Inductively Coupled Plasma‐Atomic Emission Spectrometry (ICP‐AES). Surface pH variation at 100 µm above MABS after immersion in PBS was assessed using non‐invasive micro‐test technology (NMT100/200 series, Younger USA LLC, MA) (*n* = 4). To assess biomineralization characteristics, MABS was immersed in simulated bodily fluid (SBF) for 3, 7, and 14 days, employing XRD analysis in accordance with the parameters outlined in the section titled “T*he phase, chemical and microstructure characteristics of MABS*.”

### 4.4 The Interaction Between Composition of MABS and the Performances of Osteogenesis of BMSCs and Angiogenesis of HUVECs In Vitro

#### 4.4.1 Preparation of MABS Extracts

According to evaluation groups: PgP, 5BG, 10BG, 20BG. In accordance with the requirements of ISO 10993‐12:2012, the scaffolds from each group were immersed in tissue culture medium at a ratio of 0.1 g/mL at 37°C for 24 h. After standing, the upper layer of the extract was taken for partial cell co‐culture experiments.

#### 4.4.2 Cell Culture

Under sterile conditions, the murine BMSCs cell line (immortalized mouse bone marrow mesenchymal stem cells, product code: iCell‐0116a) and human umbilical vein endothelial cells (HUVECs, product code: CRL‐1730) were thawed. For cell culture, complete culture medium was prepared using DMEM (ScienCell, USA) or ECM (ScienCell, USA) supplemented with 10% fetal bovine serum (HyClone, USA) and 1% penicillin/streptomycin (Beyotime, China), respectively. BMSCs were cultured using DMEM medium, and HUVECs were cultured using ECM medium. The cell clusters were then uniformly distributed and inoculated into culture flasks, and incubated at 37°C, 5% CO_2_, and 95% humidity. The media were substituted after 24 h. After orderly rinsed with 70% ethanol and PBS for 30 min, MABS samples with empirical designations of PgP, 5BG, 10BG, 20BG, and measuring 10 mm in diameter and 3 mm in height, were sterilized by UV radiation for 30 min and subsequently dried at 90°C for 1 h.

#### 4.4.3 The Composition of MABS Mediating the Viability, Proliferation and Osteogenesis of BMSCs

*Cell morphology of BMSCs following the incubation on the surface of MABS*: To investigate the cell adhesion after culture with MABS, BMSCs at a density of 2 × 10^3^ cells per well were seeded on the surface of MABS samples (measuring 10 mm in diameter and 3 mm in thickness) with empirical designations of PgP, 5BG, 10BG, 20BG. Following a culture time of 4 h in 24‐well plates at 37°C, culture media were sustained with a growth media and continuously incubated for another 3 days of interval. After that, the MABS samples with BMSCs adhesion were retrieved and rinsed with PBS for 2–3 times, and subsequently immersed in glutaraldehyde for fixation at 4°C overnight. After the elimination of glutaraldehyde, the MABS samples were then dehydrated in a series of gradient alcohol solutions including 50%, 60%, 70%, 80%, 90%, 95% for 15 min each, and air‐dried at ambient temperature afterward. Ultimately, the MABS samples with BMSCs adhesion were sprayed with gold and analyzed under a scanning electron microscope (SEM) to assess the adherence and morphological features of BMSCs on the surface of MABS. To examine cell adhesion following culture with MABS, BMSCs were inoculated onto the surfaces of MABS samples designated as PgP, 5BG, 10BG, and 20BG, each measuring 10 mm in diameter and 3 mm in thickness. After a culture period of 4 h in 24‐well plates at 37°C, the culture media were supplemented with DMEM (ScienCell, USA), and BMSCs were incubated continuously for a further 3 days. Subsequently, the MABS samples with BMSCs adherence were retrieved and washed with PBS two to three times, followed by immersion in glutaraldehyde for fixation at 4°C overnight. After the removal of glutaraldehyde, the MABS samples underwent dehydration using a series of gradient alcohol solutions at concentrations of 50%, 60%, 70%, 80%, 90%, and 95%, each for 15 min, followed by air‐drying at ambient temperature. The MABS samples with BMSCs adhesion were coated with gold and examined using a scanning electron microscope (SEM) to evaluate the adherence and morphological characteristics of BMSCs on the MABS surface.

*Cytotoxicity and proliferation of BMSCs following incubation with MABS*: BMSCs at a density of 2 × 10^3^ cells per well, were cultured with MABS samples (measuring 10 mm in diameter and 3 mm in thickness) with empirical designations of PLA, 5BG, 10BG, 20BG, in 96‐well plates, and culture medium was used as the blank control. After incubation at 37°C for 24, 48, and 72 h, the culture media were substituted with new medium containing 10% (v/v) CCK‐8 reagent (Beyotime, China) at each designated time point, followed by an additional 2 h incubation under the same conditions. Subsequently, absorbance of culture media was quantified at 450 nm utilizing a spectrophotometric microplate reader (Bio‐Rad 680, Berkeley, USA). Each experimental group was evaluated in 6 replicates at each time point, with data presented as mean values and standard deviations.

*Live/dead staining of BMSCs following the incubation on the surface of MABS*: BMSCs at a density of 2 × 10^3^ cells per well, were cultured on the surface of MABS samples (measuring 10 mm in diameter and 3 mm in thickness) with empirical designations of PgP, 5BG, 10BG, 20BG, and culture medium was used as the blank control. Following culture in 24‐well plates at 37°C for a duration of 4 h, a suitable growth media, DMEM medium, was supplemented to replace the original culture media. After an additional 7‐day incubation period, the viability of BMSCs was assessed utilizing the Calcein AM/PI staining kit (Beyotime, China). Live cells displayed green fluorescence, while dead cells were marked by red fluorescence.

*ALP staining assay of BMSCs following incubation with MABS*: BMSCs at density of 10^5^ cells per well, were cultured with MABS samples (measuring 10 mm in diameter and 3 mm in thickness) with empirical designations of PgP, 5BG, 10BG, 20BG in 6‐well plates, and culture medium was used as the blank control. The culture media was renewed every two days. At incubation periods of 5, 7, and 10 days, the ALP activity of BMSCs was stained by using the BCIP/NBT Alkaline Phosphatase Kit (Beyotime, China). BMSCs were first fixed with 4% paraformaldehyde and subsequently incubated in BCIP/NBT solution at 37°C for 30 min. Thereafter, BMSCs in each group were rinsed thrice with PBS, and the ALP staining results were recorded by utilizing a phase‐contrast microscope (Carl Zeiss, Germany).

*Cellular mineralization assay of BMSCs following incubation with MABS*: To evaluate the cellular mineralization capacity of BMSCs, calcium nodule deposition of BMSCs was assayed by culture with MABS samples (measuring 10 mm in diameter and 3 mm in thickness) in empirical designations of PgP, 5BG, 10BG, 20BG, and culture medium was used as the blank control. Following the incubation period of 21 days at density of 10^5^ cells per well in 6‐well plates with medium alterations every two days, BMSCs were fixed with 4% formaldehyde and ordely washed with 1000 µL of PBS. Subsequently, BMSCs were treated with Alizarin Red (Cyagen, China) for 3 min and then rinsed with double‐distilled water to remove any surplus dye. Then the stained calcium nodules were recorded by utilizing a phase‐contrast microscope.

*Osteogenic related genes expression of BMSCs following incubation with MABS*: Osteogenic genes of BMSCs, including Bglap2, Runx2, Spp1, Ctnnb1, Lrp5, following culture with MABS samples (measuring 10 mm in diameter and 3 mm in thickness) in empirical designations of PgP, 5BG, 10BG, 20BG, were evaluated via RT‐qPCR, with the culture medium serving as the blank control. Following a 24 h pre‐culture in DMEM, BMSCs at a density of 10^5^ cells per well in 6‐well plates, were inoculated in DMEM supplemented of the osteogenic induction medium comprising dexamethasone, ascorbic acid, and β‐glycerophosphate. On the seventh day of cultivation, the cells were lysed, followed by the study of osteogenic‐related gene expression. Total RNA was extracted with TRIzol (Thermo Fisher Scientific, USA), and complementary DNA (cDNA) was synthesized with the Takara reverse transcription kit, the Biomake SYBR Green kit, and Thermo PCR equipment, following the manufacturers' instructions. The primer sequences for the target genes were listed in Table 3, with Gapdh serving as the housekeeping gene.

#### 4.4.4 The Composition of MABS Mediating the Viability, Proliferation and Angiogenesis of HUVECs

*Cytotoxicity and proliferation of BMSCs following incubation with MABS*: In accordance with the technique outlined in the section titled “*Cytotoxicity and proliferation of BMSCs following incubation with MABS*”, the cytotoxicity of HUVECs was assessed at cultivation periods of 24, 48, and 72 h using the CCK‐8 method, with the culture medium serving as the blank control.

*Live/dead staining of HUVECs following the incubation on the surface of MABS*: In accordance with the technique outlined in the section titled “*Live/dead staining of BMSCs following the incubation on the surface of MABS*”, the viability of HUVECs was assessed at cultivation periods of 7 days utilizing the Calcein AM/PI staining kit.

*Tube formation of HUVECs following incubation with MABS extracts*: HUVECs at a density of 2 × 10^3^ cells per well were cultured with extracts of MABS in empirical designations of PgP, 5BG, 10BG, 20BG to evaluate their capacity for tube formation, utilizing the endothelial cell tube formation assay (ECTFA), with the culture medium serving as the blank control. HUVECs were cultured on 24‐well plates pre‐coated with Matrigel Matrix Growth Factor Reduced (BD Biosciences, USA) alongside extracts from each empirical designation. Following a 4 h incubation at 37°C, the formation of pseudocapillary network during the ECTFA was recorded by a phase‐contrast microscope and quantitatively assessed utilizing the angiogenesis analysis plugin of ImageJ software (National Institutes of Health, USA). The number of tube branches was counted using an inverted phase‐contrast microscope, the tube length was assessed in pixels, and the meshes, nodes, and branches of the capillary network were quantified in five random fields per well. *Scratch assay of HUVECs following incubation with MABS extracts*: HUVECs at a density of 1×10^3^ cells per well were cultured with extracts of MABS in empirical designations of PgP, 5BG, 10BG, and 20BG to evaluate their migration capacity, with the culture medium serving as the blank control. Upon achieving complete confluence of HUVECs in 6‐well plates, a linear incision was executed in the central area of each well. Subsequently, HUVECs were cultured with extracts of MABS and microscopically recorded immediately and 24 h post‐scratch to evaluate the advancement of cell migration. The alterations in wound healing width were quantitatively assessed by measuring the distance between the scratch edges.

*Angiogenic related genes expression of HUVECs*: The angiogenic‐related genes of HUVECs, including HIF‐1α and VEGFA, following culture with MABS samples (measuring 10 mm in diameter and 3 mm in thickness) in empirical designations of PgP, 5BG, 10BG, and 20BG, were evaluated via RT‐qPCR, with the culture medium serving as the blank control. HUVECs were cultivated at a density of 4 × 10^5^ cells per well in ECM medium at 37°C. Gene expression analysis was conducted on day 7. Total RNA was extracted with TRIzol, and complementary DNA (cDNA) was synthesized with the Takara reverse transcription kit, the Biomake SYBR Green kit, and Thermo PCR equipment, following the manufacturers' instructions. The primer sequences for the target genes were listed in Table 7, with GAPDH serving as the housekeeping gene.

**TABLE 7:** Primer sequences of the osteogenic‐related genes and angiogenic‐related genes, with Gapdh or GAPDH serving as housekeeping gene.

Primer	Sequence
Gapdh‐F	5’‐GGAGAAACCTGCCAAGTATGA‐3’
Gapdh‐R	5’‐TCCTCAGTGTAGCCCAAGA‐3’
Runx2‐F	5’‐ GCCACTTACCACAGAGCTATT‐3’
Runx2‐R	5’‐ GAGGCGATCAGAGAACAAACT‐3’
Bglap2‐F	5’‐ CCAAGCAGGAGGGCAATAA‐3’
Bglap2‐R	5’‐ TCGTCACAAGCAGGGTTAAG‐3’
Spp1‐F	5’‐ GATTCTGTGGACTCGGATGAAT‐3’
Spp1‐R	5’‐GTAGGGACGATTGGAGTGAAAG‐3’
Ctnnb1‐F	5′‐GACACCTCCCAAGTCCTTTATG‐3′
Ctnnb1‐R	5′‐CTGAGCCCTAGTCATTGCATAC‐3′
Lrp5‐F	5′‐GGATGGGCAAGAACCTCTATT‐3′
Lrp5‐R	5′‐GTCAAGGTCTCTCCACACAAG‐3′
GAPDH‐F	5′‐GGTGTGAACCATGAGAAGTATGA‐3′
GAPDH‐R	5′‐GAGTCCTTCCACGATACCAAAG‐3′
HIF1A‐F	5′‐GTCTGCAACATGGAAGGTATTG‐3′
HIF1A‐R	5′‐GCAGGTCATAGGTGGTTTCT‐3′
VEGFA‐F	5′‐ATCAGTTCGAGGAAAGGGAAAG‐3′
VEGFA‐R	5′‐AGGCTCCAGGGCATTAGA‐3′

### 4.5 RNA Transcriptome Sequencing (RNA‐seq) of BMSCs and HUVECs Following Incubation With MABS

To elucidate the biomolecular mechanisms underlying the effects of MABS on the osteogenesis of BMSCs and the angiogenesis of HUVECs, whole‐transcriptome sequencing was performed on the 10BG and PgP groups after culturing BMSCs and HUVECs with MABS extracts for 7 days. Therefore, the experiment employed the TRIzolTM Plus RNA Purification Kit to extract total RNA from BMSCs and HUVECs according to the vendor's protocol. Enrichment of mRNA, fragmentation, reverse transcription, library construction, HiSeq X Ten, and data analysis were performed by Shanghai OE Biotech. Co., Ltd. (Shanghai, China) and Shanghai Personal Biotechnology Co., Ltd. (Shanghai, China). Differential expression analysis was performed using DESeq2. Genes with a q‐value < 0.05 and |log_2_FC| > 0.263 were considered significantly differentially expressed. KEGG pathway enrichment analysis was conducted using the clusterProfiler R package, with significantly enriched pathways defined by an adjusted p‐value (q‐value) < 0.05. Enriched pathways related to osteogenesis and angiogenesis were identified and visualized.

### 4.6 MABS and the In Vivo Performances of Osteogenesis and Angiogenesis

#### 4.6.1 Animal Model Construction and Designation of Experimental Groups

This study utilized Sprague Dawley (SD) rats aged 6‐8 weeks, with an equal representation of male and female participants, each weighing between 200 and 250 grams. The rats were obtained from the Experimental Center of the Second Affiliated Hospital of Harbin Medical University. All animal procedures were approved by the Animal Experiment Committee of the Second Affiliated Hospital of Harbin Medical University and conducted in accordance with the rules set out by the National Institutes of Health (NIH). The SD rats were kept on a normal diet and housed in a regulated environment at a constant temperature of 21°C. Anesthesia was administered through an intraperitoneal injection of sodium pentobarbital at a dosage of 40 mg∙kg^−1^. Subsequently, the cranial region of the rats was shaved and disinfected. A sagittal incision was performed to expose the cranial surface, and under irrigation with PBS, a 5 mm diameter trephine burr was utilized to create precise calvarial defects. Four empirical designations of MABS, namely PgP, 5BG, 10BG, and 20BG, were implanted in rat calvarial defects. Following the suturing of the wound, the experimental rats were administered intramuscular injections of penicillin within 72 h to decrease the danger of infection.

#### 4.6.2 MABS Mediating the In Vivo Osteogenesis Performance

Four weeks following MABS implantation, the experimental rats were killed for analysis. The collected calvarial tissue samples were stored in 10% buffered paraformaldehyde for a maximum of 24 h. The evaluation of new bone formation in calvarial defects was conducted via micro‐CT imaging and histological analysis. Multiple osteogenic parameters of the newly formed tissue were assessed, including the bone volume to total volume (BV/TV) ratio, trabecular number (Tb.N), trabecular separation (Tb.Sp), and trabecular thickness (Tb.Th). Subsequently, collected calvarial tissue samples underwent dehydration and defatting before being embedded in a plastic matrix. A microtome for hard tissue was utilized to prepare undecalcified tissue sections measuring 4 microns in thickness. The sections were then stained with hematoxylin and eosin (H&E) and Masson's trichrome staining. The tissue morphology was evaluated using CaseViewer software under an optical microscope, and the area indicative of relative bone development was assessed with Avizo2019 software (Thermo Fisher Scientific, USA).

The data collected above were then subjected to mean and Gaussian processing. A volumetric model of the point cloud was created via volume reconstruction, incorporating data that included bone and MABS implants. The volumetric model was pseudo‐colored according to the intensity of micro‐CT values, resulting in a pseudo‐colored point cloud volumetric model. Multiple binarization segmentations of tissues with varying densities within the dataset were conducted to generate corresponding 3D reconstruction data, encompassing other tissues, bone tissue, and implants. Representative schematic diagrams were generated from 2D coronal images of micro‐CT analysis corresponding to each empirical designation. Masked binarization data were subsequently employed to independently reconstruct 3D models of bone tissue and implants. Parameters related to osteogenesis (BV/TV, Tb.N, Tb.Th, Tb.Sp) were assessed in calvarial defects and at the interface between bone tissue and the implant. Regions of interest (ROI) were identified to reconstruct pseudo‐colored point cloud models and 3D models of the bone integration area, subsequently enabling a quantitative analysis of the osteogenic parameters.

#### 4.6.3 MABS Mediating the In Vivo Angiogenesis Performance

Four weeks following MABS implantation, the experimental rats were anesthetized through an intraperitoneal injection of sodium pentobarbital at a dosage of 40 mg kg^−^^1^. Then, a high‐resolution small animal ultrasound/photoacoustic imaging system (model VevoLAZR‐X) was utilized to evaluate vascular regeneration surrounding the implants in the cranial defects. Subsequent to the initial data processing, the adjacent tissues were classified into blood vessels and soft tissues. A volumetric model of soft tissues was constructed using point‐cloud data through physical reconstruction, with pseudo‐coloring based on tissue thickness. Binary segmentation was performed to include data related to blood vessels, bone tissue, and implants, resulting in the creation of masked binary datasets. The methodologies for reconstructing bone tissue and scaffolds followed the previously established procedures. The 3D model of the vascular lumen was reconstructed using an inversion function, and the vessel skeletons were generated automatically and pseudo‐colored to indicate lumen diameter. A quantitative morphological analysis was conducted on the reconstructed blood vessels.

### 4.7 Bioadaptive Design of Implants Derived by Dln and the Validation of Matching up to the Osteogenic Regenerative Microenvironment of Bone Defects

This study employs an MLP neural network to create a regression prediction model for evaluating the bioadaptive capacity of implants to the osteogenic microenvironment in bone defects. The initial phase entailed the generation of datasets via finite element simulations, in vitro experiments, and other data sources, requiring the annotation of relationships between composition parameters of MABS and their corresponding outcomes of bone regeneration. Column‐wise random imputation was adopted as the main strategy to process missing data and mitigate data scarcity. Meanwhile, MICE was used for comparative sensitivity assessment, with relevant diagnostic reports produced. The MSE and MAE serve as metrics for quantifying prediction errors. The Adam optimizer was employed to adjust the learning rate dynamically, along with gradient clipping to prevent gradient explosion. The validation set loss was monitored, and early stopping was applied when performance stabilized, thereby preserving optimal model parameters. Prediction accuracy was evaluated through metrics such as the R^2^ score, RMSE, and MAE. The predictive accuracy of the constructed model for bone regeneration of MABS was further assessed in a rabbit tibia defect model (approved by the Ethics Committee, with the approval number YJSDW2023‐054), including Osteogenic gene expression and defect healing rates. Comparative analyses using Random Forest and XGBoost models demonstrate the superior ability of the MLP in handling structured data. We adopted stratified five‐fold cross‐validation for multi‐model comparative analyses to ensure robust and unbiased evaluation. With stratification performed based on BG categories (5, 10, 20 wt.%) in the revised_strict_constrained_model.py workflow, each fold retained a scaffold composition distribution consistent with the overall dataset. Further benchmarking implemented in 8compare.py supplemented an additional stratified 5‐fold cross‐validation strategy stratified by dominant output categories, which systematically compared the performance of Multilayer Perceptron (MLP), Random Forest, and XGBoost models (Table S1). This research identifies the MLP neural network as the core component of the predictive model.

#### 4.7.1 Construction and Training of the Multilayer Perceptron Model

This research involved the design of network architecture, emphasizing both complexity and computational efficiency. The input layer consists of five neurons that represent experimentally derived input features: BG (Percentage of BG), AF, Ani, Ecc, and EqD. The hidden layer comprises five layers, with the number of neurons decreasing sequentially from 512 to 32. The output layer is responsible for producing predictions for three target variables, represented by three neurons: Biocompatibility (Bio), Osteogenic Effect (Osteo), and Angiogenic Effect (Angio). The intermediate hidden layers are designed to capture nonlinear relationships within the dataset. The MSE served as the loss function during the training phase for regression tasks focused on predicting Bio, Osteo, and Angio. An early stopping mechanism was implemented to reduce the risk of overfitting. This mechanism evaluates the model's performance on a validation dataset during the training process. When a decrease in performance on the validation dataset is noted while the training loss persists in its decline—suggesting overfitting—the training process is halted. This method prevents the model from overfitting the training data, enhancing its capacity to generalize to novel, unseen data.

#### 4.7.2 Evaluation and Result Output of the Multilayer Perceptron Model

After the MLP model training is completed, its performance is comprehensively evaluated. Multiple comprehensive regression metrics are calculated to accurately and thoroughly assess the model's predictive capabilities. The MSE represents the average of the squares of the differences between predicted values and actual values. It is expressed in Equation (2):

(2) $$MSE=\frac{1}{n}\sum \frac{n}{i=1}{\left(y_{i}-\hat{y_{i}}\right)}^{2}$$

where *n* is the number of data points, *y_i_* is the actual value, and *ŷ_i_* is the predicted value. MSE provides an overall measure of model error, with lower values indicating better performance. The RMSE is the square root of MSE and shares the same units as the target variable, making this value more interpretable in the context of actual values. The MAE is another important metric, expressed as the average of the absolute differences between predicted values and actual values, as illustrated in Equation (3):

(3) $$MAE=\;\frac{1}{n}\sum \frac{n}{i=1}\left|y_{i}-\hat{y_{i}}\right|$$

Compared with MSE and RMSE, MAE is less sensitive to outliers and provides a more robust measure of average error. The MAPE is expressed as a percentage, represented in Equation (4):

(4) $$\frac{1}{n}\sum \frac{n}{i=1}\frac{\left|y_{i}-\hat{y_{i}}\right|}{y_{i}}\times 100$$

MAPE is of great significance for understanding the relative error of predictions, especially when there are large scale differences in the target variables. The SMAPE is a more symmetric form of MAPE data, which expressed in Equation (5):

(5) $$\frac{200}{n}\sum \frac{n}{i=1}\frac{\left|y_{i}-\hat{y_{i}}\right|}{\left|y_{i}\right|+\left|\hat{y_{i}}\right|}$$

SMAPE addresses some limitations of MAPE, such as the division‐by‐zero issue when *y_i_* = 0. Finally, the coefficient of determination (*R* ^2^) is used to measure how well the model's predictions fit the actual data. An *R* ^2^ value close to 1 indicates a good fit, meaning the model can explain the vast majority of variations in the data. For ease of use and further analysis, the trained MLP model is saved in the universal h5 file format, facilitating model serialization and deserialization. The preprocessed data is stored in tabular files for validation.

#### 4.7.3 Validation of the Adaptation of DLN‐Derived MABS to the Osteogenic Regenerative Microenvironment

To confirm the parameters generated from the multilayer perceptron model, the 4D printed MABS derived from DLN were exposed to iterative updates, and a rabbit tibial defect model was utilized to assess its in vivo optimal adaptation to the osteogenic regenerative microenvironment of bone defect. Herein, New Zealand rabbits, about 8–10‐week‐old, with an equal distribution of males and females, each weighing between 3 and 3.5 kg were involved in this section. All of the rabbits were procured from the Experimental Animal Center of Guangxi Medical University, and surgical procedures were approved by the Animal Experiment Committee of Guangxi Medical University and were conducted in compliance with the rules provided by the National Institutes of Health (NIH). The rabbits were given regular food and maintained in a controlled environment at a temperature of 21°C. A total of Sixteen rabbits (*n* = 4) were randomly assigned to four experimental groups. Following anesthesia via intraperitoneal administration of sodium pentobarbital (40 mg∙kg^−1^), a sagittal incision was done on the posterior surface of the tibia to expose the bone. A tibial defect of 9 mm × 4 mm was produced using a handheld circular saw under the irrigation of PBS. Subsequently, four types of DLN derived MABS—PgP (*n* = 4), 5BG (*n* = 4), 10BG (*n* = 4), and 20BG (*n* = 4)—were implanted into the rabbit tibia defects. During the implantation procedure, the shape‐memory recovery was triggered by irrigating the scaffold with sterile saline pre‐heated to 60°C. To protect the adjacent bone and, more critically, the periosteum and soft tissues from direct and prolonged thermal exposure, these surrounding tissues were temporarily shielded with saline‐soaked gauze during the brief heating period. The incisions were sutured, and penicillin was provided intramuscularly for three consecutive days postoperatively to reduce the risk of infection. 8 weeks following implantation, the rabbits were humanely euthanized via excessive anesthesia administered through the marginal ear vein, and then tibia samples were harvested and preserved in 10% neutral buffered formalin for a maximum duration of 24 h. Osteogenesis and angiogenesis performance of DLN‐derived MABS in rabbit tibia defect was examined using micro‐CT analysis, high‐resolution ultrasound/photoacoustic imaging, and histological examination. The osteogenic parameters of the tibia tissues encompassing BV/TV, Tb.N, Tb.Sp, and Tb.Th, were analyzed from micro‐CT results. Additional criteria analyzed were vessel length, vessel volume, vessel node count, as well as the 3D reconstruction of the medullary cavity model in the bone graft area, which were derived from high‐resolution ultrasound/photoacoustic imaging. Fluid simulation factors such as velocity rendering, pressure rendering, and both quantitative and statistical studies of velocity and pressure were also undertaken.

### 4.8 Statistical Methods

Statistical analyses were performed using IBM SPSS Statistics (version 25.0; IBM Corp., Armonk, NY, USA) and Python Software Foundation. (2021). Python 3.9.9 [Computer software]. All experiments were performed with at least three independent biological replicates. Biological replicates are defined as separate cell passages, separately fabricated scaffolds, or individual animals. Technical replicates were averaged for each biological replicate before statistical analysis. Data are expressed as mean ± SD, and the exact n is indicated in each figure legend and summarized in Table S2. Independent t‐tests or one‐way ANOVA followed by Tukey's post‐hoc test were employed to evaluate intergroup statistical differences in data with homogeneous variance. The Kruskal‐Wallis test, a non‐parametric one‐way ANOVA, was utilized for data exhibiting heterogeneous variance. Statistical significance was defined as *p < 0.05, **p < 0.01, ***p < 0.001, and ****p < 0.0001, whereas ^ns^P > 0.05 was deemed not statistically significant.

## Author Contributions

**Xiongjie Liang**, **Yuechi Zhang**, and **Weifeng Hu** contributed equally to this work and should be considered co‐first authors. **Xiongjie Liang**: Investigation, methodology, experimentation, data curation, writing – review & editing. **Yuechi Zhang**: Investigation, data curation. **Weifeng Hu**: Investigation, data curation, writing – original draft. **Shiyan Lv**: Methodology, validation. **Fan Jia**: Experimentation, investigation, review. **Yan Zhang**: Investigation, review. **Feng Wu**: Investigation, data curation. **Changjiang Yang**: Investigation, data curation. **Guanqi Zhen**: Investigation, review. **Jinglong Yan**: Project administration, funding acquisition, supervision. **Xu Cui**: Formal analysis, writing – review & editing. **Wei Zhao**: Validation, formal analysis, writing – review & editing. **Guanghua Chen**: Supervision, investigation, methodology, data curation, writing – review & editing.

## Conflicts of Interest

The authors declare no conflicts of interest.

## Supporting information




**Supporting File 1**: advs76351‐sup‐0001‐SuppMat.docx.

**Supporting File 2**: advs76351‐sup‐0002‐FigureS1‐S16.zip.

## Data Availability

The data that support the findings of this study are available in the supplementary material of this article.All data are available from the corresponding author on request. Codes for the Multi‐layer perceptron deep learning program is available for reviewers from the corresponding author under request.
