# Supplementary material for: Deep Learning Network‐Tailored Microenvironment Matching of 4D Bioprinting Bioactive Scaffolds for Bone Regeneration
Source: Adv Sci (Weinh). 2026 Jun 30;13(53):e76351. doi: 10.1002/advs.76351 (PMC13336367; doi:10.1002/advs.76351)
Supplement: Supplementary file 1 [file ADVS-13-e76351-s001.docx]

**2.1.2. *Physicochemical Properties of 4D-Printed MABS***

**Supplementary Figure 1.** XRD diffraction patterns of the prepared MABS (**a**), and the corresponding samples after immersion in SBF for 3, 7 and 14 days (**b-e**). The stress-strain curves of MABS with porosity factors of 60% (f) and 20% (g), respectively. Storage modulus (h) and tan delta (i) of MABS with a porosity factor of 60%, as determined using dynamic mechanical thermal analysis (DMTA) (Data are shown as means ± SD, n = 3)

**Supplementary Figure 2.** **a**. SEM and EDS mapping detecting the pore structure and elemental composition of MABS with specific group of BG@PgP. **b**. Representative images showing the influence of scaffolds on culture medium during co-culture.

***2.2. Dataset Construction for Component-Mediated Osteogenic Differentiation of rBMSCs In Vitro***

**Supplementary Figure 3.** **a.** Adhesion of BMSCs on the surface of MABS following incubation period of 24 h, with scale bar 200 μm in upper raw and scale bar 50 μm in bottom raw, arrow indicates cells. **b.** Proliferation of BMSCs and HUVECs following culture with MABS for 24, 48 and 72 h. **c.** ALP staining of BMSCs following culture periods of 5 and 7 days with extracts of MABS (scale bar: 100 μm). **d.** Semi-quantitative analysis of the corresponding ALP staining results in **c**. **e.** Semi-quantitative analysis of ARS staining of BMSCs following culture period of 14 days with extracts from MABS. (Data are presented as means ± SD, n = 3, statistical significance was determined using the one-way ANOVA method with Tukey’s multiple comparisons tests, Statistical significance was defined as *P < 0.05, **P < 0.01, ***P < 0.001, and *****P* < 0.0001, whereas ^ns^P > 0.05 was deemed not statistically significant).

## 2.4.1. Dataset Construction for 4D-Printed Adaptive Bioactive Scaffolds Promoting Osteogenesis in Rat Calvarial Defect Models

**Supplementary Figure 4.** **a.** The quantitative analysis of the migration and vascular formation of HUVECs following 24 h of incubation time with extracts of MABS. **b.** 3D reconstruction of micro-CT analysis on the new bone formation of MABS following an implantation period of 4 weeks in rat calvarial defect model (tra: transparent, sag: sagittal, and 3D reconstruction). Assessment of neovascularization parameters (**c**) and bone formation parameters (**d**), specifically vessels length (VL), vessels volume (VV) and vessels nodes (VN), as well as tubercular thickness (Tb.Th) tubercular space (Tb.Sp) and tubercular number (Tb.N) via micro-CT analysis of bone regeneration in rat calvarial defect model following the implantation of MABS for 4 weeks. (Data are presented as means ± SD, n = 3, statistical significance was determined using the one-way ANOVA method with Tukey’s multiple comparisons tests, Statistical significance was defined as *P < 0.05, **P < 0.01, ***P < 0.001, and *****P* < 0.0001, whereas ^ns^P > 0.05 was deemed not statistically significant). **e.** 3D reconstruction of micro-CT analysis illustrating new bone formation and blood vessel ingrowth in the rat calvarial defect model following implantation for 4 weeks in the PgP- group. **f.** Sagittal and 3D reconstruction of micro-CT showing new bone formation in the rabbit tibial defect model following implantation for 8 weeks in the PgP- group, with an exploded view illustrating the 3D reconstruction of multiple components of newly formed bone, from bottom to top including bone tissue (gray area), combination of soft tissue and scaffold (pink area), and reconstructed blood vessels (network area). The PgP- group in (**b-f**) served as the control group without shape memory programming, demonstrating the positive effects of shape memory on bone integration and angiogenesis in contrast.

**Supplementary Figure 5.** **a.** Vascular recruitment surrounding the subcutaneous MABS following an implantation period of 4 weeks in the rat calvarial defect model, as detected utilizing visual observation (Ectopic) and high-resolution ultrasound/photoacoustic imaging. **b.** Assessment of neovascularization parameters via the corresponding high-resolution ultrasound/photoacoustic imaging in **a.** (Data are presented as means ± SD, n = 3, Statistical significance was determined using the one-way ANOVA method with Tukey’s multiple comparisons tests, Statistical significance was defined as *P < 0.05, whereas ^ns^P > 0.05 was deemed not statistically significant).

## 2.5. Screening the Multilayer Perceptron Model Suitable for Evaluating the Biological Functions of 4D-Printed Adaptive Bioactive Scaffolds

**Supplementary Figure 6.** **a.** Curve illustrating the mean squared error from cross-validation for the random forest model. **b.** Heatmap of feature importance for the XGBoost model; **c.** Distribution of actual and predicted values with regression lines for two models in **a-b. d.** Plot of residuals versus actual values for two models in **a-b. e.** Metrics for stability and interpretability of three models. Bio: biocompatibility; Osteo: osteogenesis; Angio: angiogenesis. (Data are presented as means ± SD, n = 3, statistical significance was determined using the one-way ANOVA method with Tukey’s multiple comparisons tests, Statistical significance was defined as *P < 0.05 and **P < 0.01, whereas ^ns^P > 0.05 was deemed not statistically significant)

**Supplementary Figure. 7.** Forward prediction and overall indicator analysis under strict physical constraints.

**a.** Scatter plots of predicted vs. experimental values for bio-activity, osteogenic, and angiogenic indicators, with R² scores and perfect prediction lines, to evaluate prediction accuracy. **b.** Plots of predicted total vs. true total values for constraint validation, verifying the 100% sum constraint of bio-activity, osteogenic, and angiogenic indicators. **c-d, f.** Three-dimensional distributions, statistical analyses, and data summaries of bio-activity, osteogenic, and angiogenic indicators, for interpreting indicator distributions, validating constraints, and visualizing results. **e.** Histograms of prediction error distributions for bio-activity, osteogenic, and angiogenic indicators, to analyze error distribution characteristics.

**Supplementary Figure 8.** Reverse optimization analysis under strict physical constraints. **a-c.** Visualization of reverse optimization processes and results, including constraint validation, optimization algorithm evaluation, constrained optimization performance, and objective achievement. **d.** Model prediction confidence distribution in reverse optimization. **e.** Constraint error analysis in reverse optimization. **f.** Chart of constraint satisfaction rates across functional modules for verifying constraint validity, demonstrating overall physical constraint performance.

**Supplementary Figure 9.** Virtual experiment analysis under strict physical constraints **a.** Performance comparisons among different model versions, illustrating the model evolution process. **b.** Error convergence trajectory during reverse optimization, demonstrating optimization algorithm performance. **c.** Heatmap visualization of model performance metrics for comprehensive performance evaluation. **d.** Scatter plot of prediction accuracy in virtual experiments, validating model generalization ability. **e.** Constraint validation in virtual experiments, verifying constraint maintenance during generalization. **f.** Prediction error distribution in virtual experiments, analyzing generalization error characteristics.

## 2.5.2. Validation of the Effectiveness of Multilayer Perceptron Model for Evaluating the Biological Functionality of Composite Materials

**Supplementary Figure 10.** Fabrication of 4D-printed MABS utilizing parameters optimized by a MLP deep learning model, followed by implantation into a rabbit tibial defect after shape pre-programming. **a.** The permanent morphology of MABS at ambient temperature. **b.** Temporary shape distortion of MABS elicited at 60 °C. **c.** Shape retention capacity of MABS upon reversion to ambient temperature. **d.** The implantation of the malformed MABS and its ensuing shape recovery within the rabbit tibial defect upon exposure to sterile saline at 60 °C. **e.** The tibial deficiency is completely filled post-implantation, offering strong mechanical support and stability without the need for additional plate attachment, and exhibiting adaptive responsiveness to the conditions inside and around the defect area. **f.** Wound closure subsequent to the fixation of the 4D-printed MABS implant.

**Supplementary Figure 11.** **a.** Sagittal and 3D reconstruction of micro-CT analysis on the new bone formation of MABS following an implantation period of 8 weeks in the rabbit tibial defect model, CP, SP, and HP illustrating the coronal plane, sagittal plane, and horizontal plane of micro-CT respectively, whereas ROI indicating the region of interest, and IC meaning the intensity classification. **b.** Assessment of neovascularization parameters (**b**) and bone formation parameters(**c**) , specifically bone volume/tissue volume (BV/TV), tubercular thickness (Tb.Th) tubercular space (Tb.Sp) and tubercular number (Tb.N), as well as vessels length (VL), vessels volume (VV) and vessels nodes (VN) via Micro-CT analysis of bone regeneration in rabbit tibial defect model following the implantation of MABS for 8 weeks. (Data are presented as means ± SD, n = 4 (pls confirm the replicate number), statistical significance was determined using the one-way ANOVA method with Tukey’s multiple comparisons tests, Statistical significance was defined as *P < 0.05, **P < 0.01, ***P < 0.001, and *****P* < 0.0001, whereas ^ns^P > 0.05 was deemed not statistically significant).

**Supplementary Figure 12.** Shape fixity ratio (Rf) and shape recovery ratio (Rr) during five shape memory cycles. **a.** 5BG, **b.** 10BG, **c.** 20BG.

**Supplementary Figure 13.** **a.** The XRD pattern of BSG shows a broad diffuse halo peak around 2θ = 30°, with no sharp crystalline diffraction peaks observed. **b.** The FT-IR results indicate that the bioactive glass exhibits a typical glass network structure. The broad absorption band at 3431 cm⁻¹ is assigned to the O–H stretching vibration of surface hydroxyl groups and adsorbed water, indicating the presence of abundant active hydroxyl groups on the glass surface.

**Supplementary Figure 14.** Western blotting analysis of cell lysates cultured under different scaffold extract conditions. **a, b.** BMSCs co-cultured under extract conditions for 7 days. **c.** HUVECs cultured under extract conditions for 48 h.

**Supplementary Figure 15.** Overview of the constrained deep learning multi-objective prediction workflow. Raw data (23 features, 3 targets) undergo preprocessing (missing value imputation, stratified train/val/test split: 70/15/15). A constrained neural network (architecture: 23→512→256→128→64→32→3, Softmax output with Σ=1.0, 5-fold CV, Z-score normalization) is trained on the train/val sets. The saved model powers five downstream modules: forward prediction, reverse optimization, virtual experimentation, SHAP analysis, and constraint iteration analysis. Outputs from predictive modules are visualized (scatter plots, heatmaps, etc.), while interpretability and constraint analyses feed into a benchmark comparison against XGBoost, random forest, and standard MLP models.

**Supplementary Figure 16.** Detailed data preprocessing pipeline with constraint preservation. Raw data (N samples × 23 features) are processed in two parallel streams:(1) Feature processing: Missing values are imputed via random sampling (low missingness, <5%) or MICE (Iterative Imputer, max_iter=10, seed=42), followed by Z-score normalization (fit on training set only). Target processing: Three composite scores (Biocompatibility, Osteogenic, Angiogenic) are derived from predefined indicator groups, then normalized via stabilized Softmax to enforce Σ(scores)=1.0. (2) Data are split (0.7/0.15/0.15) and loaded into PyTorch DataLoaders (batch size=32) as normalized tensors ([B,23] features, [B,3] targets). The pipeline ensures the compositional constraint is preserved through imputation, target normalization, and model architecture.

**Supplementary Table 1.** 5-Fold Cross-Validation Performance Summary of Models

| **model** | **fold** | **MSE** | **R2** | **RMSE** | **MAE** |
| --- | --- | --- | --- | --- | --- |
| XGBoost | 1 | 0.00014 | 0.94144 | 0.01168 | 0.00765 |
| XGBoost | 2 | 0.00017 | 0.93217 | 0.01304 | 0.00787 |
| XGBoost | 3 | 0.00011 | 0.95582 | 0.01030 | 0.00713 |
| XGBoost | 4 | 0.00014 | 0.93547 | 0.01192 | 0.00743 |
| XGBoost | 5 | 0.00010 | 0.95731 | 0.01017 | 0.00699 |
| RandomForest | 1 | 0.00022 | 0.91196 | 0.01478 | 0.01051 |
| RandomForest | 2 | 0.00024 | 0.90670 | 0.01544 | 0.01057 |
| RandomForest | 3 | 0.00016 | 0.92958 | 0.01264 | 0.00950 |
| RandomForest | 4 | 0.00021 | 0.90819 | 0.01444 | 0.01014 |
| RandomForest | 5 | 0.00016 | 0.93084 | 0.01280 | 0.00961 |
| MLP | 1 | 0.00003 | 0.98953 | 0.00545 | 0.00313 |
| MLP | 2 | 0.00003 | 0.98650 | 0.00583 | 0.00347 |
| MLP | 3 | 0.00001 | 0.99438 | 0.00372 | 0.00250 |
| MLP | 4 | 0.00001 | 0.99453 | 0.00361 | 0.00245 |
| MLP | 5 | 0.00002 | 0.99322 | 0.00422 | 0.00260 |

**Supplementary Table 2.** Summary of sample sizes for major experiments

| **Experiment / Assay** | **Biological replicates**  **(n)** | **Technical replicates per biological replicate** | **Notes** |
| --- | --- | --- | --- |
| In vitro | - | - | - |
| CCK‑8 proliferation (rBMSCs & HUVECs) | 3 (independent cell passages) | 6 wells | Data represent mean of 6 technical replicates per biological replicate $\text{n}\text{=3}$ |
| Live/dead staining | 3 (independent cell passages) | 3 fields of view | Representative images from each replicate; quantification across 3 fields |
| ALP staining (qualitative & quantitative) | 3 (independent cell passages) | 3 wells | Absorbance measured in triplicate wells per biological replicate |
| Alizarin Red S (ARS) staining | 3 (independent cell passages) | 3 wells | As above |
| Scratch assay (HUVEC migration) | 3 (independent cell passages) | 3 replicate scratches per experiment | Healing rate quantified from 3 fields per scratch |
| Tube formation assay | 3 (independent cell passages) | 3 wells | Microvessel length, volume, and loop count analyzed via ImageJ |
| RT‑qPCR (osteogenic/angiogenic genes) | 3 (independent cell passages) | 3 technical replicates per gene | Each sample run in triplicate; data normalized to GAPDH |
| RNA‑seq (transcriptome) | 3 (independent cell passages) | N/A | Performed on pooled RNA from 3 wells per biological replicate |
| ICP‑AES / pH measurement | 3 (independently fabricated scaffolds) | 3 measurements per scaffold | Scaffolds from separate fabrication batches |
| Mechanical testing (compression) | 5 (independently fabricated scaffolds) | 1 test per scaffold | Outliers removed; n=5 as stated in manuscript |
| In vivo – rat calvarial defect | - | - | - |
| Micro‑CT analysis (bone parameters) | 3 animals per group | N/A | Each animal considered one biological replicate; 3D reconstruction and trabecular parameters quantified |
| Histology (H&E, Masson) | 3 animals per group | 3 sections per animal | Representative images shown |
| Ultrasound/photoacoustic angiography | 3 animals per group | 2 independent scans per animal | Vessel volume, length, and branching quantified |
| In vivo – rabbit tibial defect | - | - | - |
| Micro‑CT & 3D reconstruction | 4 animals per group | N/A | As described in Methods: n=4 per group (total 16 rabbits) |
| Finite element/fluid simulation | 4 animals per group | 3 independent simulations per model | Pressure and velocity rendering from CT data |
| Deep learning model | - | - | - |
| Training dataset | 1794 samples | N/A | Derived from 2,564 experimental samples 70% (see Table 2 in manuscript) |
| Validation dataset | 385 samples | N/A | Independent validation set 15% |
| Test dataset | 385 samples | N/A | Independent test set 15% |
| Virtual experiment validation | 50 virtual samples | N/A | Generated from test set distribution |

**Discussion**

The MLP model developed in this study demonstrated exceptional predictive accuracy (overall R² = 0.944), outperforming previous machine learning models for BSG-based scaffold design (e.g., R² = 0.85–0.90 reported by Liu et al. ^[1]^. The integration of a Softmax output layer and constraint loss function ensured 100% satisfaction of the biological feasibility constraint (bio + osteo + angio = 100%), addressing a critical limitation of unconstrained models where predictions may violate physiological plausibility^[2]^. This strict constraint mechanism enhances the scientific rigor of the model^[3]^.

The higher R² score for biocompatibility (0.983) compared to angiogenesis (0.893) reflects the greater sensitivity of biocompatibility to BSG concentration (average sensitivity = 0.1394 vs. 0.0659 for angiogenesis)^[3]^. This finding is consistent with previous studies indicating that BSG concentration is a dominant factor regulating cell proliferation and adhesion^[1]^. The moderate prediction error for angiogenesis (MAPE = 9.69%) may be attributed to the higher complexity of angiogenic pathways, which involve more biological indicators (10 vs. 4 for biocompatibility) and stronger non-linear interactions.

Reverse optimization using the BFGS algorithm successfully identified optimal BSG parameters for target biological performance, with a minimum total error of 0.5578 for the high biocompatibility scenario. This capability provides a scientific basis for the rational design of 4D scaffolds, reducing the need for time-consuming and costly trial-and-error experiments^[4]^. For example, the optimal BSG concentration (11.62) identified for high biocompatibility falls within the range (10–15) previously reported to balance bioactivity and mechanical stability^[1]^. The consistency of optimal Ani (0.560) and Ecc (0.732) values across target combinations suggests that these parameters play a synergistic role in regulating multiple biological functions, a finding supported by correlation analysis showing a strong positive correlation between Ani and Ecc (r = 0.990).

The incremental learning module addressed a key challenge in material science AI models: adapting to new experimental data without retraining the entire model^[5]^. By using experience replay with a memory bank of 500 samples, the model retained historical knowledge while incorporating new information, resulting in a 3.6% performance improvement. This feature is particularly valuable for scaffold development, where experimental data are often accumulated incrementally. The web application further enhances accessibility, enabling researchers and engineers to utilize the model without specialized programming skills—aligning with the trend of user-friendly AI tools in material science.

Despite its strengths, this design has several limitations. First, the model’s prediction range is constrained by the training data (e.g., BSG concentration: 5–20), limiting its applicability to extreme parameter values^[6]^. Second, the black-box nature of deep learning reduces model interpretability, which could be improved by integrating explainable AI (XAI) techniques such as SHAP or LIM. Third, virtual experiment validation, while statistically robust, requires further confirmation with in vitro and in vivo experiments to validate biological relevance. Future work will focus on three directions: (1) expanding the dataset to include more diverse BSG compositions and biological environments to improve generalization; (2) integrating multi-modal data (e.g., micro-CT images, mechanical testing data) to enhance prediction accuracy; and (3) developing a cloud-based deployment to support real-time collaboration and large-scale data processing. Additionally, optimizing the reverse optimization algorithm with multi-objective functions (e.g., incorporating cost and manufacturing feasibility) will further enhance industrial applicability^[4]^.

To enhance research transparency and reproducibility, we have fully refactored all computational codes and released a public GitHub repository (https://github.com/GuanghuaChenHMU/ProjectMABS). This open-source resource integrates standardized datasets, complete processing pipelines, trained models, and configuration files, enabling full execution and extension of our biomedical material prediction workflow. The repository contains 70%/15%/15% partitioned training, validation and test datasets, alongside a full suite of executable scripts. These cover data imputation, five-fold cross-validated constrained neural network training, hybrid optimization-based reverse prediction, virtual experiment sensitivity analysis, SHAP interpretability evaluation, constraint benchmarking, scientific visualization, and multi-model comparative analysis. Key experimental assets, including trained model weights, normalization statistics, hyperparameter configurations and training logs, are fully preserved. This repository allows researchers to reproduce all experimental results and adapt the framework for novel biomaterial research. We have updated the data availability statement to endorse this open-science practice, strengthening the credibility and generalizability of this work.

**References**

[1]	L. Li, Y. Huang, J. Qin, J. R. Honiball, D. Wen, X. Xie, Z. Shi, X. Cui, B. Li, *Biomater. Adv.* **2022**, *138*, 212949.

[2]	W. Liu, Y. Zhang, Y. Lyu, S. Bosiakov, Y. Liu, *Front. Bioeng. Biotechnol.* **2023**, *11*, 1241151.

[3]	Y. Liu, Q. Cao, S. Yong, J. Wang, X. Chen, Y. Xiao, J. Lin, M. Yang, K. Wang, X. Li, X. Zhu, X. Zhang, *Biomaterials* **2025**, *321*, 123348.

[4]	W. Liu, Y. Zhang, Y. Lyu, S. Bosiakov, Y. Liu, *Front. Bioeng. Biotechnol.* **2023**, *11*, 1241151.

[5]	“Interpretable AI in Tissue Engineering: XGBoost and SHAP for PLGA Scaffold Biocompatibility,” DOI 10.1101/2024.11.21.624734can be found under https://www.semanticscholar.org/paper/Interpretable-AI-in-Tissue-Engineering%3A-XGBoost-and-Rafat/779a7364d37110c1f692e06a933b3f1377f0ac2e, **2024**.

[6]	F. Ge, C. Li, S. Iqbal, A. Muhammad, F. Li, M. A. Thafar, Z. Yan, A. Worachartcheewan, X. Xu, J. Song, D.-J. Yu, *Brief. Bioinform.* **2023**, *24*, bbac535.
