# Supplementary figures and images for: Deep Learning Network‐Tailored Microenvironment Matching of 4D Bioprinting Bioactive Scaffolds for Bone Regeneration
Source: Adv Sci (Weinh). 2026 Jun 30:e76351. Online ahead of print. doi: 10.1002/advs.76351 (PMC13336367; doi:10.1002/advs.76351)

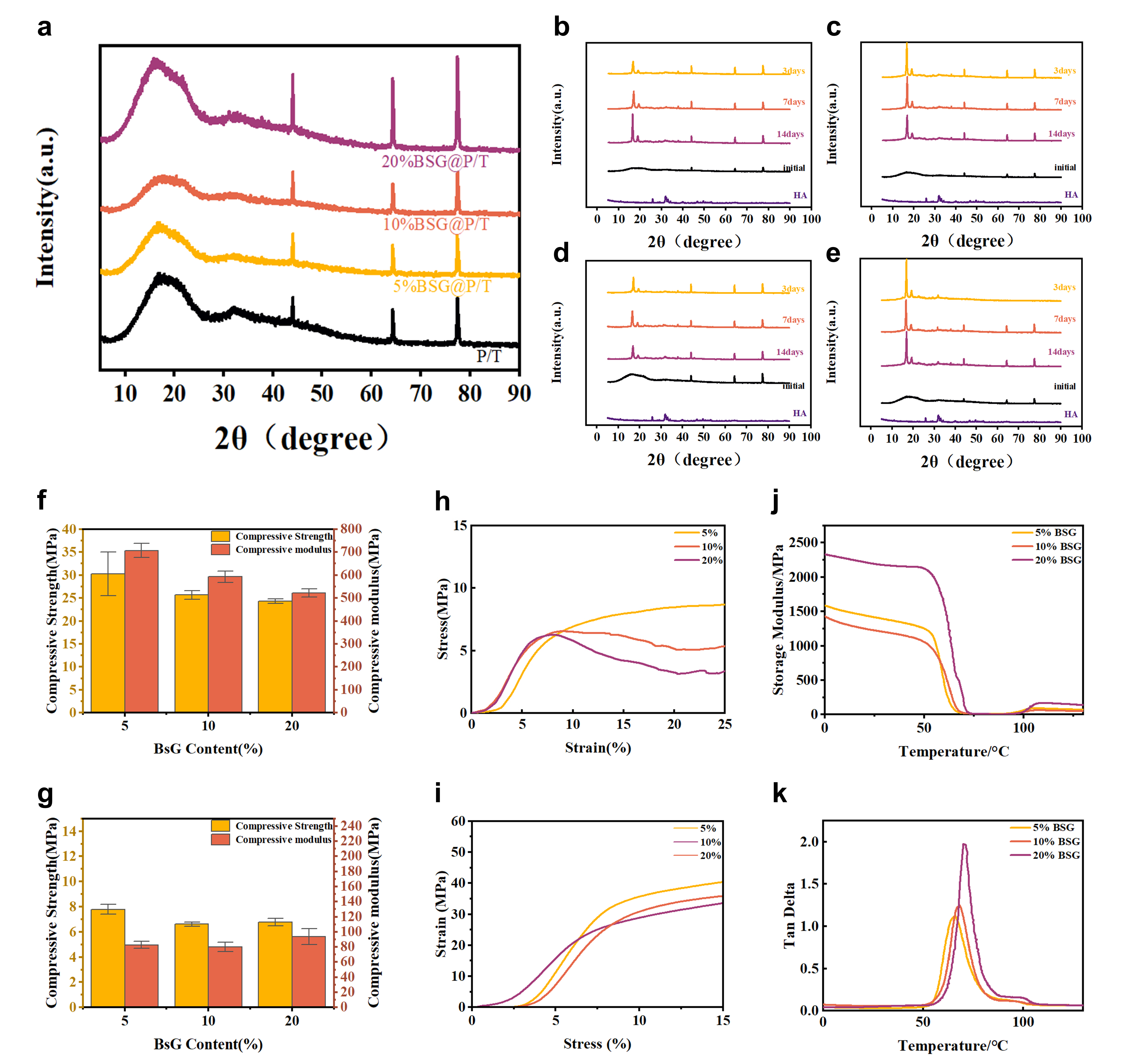

Supplement: Supplementary file 2 — Supporting File 2: advs76351‐sup‐0002‐FigureS1‐S16.zip. [file ADVS-9999-e76351-s001.zip › Fig S1.png]

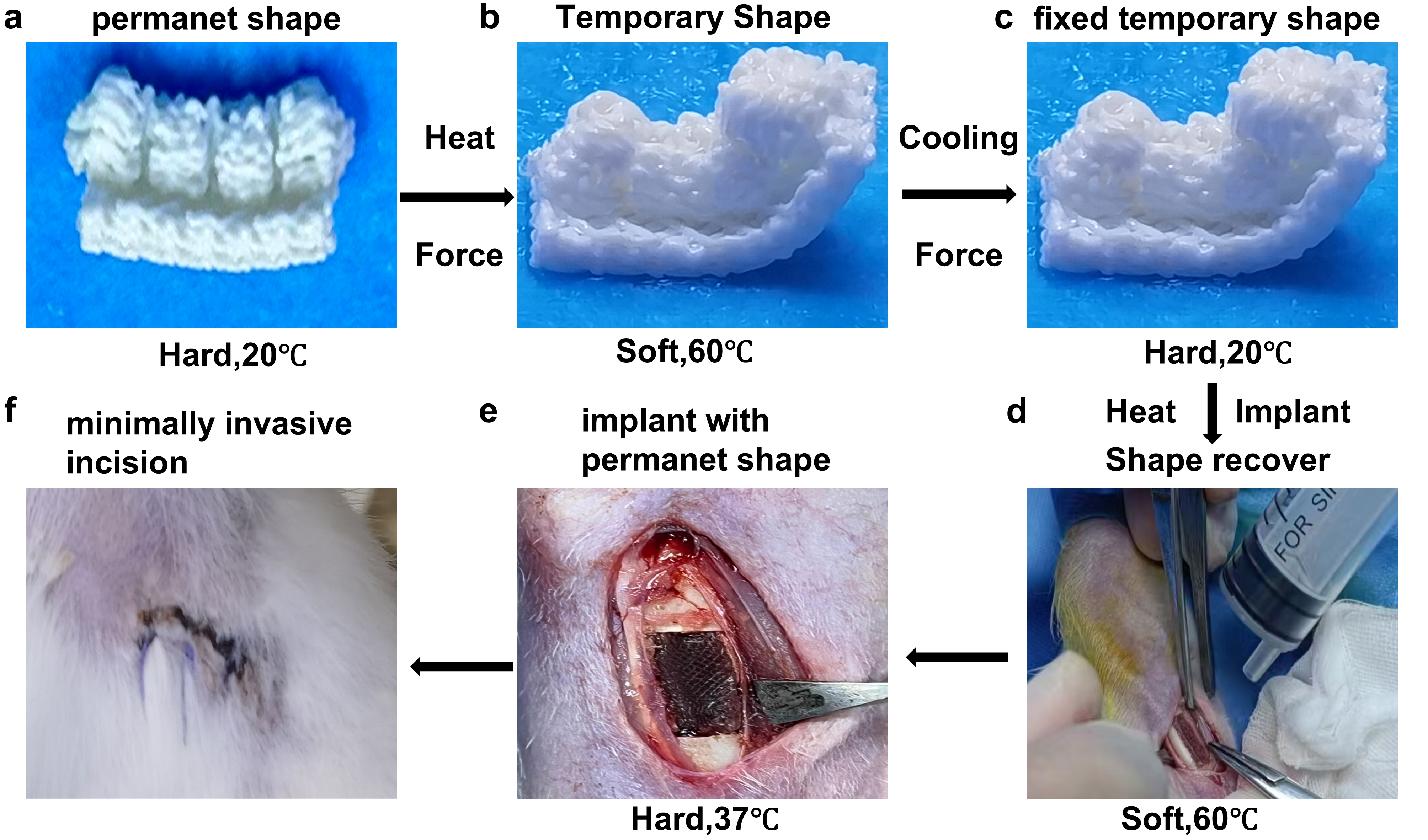

Supplement: Supplementary file 2 — Supporting File 2: advs76351‐sup‐0002‐FigureS1‐S16.zip. [file ADVS-9999-e76351-s001.zip › Fig S10.png]

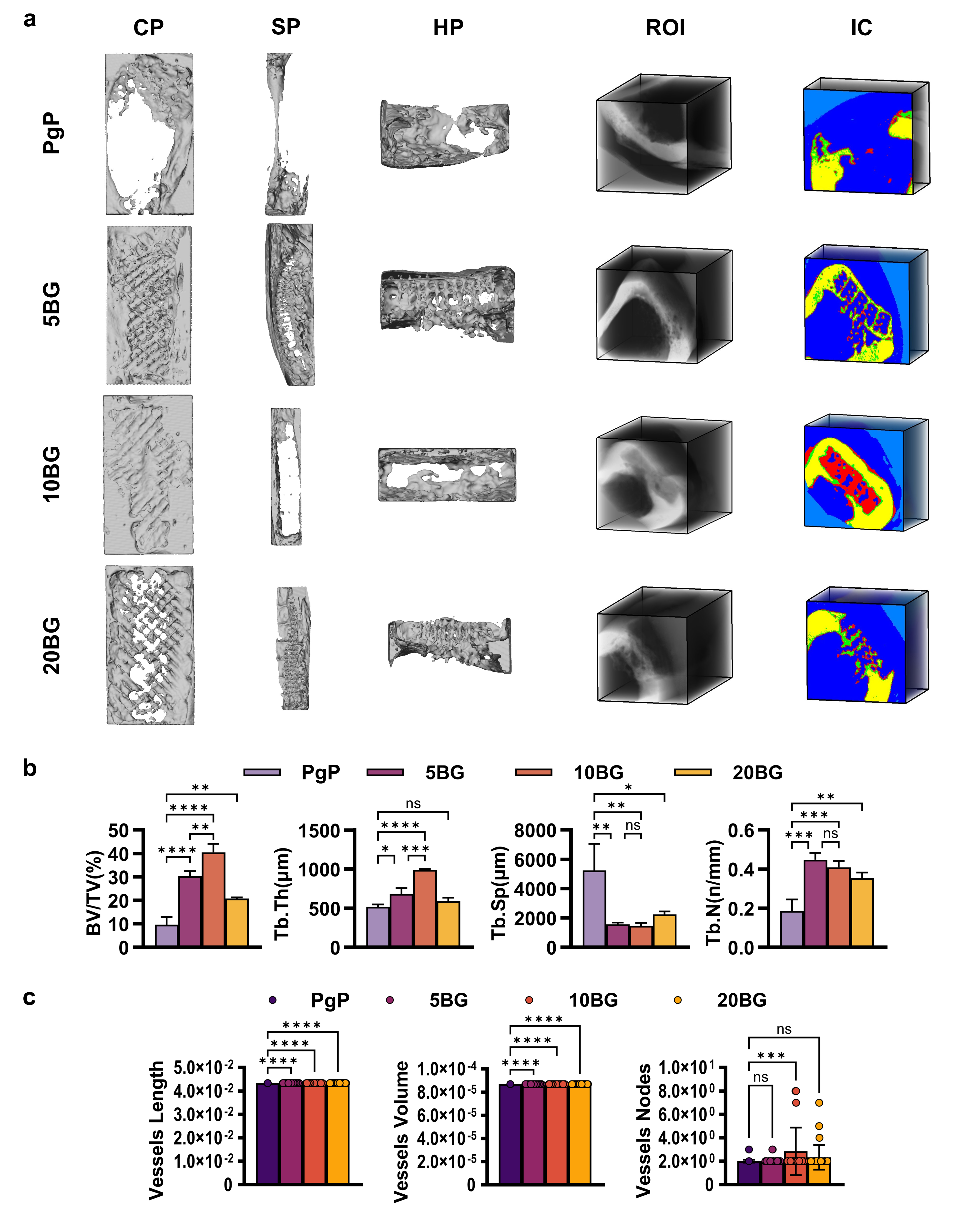

Supplement: Supplementary file 2 — Supporting File 2: advs76351‐sup‐0002‐FigureS1‐S16.zip. [file ADVS-9999-e76351-s001.zip › Fig S11.png]

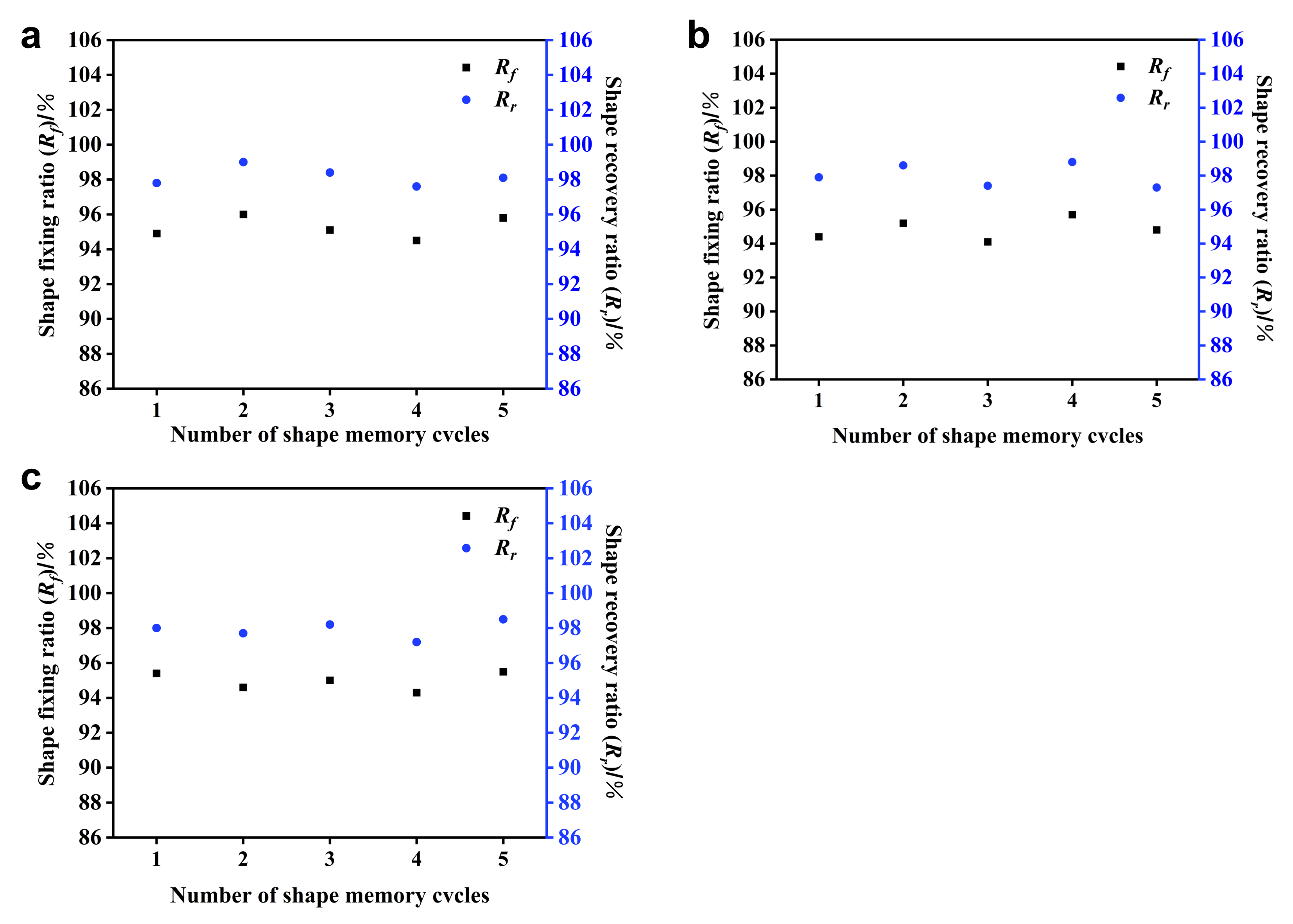

Supplement: Supplementary file 2 — Supporting File 2: advs76351‐sup‐0002‐FigureS1‐S16.zip. [file ADVS-9999-e76351-s001.zip › Fig S12.png]

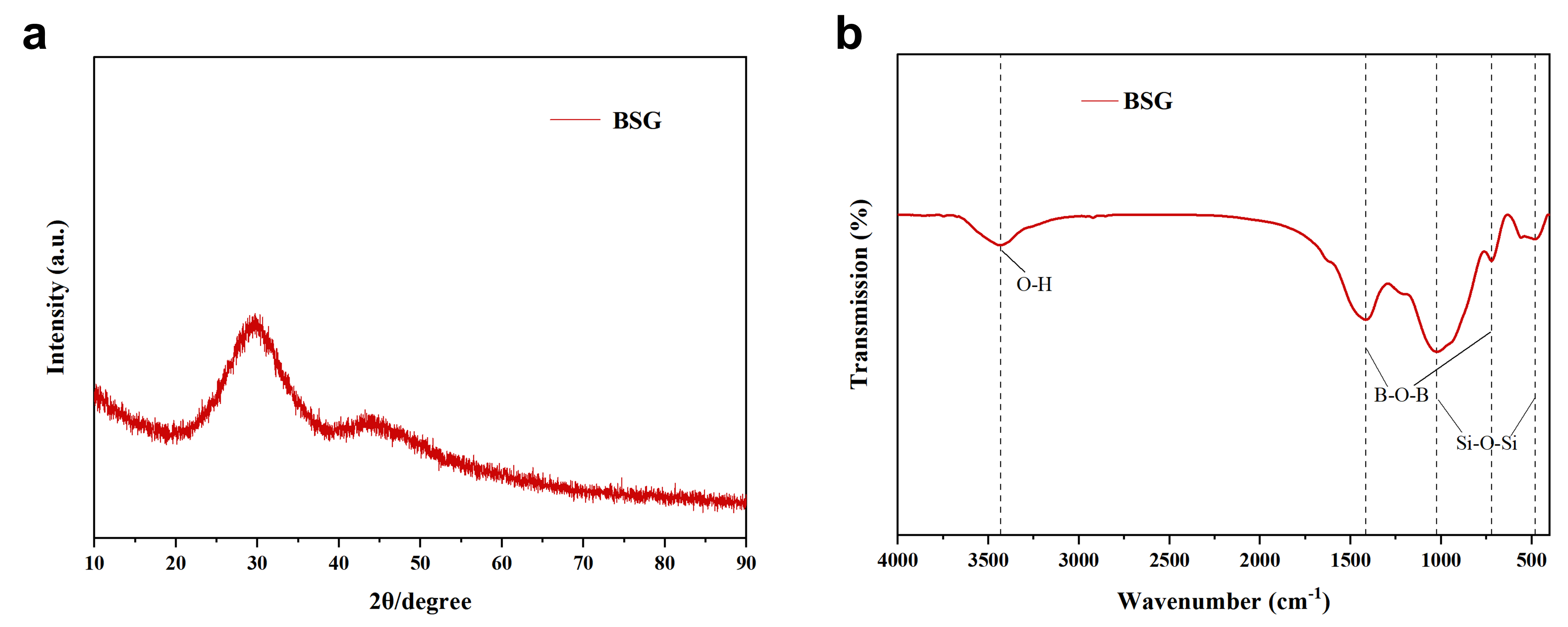

Supplement: Supplementary file 2 — Supporting File 2: advs76351‐sup‐0002‐FigureS1‐S16.zip. [file ADVS-9999-e76351-s001.zip › Fig S13.png]

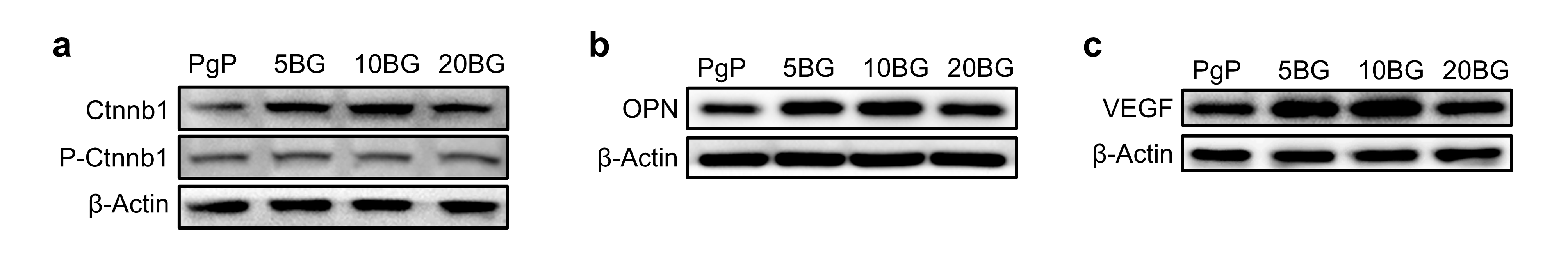

Supplement: Supplementary file 2 — Supporting File 2: advs76351‐sup‐0002‐FigureS1‐S16.zip. [file ADVS-9999-e76351-s001.zip › Fig S14.png]

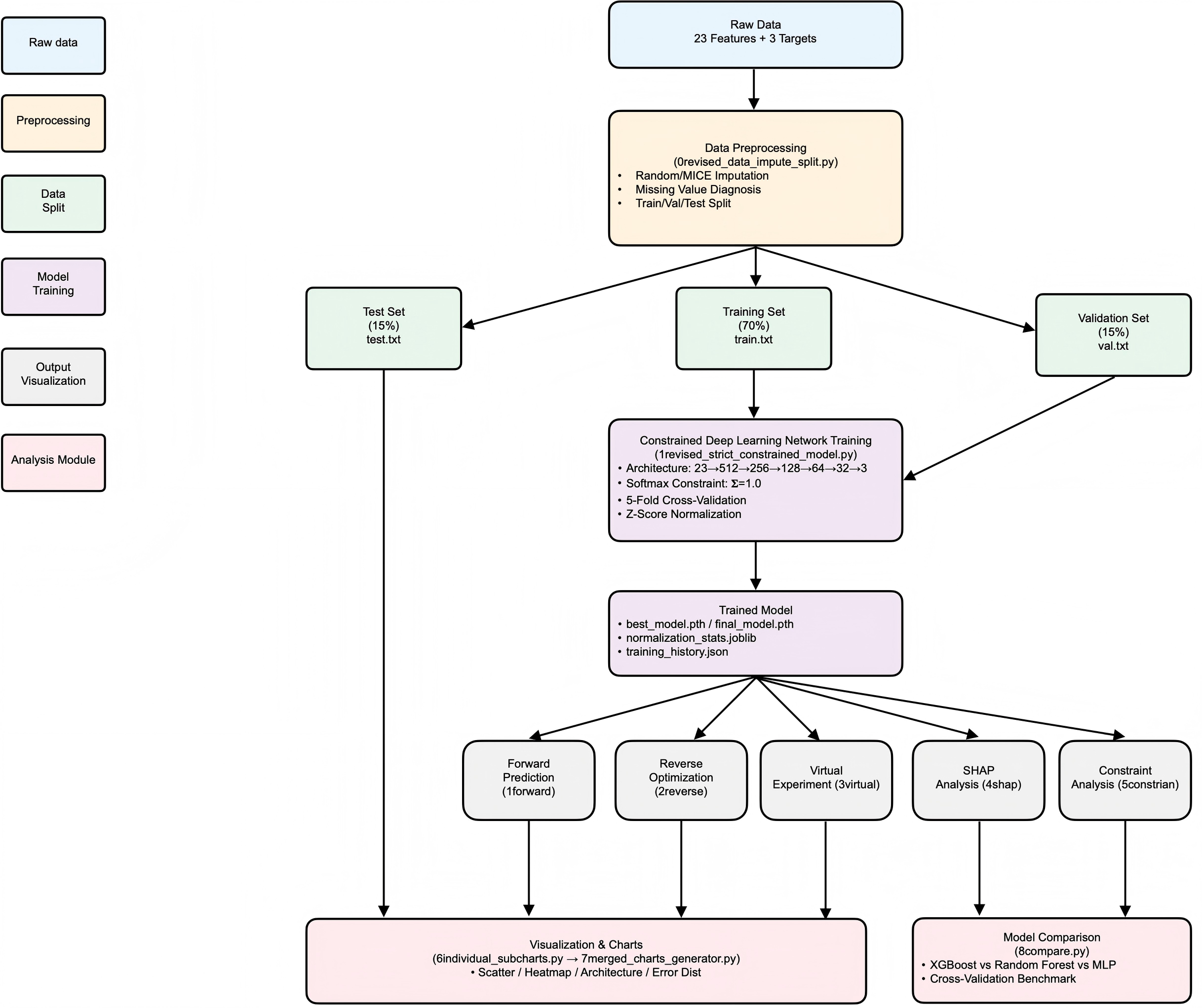

Supplement: Supplementary file 2 — Supporting File 2: advs76351‐sup‐0002‐FigureS1‐S16.zip. [file ADVS-9999-e76351-s001.zip › Fig S15.png]

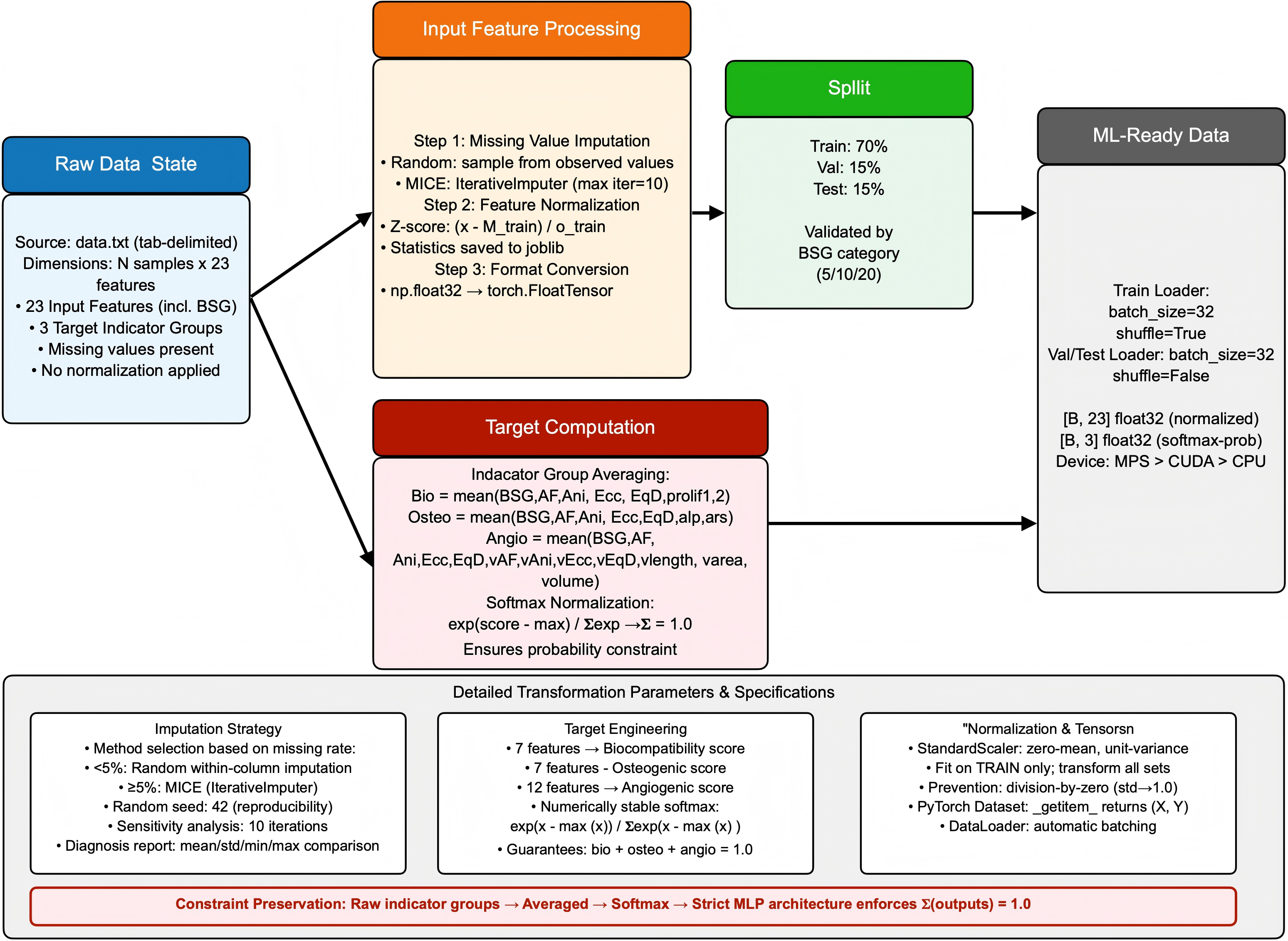

Supplement: Supplementary file 2 — Supporting File 2: advs76351‐sup‐0002‐FigureS1‐S16.zip. [file ADVS-9999-e76351-s001.zip › Fig S16.png]

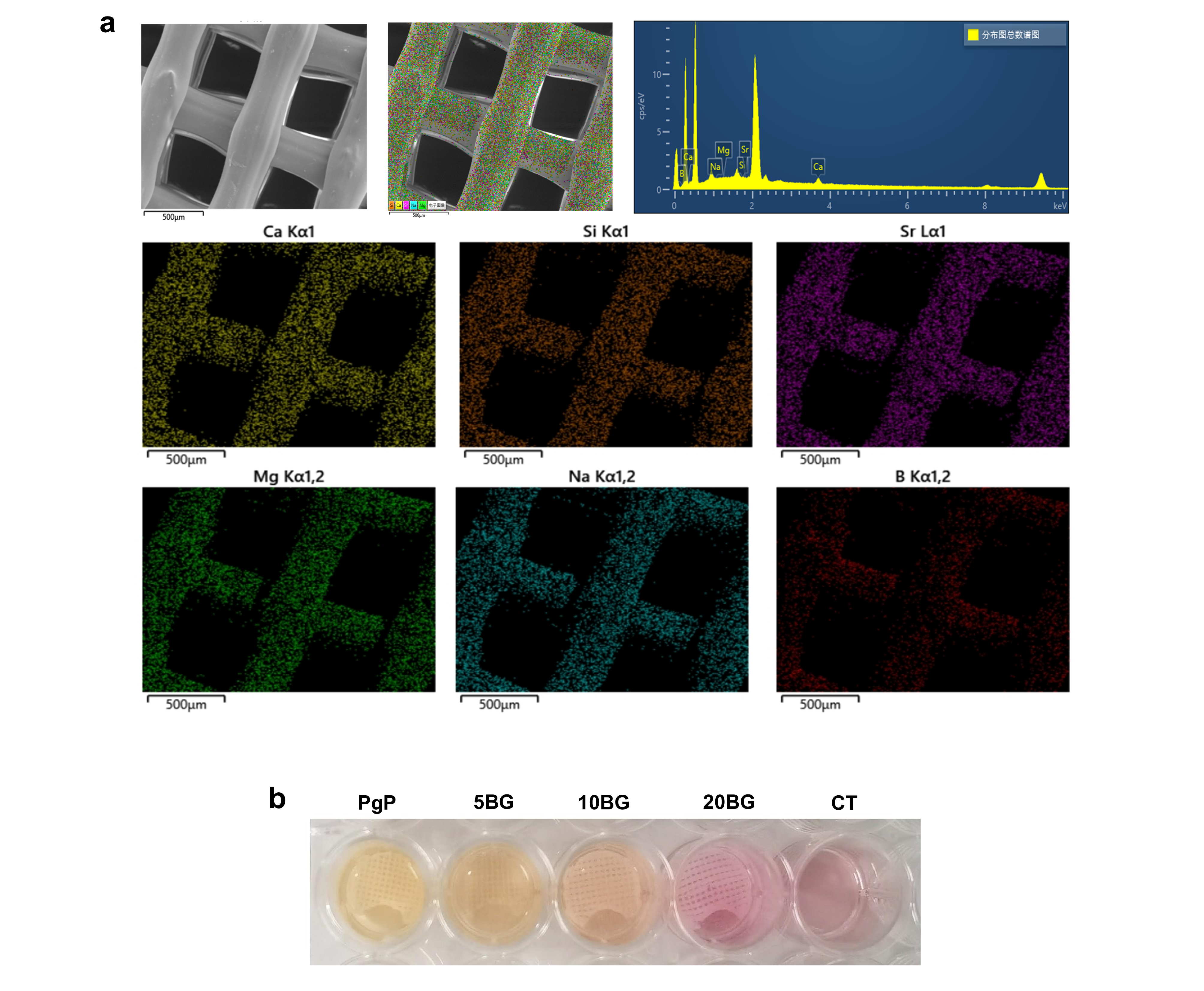

Supplement: Supplementary file 2 — Supporting File 2: advs76351‐sup‐0002‐FigureS1‐S16.zip. [file ADVS-9999-e76351-s001.zip › Fig S2.png]

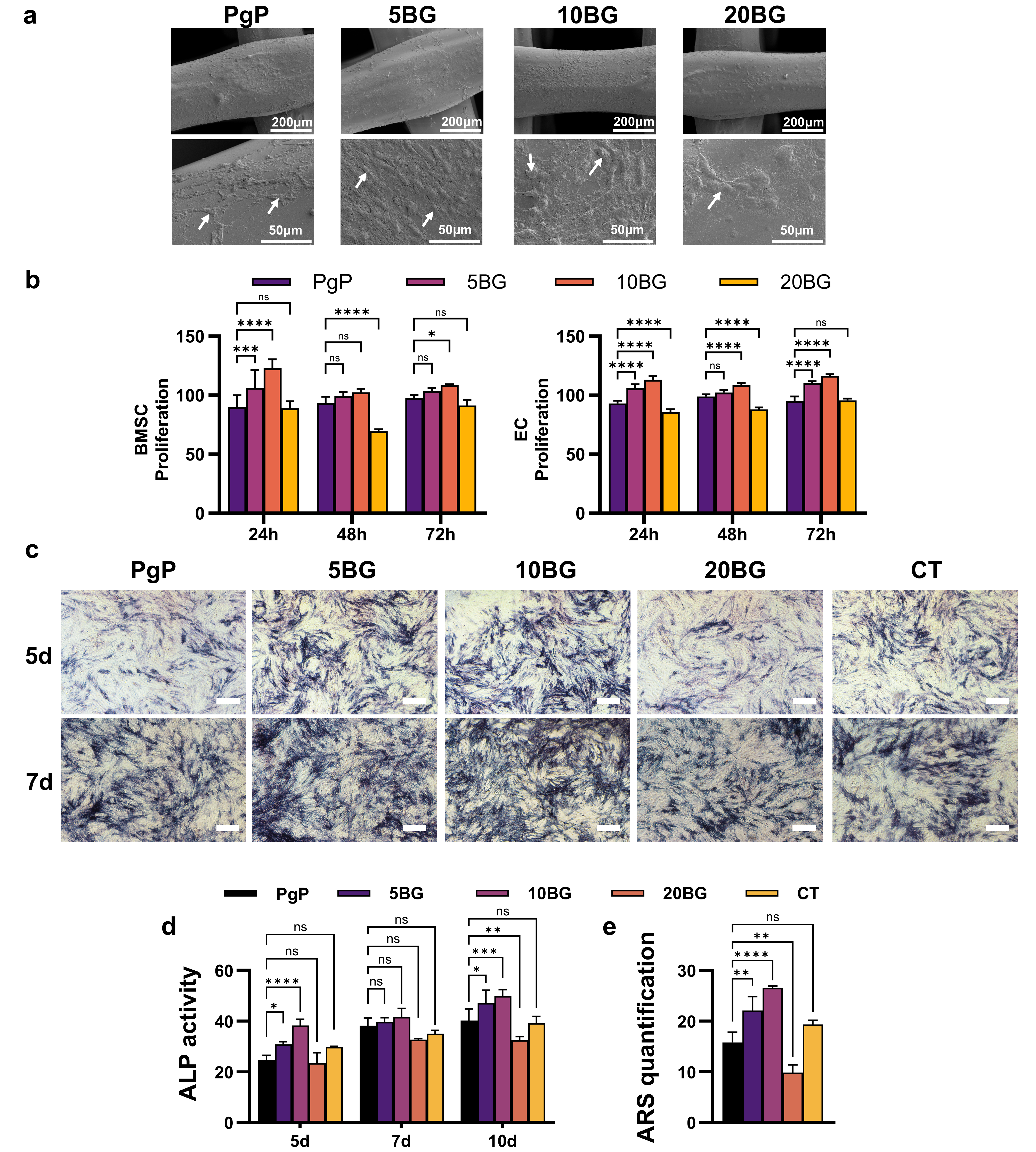

Supplement: Supplementary file 2 — Supporting File 2: advs76351‐sup‐0002‐FigureS1‐S16.zip. [file ADVS-9999-e76351-s001.zip › Fig S3.png]

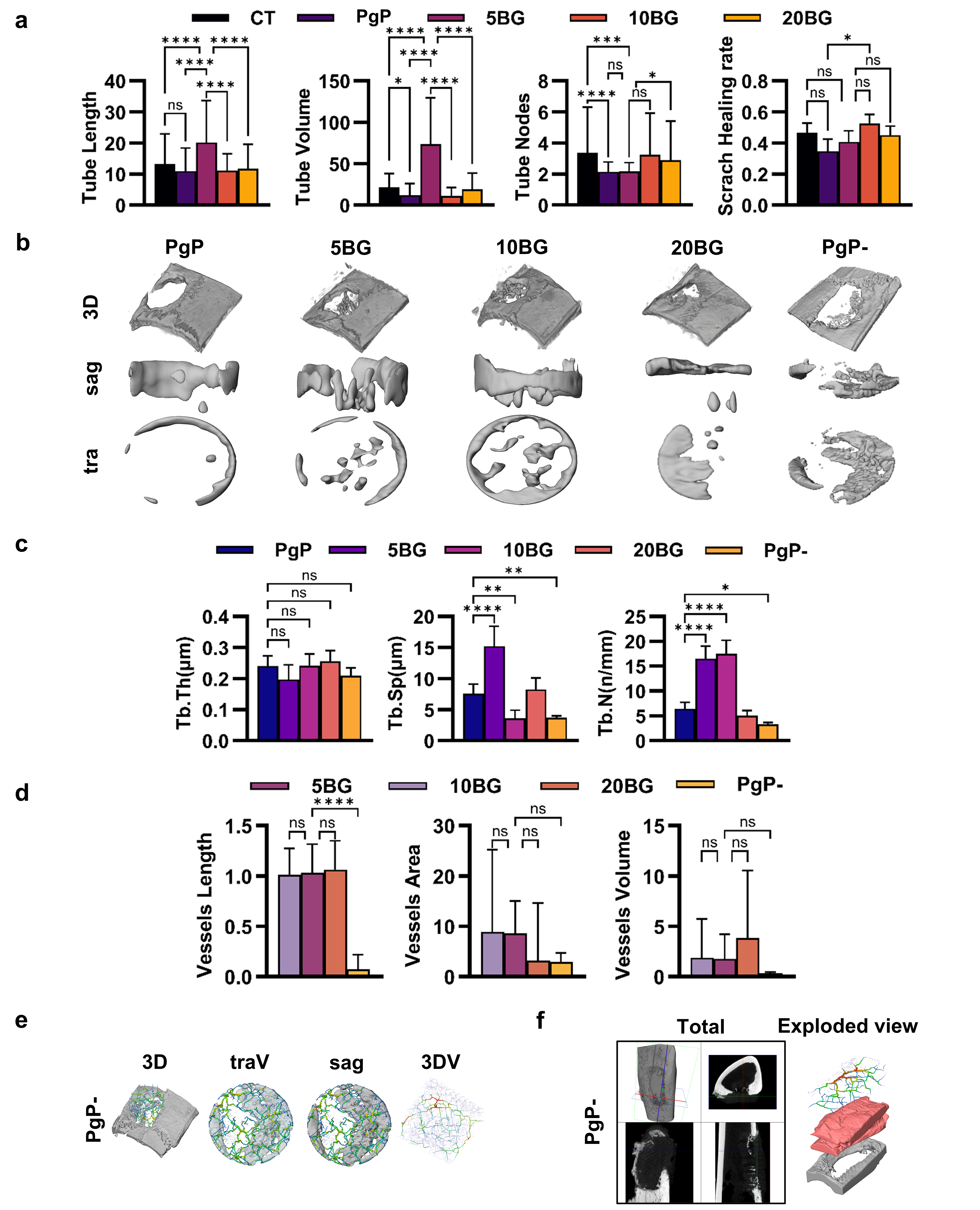

Supplement: Supplementary file 2 — Supporting File 2: advs76351‐sup‐0002‐FigureS1‐S16.zip. [file ADVS-9999-e76351-s001.zip › Fig S4.png]

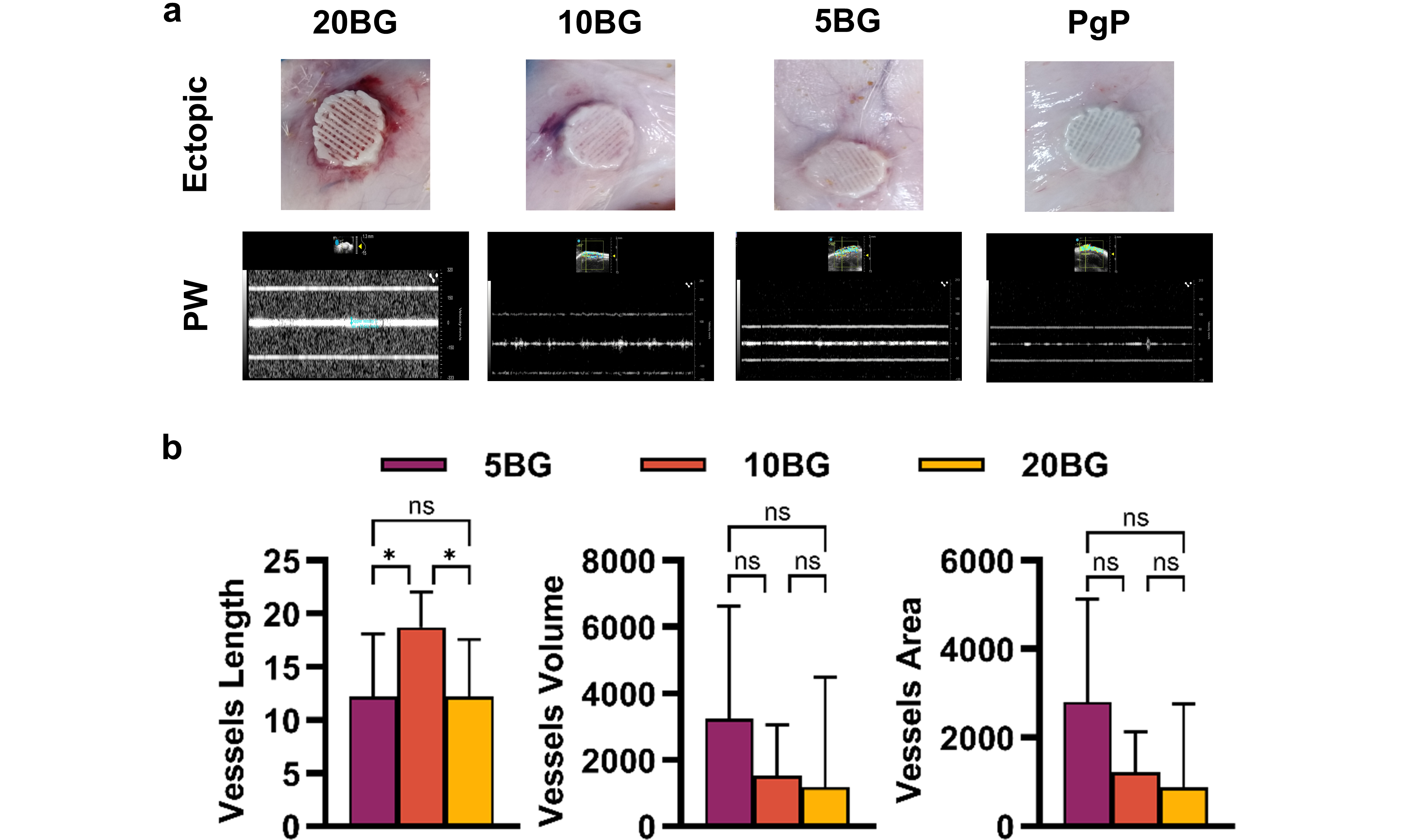

Supplement: Supplementary file 2 — Supporting File 2: advs76351‐sup‐0002‐FigureS1‐S16.zip. [file ADVS-9999-e76351-s001.zip › Fig S5.png]

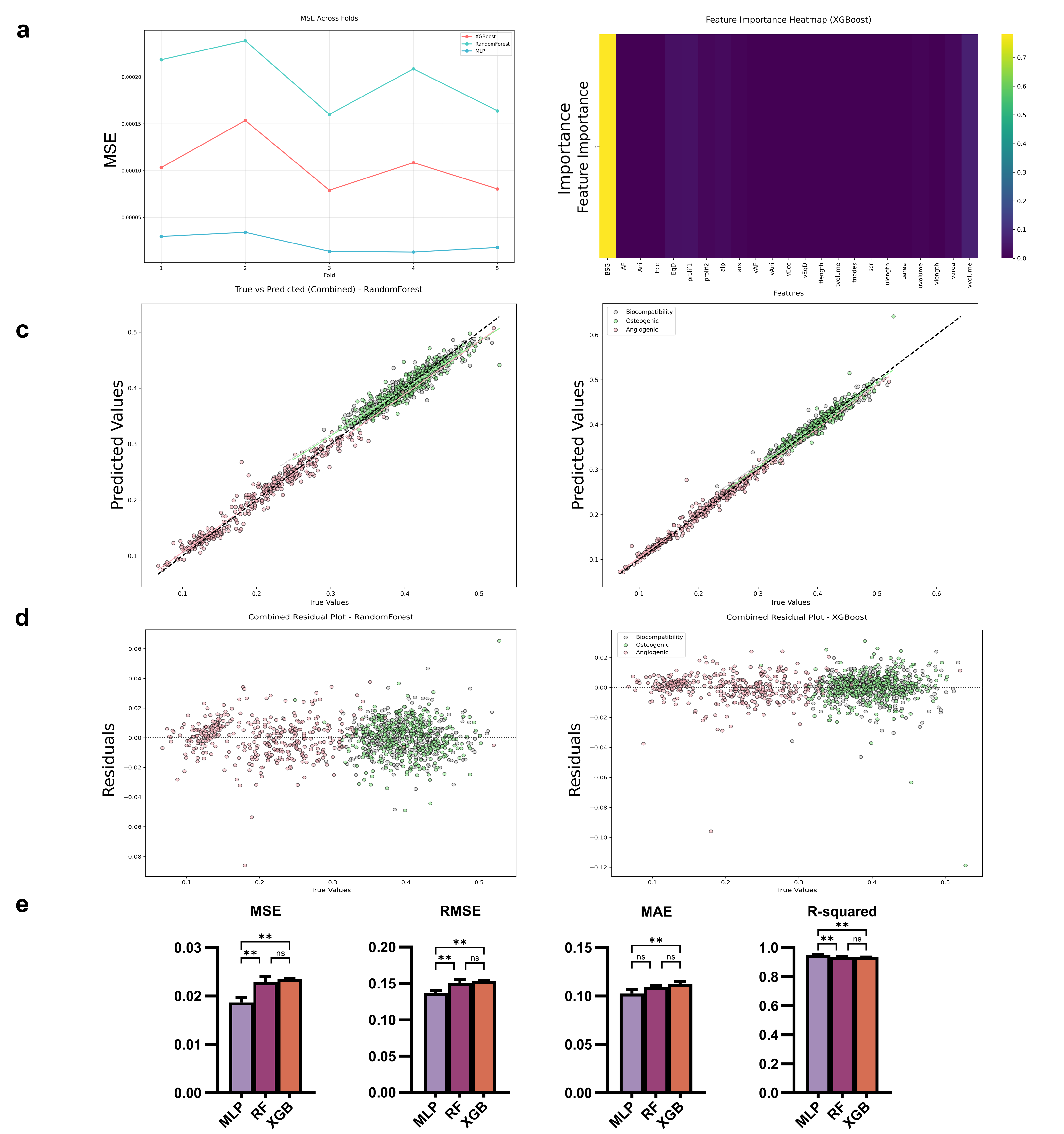

Supplement: Supplementary file 2 — Supporting File 2: advs76351‐sup‐0002‐FigureS1‐S16.zip. [file ADVS-9999-e76351-s001.zip › Fig S6.png]

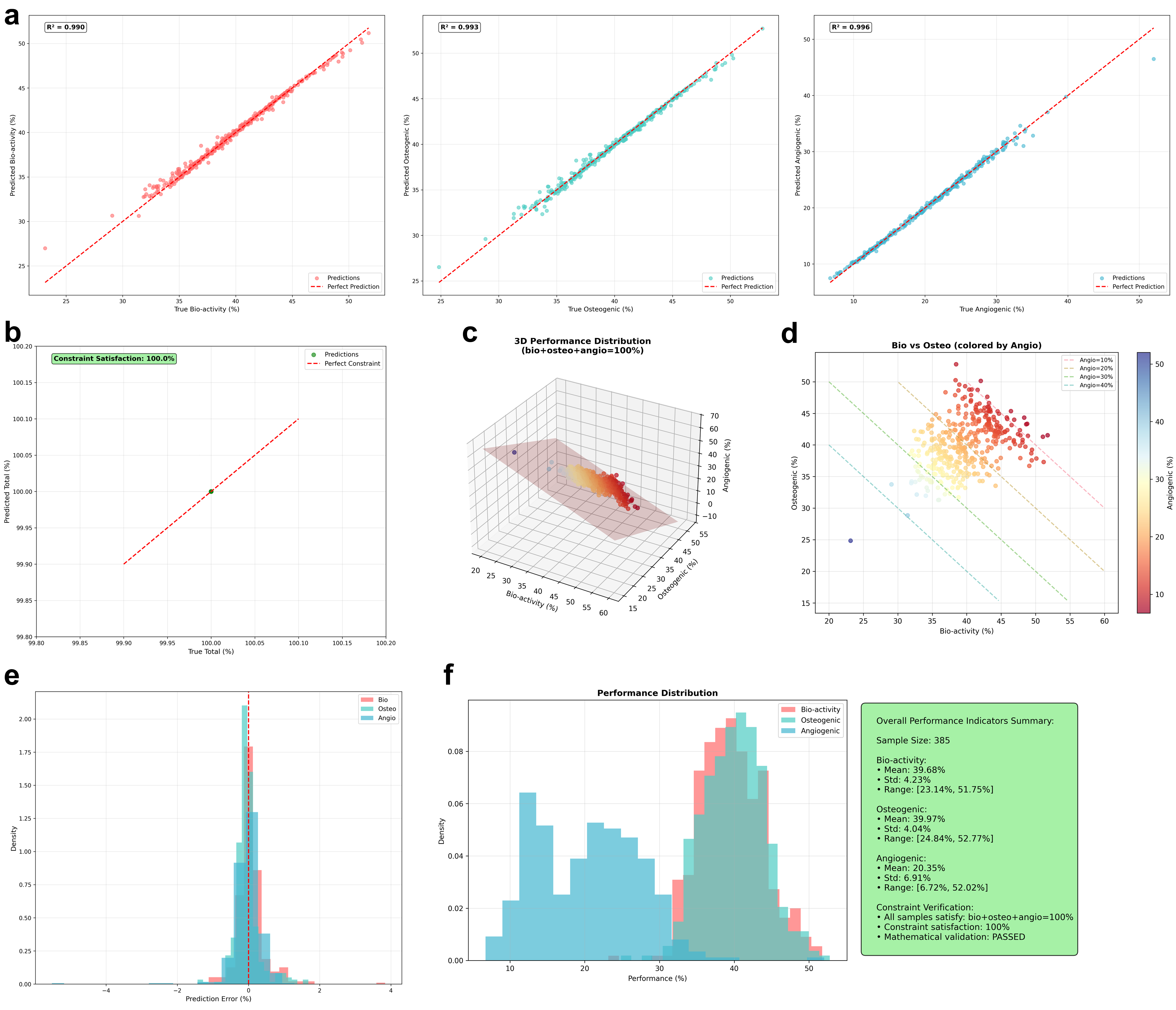

Supplement: Supplementary file 2 — Supporting File 2: advs76351‐sup‐0002‐FigureS1‐S16.zip. [file ADVS-9999-e76351-s001.zip › Fig S7.png]

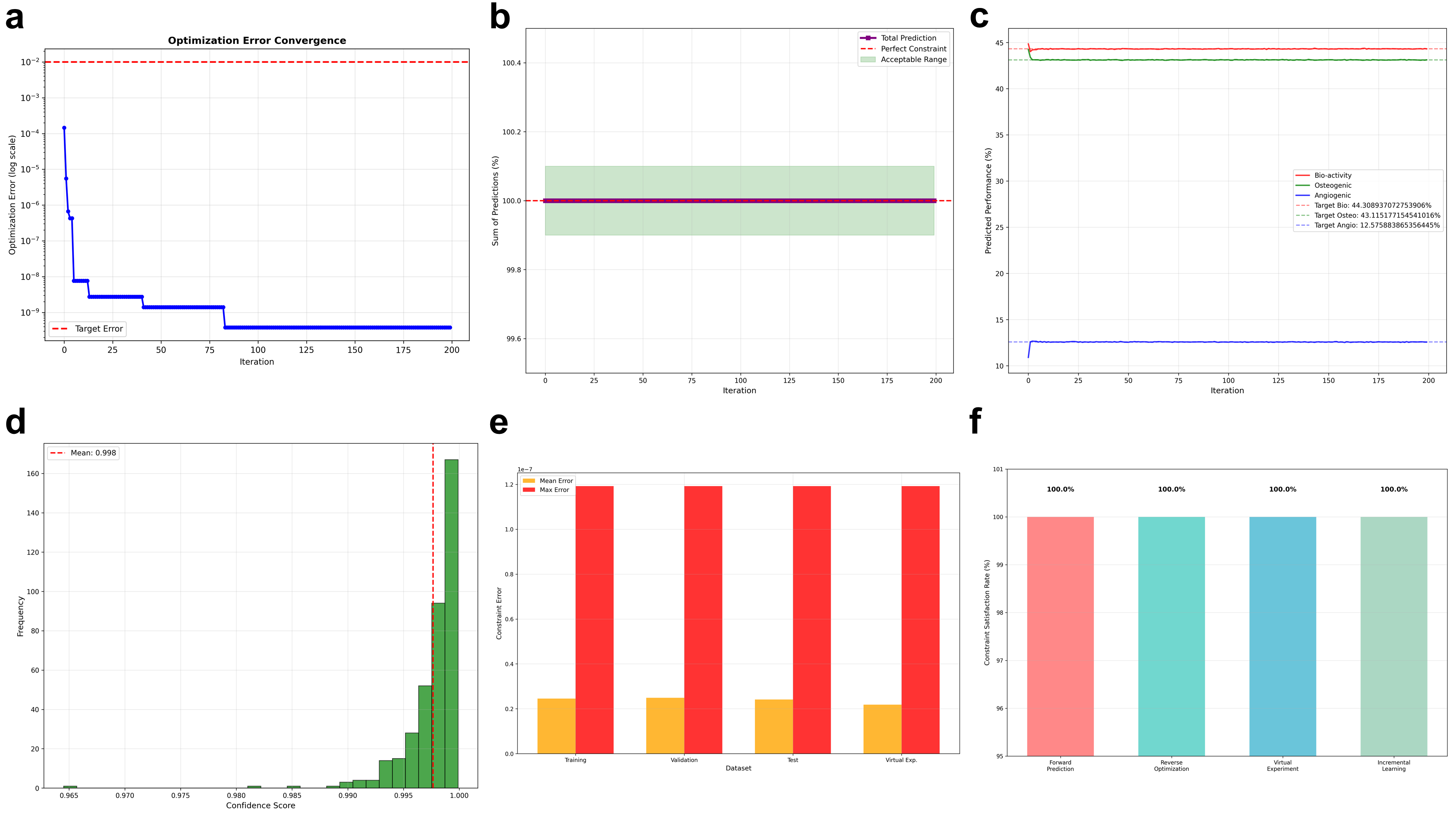

Supplement: Supplementary file 2 — Supporting File 2: advs76351‐sup‐0002‐FigureS1‐S16.zip. [file ADVS-9999-e76351-s001.zip › Fig S8.png]

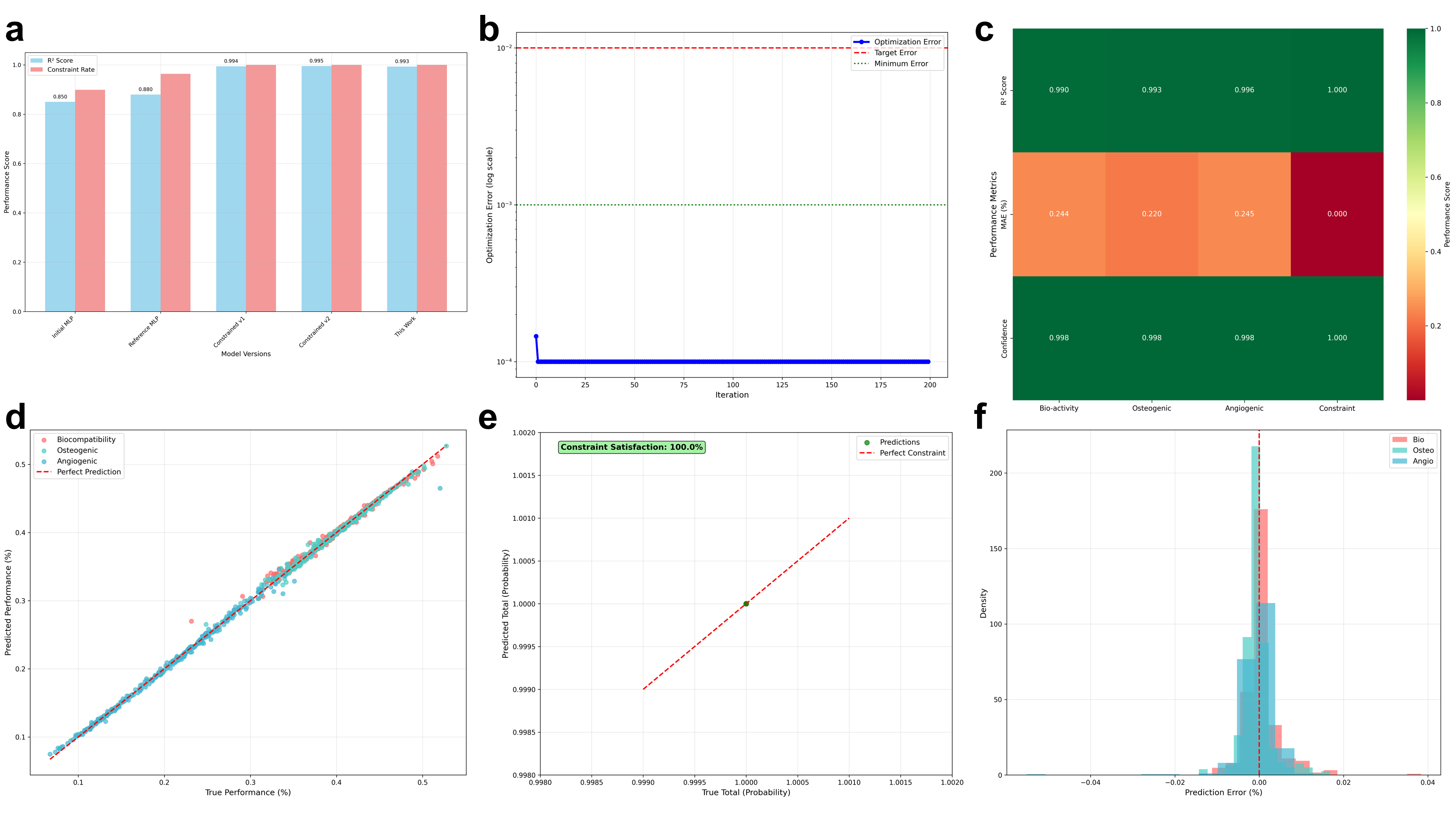

Supplement: Supplementary file 2 — Supporting File 2: advs76351‐sup‐0002‐FigureS1‐S16.zip. [file ADVS-9999-e76351-s001.zip › Fig S9.png]
